# Hospital-Wide Implementation, Clinical Outcomes, and Safety of Phenobarbital for Alcohol Withdrawal

**DOI:** 10.1001/jamanetworkopen.2025.28694

**Published:** 2025-08-25

**Authors:** Benjamin J. Wolpaw, Hannah Oren, Laura Quinnan-Hostein, Katharine A. Bradley, Nicholas J. Johnson, Kevin A. Hallgren, Ayushi Gupta, H. Nina Kim, Marion A. Granich, Tessa L. Steel

**Affiliations:** 1 Division of General Internal Medicine, Department of Medicine, University of Washington, Seattle; 2 Kaiser Permanente Washington Health Research Institute, Seattle; 3 Department of Emergency Medicine, University of Washington and Harborview Medical Center, Seattle; 4 Division of Pulmonary, Critical Care & Sleep Medicine, Department of Medicine, University of Washington and Harborview Medical Center, Seattle; 5 Department of Psychiatry and Behavioral Sciences, University of Washington and Harborview Medical Center, Seattle; 6 Department of Medicine, University of Washington, Seattle; 7 Division of Allergy & Infectious Diseases, Department of Medicine, University of Washington and Harborview Medical Center, Seattle; 8 Center for Clinical Excellence, University of Washington Medical Center, Seattle

## Abstract

**Importance:**

Phenobarbital monotherapy is increasingly used to treat alcohol withdrawal syndrome (AWS) in hospitalized patients, but its implementation outside academic emergency departments (EDs) and intensive care units (ICUs) remains understudied.

**Objective:**

To evaluate the implementation, clinical outcomes, and safety of an electronic health record (EHR) order set for intravenous weight-based phenobarbital loading (10 or 15 mg/kg) across all care locations (ED, acute care, and ICU) of a community hospital.

**Design, Setting, and Participants:**

This retrospective quality improvement study was conducted at a 281-bed community hospital in Seattle, Washington, including all hospitalized adults treated for AWS 11 months before and 12 months after implementation of the phenobarbital EHR order set (April 1, 2021, to March 31, 2023). Data analysis occurred in May 2024.

**Exposure:**

Initiation of AWS treatment before vs on or after March 24, 2022.

**Main Outcomes and Measures:**

Implementation was assessed by order set use and cumulative phenobarbital and benzodiazepine exposure. Clinical outcomes included daily maximum Clinical Institute Withdrawal Assessment of Alcohol Scale Revised (CIWA-Ar) scores, AWS treatment duration (time from first to last medication), and time from AWS treatment initiation to hospital discharge. Safety outcomes included prolonged use of physical restraints (>1 day), intubation, and in-hospital mortality. Logistic and negative binomial regression models were used to estimate differences in each outcome, before vs after implementation, adjusting for baseline group differences.

**Results:**

Among 254 patients, the preimplementation (154 patients) and postimplementation (100 patients) groups were similar in terms of baseline characteristics; most were middle-aged (mean [SD] age, 53.0 [14.9] years), male (177 men [66.9%]), and publicly insured (186 patients [73.2%]). After implementation, the phenobarbital order set was used for 67 patients (67.0%), predominantly in ED and acute care locations. AWS symptoms resolved more rapidly after implementation, with a 4.2- to 5.0-point reduction in daily maximum CIWA-Ar scores at 24 to 96 hours from hospital presentation, 30.1-hour reduction in AWS treatment duration (95% CI, 16.7-43.5 hours), and 2.2-day reduction in time to hospital discharge (95% CI, 0.7-3.7 days). Safety outcomes did not significantly differ before and after implementation.

**Conclusions and Relevance:**

In this quality improvement study, implementation of a weight-based phenobarbital order set for AWS in all care locations of a community hospital was associated with faster symptom resolution, shorter length of stay, and no differences in safety outcomes. These findings support the broader adoption of phenobarbital-based AWS treatment across diverse hospital settings.

## Key Points

**Question:**

Is implementing a phenobarbital electronic health record order set across all care locations of a community hospital associated with improved outcomes for alcohol withdrawal syndrome (AWS)?

**Findings:**

In this quality improvement study comparing outcomes before (154 patients) and after (100 patients) implementation, 67% of postimplementation patients were treated using the phenobarbital order set, which was associated with faster symptom resolution (4.2- to 5.0-point Clinical Institute Withdrawal Assessment of Alcohol Scale Revised score reduction from 24-96 hours), reduced AWS treatment duration (30.1 hours), shorter hospital stay (2.2 days), and no increase in adverse events.

**Meaning:**

These findings suggest that hospital-wide use of a phenobarbital order set is safe and effective for treating AWS and is associated with shortened symptom duration and length of stay.

## Teaser

This quality improvement study evaluates the implementation, clinical outcomes, and safety of an electronic health record order set for intravenous weight-based phenobarbital loading for treating alcohol withdrawal syndrome.

## Introduction

Benzodiazepines, the backbone of traditional alcohol withdrawal syndrome (AWS) treatment, are deliriogenic, addictive, and a leading cause of adverse drug reactions in hospitals.^1,2^ Phenobarbital has been used as an alternative for over 30 years,^3^ but has recently gained traction in US hospitals, increasing from 6.1% to 18.4% use from 2016 to 2020.^4^ The American Society of Addiction Medicine (ASAM) recommends phenobarbital as a first-line AWS treatment when prescribed by experienced practitioners.^5^

Phenobarbital offers practical advantages over benzodiazepines. A single weight-based loading dose can provide rapid and sustained symptom control, in contrast to sequential, reactive dosing often used with short-acting benzodiazepines in US hospitals.^6^ Weight-based dosing reduces reliance on subjective symptom scales, like the Clinical Institute Withdrawal Assessment of Alcohol Scale Revised (CIWA-Ar),^7^ which have barriers to appropriate use in hospitals.^8,9^ In addition, phenobarbital provides GABA (γ-aminobutyric acid) agonism and glutamate antagonism,^10,11,12^ addressing both dominant aspects of AWS pathophysiology, whereas benzodiazepines only enhance GABA signaling.^13^

A limited but growing body of evidence supports the safety and effectiveness of phenobarbital for treating AWS, specifically in academic emergency department (ED) and intensive care unit (ICU) settings,^14,15,16,17,18^ but practical implementation protocols are needed to support phenobarbital treatment in community hospitals and acute care (ie, general hospital ward) settings, where the majority of hospitalized patients with AWS receive their care.^19^ This study evaluated implementation, clinical outcomes, and safety outcomes before and after adoption of an electronic health record (EHR) order set for weight-based intravenous phenobarbital monotherapy in all care locations (ED, acute care, and ICU) of a community hospital with limited prior experience using this approach for AWS.

## Methods

### Setting

The study was conducted in the University of Washington health system (UW Medicine) from April 1, 2021, to March 31, 2023. On March 24, 2022, UW Medicine implemented a phenobarbital EHR order set for AWS across all care locations (ED, acute care, and ICU) of its 3 hospitals. Two UW Medicine hospitals were already using phenobarbital, whereas the third, a 281-bed urban community hospital that joined UW Medicine in January 2020, was not. The study focused on this third hospital, after transition of the UW Medicine EHR to Epic (Epic Systems Corporation) on April 1, 2021.

Most patients at the study hospital were cared for by hospitalists in a direct care model, without trainees or advanced practice practitioners. Before implementation of the EHR order set, most had no prior experience using phenobarbital monotherapy for AWS.

Data for the study were obtained from the UW Medicine EHR. The UW institutional review board reviewed and approved the study with a waiver of consent, because the data were deidentified, and Health Insurance Portability and Accountability Act authorization under the institution’s Federalwide Assurance. This report adheres to Strengthening the Reporting of Observational Studies in Epidemiology (STROBE) reporting guidelines and Standards for Quality Improvement Reporting Excellence (SQUIRE) 2.0 reporting guidelines.

### Sample

Eligible patients were hospitalized at least 24 hours at the study hospital and were determined to be at risk and treated for AWS during the 11 months before and 12 months after implementation of the phenobarbital order set (eFigure 1 in Supplement 1). The group size reflected actual variation in admission volume. Being at risk for AWS was defined as either an EHR order for CIWA-Ar monitoring, a positive blood or urine alcohol test within 24 hours of presentation, or an alcohol-related diagnosis within 2 years prior or the first 24 hours of hospitalization. Being treated was defined as receiving any dose of phenobarbital or 2 or more doses of benzodiazepine within a 12-hour period during the first 7 days of hospitalization. Benzodiazepines included chlordiazepoxide, diazepam, and lorazepam (others are rarely used for AWS in UW Medicine).

Patients were excluded if pregnant (less representative of general hospital patients), admitted to a psychiatric service (given frequent benzodiazepine use for non-AWS indications), transitioned to comfort-focused end-of-life care within 14 days (given benzodiazepine use for comfort rather than AWS), or encounter data were missing (eFigure 1 in Supplement 1). For patients with multiple hospitalizations during the study period, only the first was included to facilitate person-level analyses.

### Intervention: Phenobarbital EHR Order Set Implementation

#### EHR Order Set

An EHR order set (Box; eTable 1 in Supplement 1) guiding the use of weight-based intravenous phenobarbital for AWS was introduced on March 24, 2022, as part of a quality improvement initiative. The order set included 2 loading-dose options: 15 mg/kg for most patients and 10 mg/kg for those at lower risk of severe AWS or heavily pretreated with benzodiazepines. Practitioners could add as-needed phenobarbital doses (130 mg twice and 260 mg once) for uncontrolled agitation or CIWA-Ar scores 15 or higher. The order set also encouraged maintenance phenobarbital 1 to 2 mg/kg every 12 hours for 2 to 3 days.

Box. **Key Components of the Phenobarbital Order Set Implemented in the Electronic Health Record on March 24, 2022^a^**

**Key Order Set Components**

**Nursing**

- Vital signs 10 minutes after phenobarbital loading dose
- Clinical Institute Withdrawal Assessment for Alcohol Revised (CIWA-Ar) every 1-4 hours based on score

**Loading Dose**

- Phenobarbital 15 mg/kg intravenous piggyback (recommended for most patients)
- Phenobarbital 10 mg/kg intravenous piggyback (low risk or heavily pretreated with benzodiazepines)

**As-Needed Doses**

- Phenobarbital 130 mg intravenous twice as needed for uncontrolled agitation or CIWA-Ar ≥15
- Phenobarbital 260 mg intravenous once as needed for uncontrolled agitation or CIWA-Ar ≥15

^a^
Complete order set is shown in eTable 1 in Supplement 1.

#### Education and Implementation Support

To support implementation, educational presentations were delivered to staff physicians at grand rounds (November 18, 2021, 60 minutes), a hospitalist faculty meeting (March 8, 2022, 15 minutes), and an ED faculty meeting (March 17, 2022, 30 minutes). Training covered historical use, supporting literature, pharmacologic advantages, and weight-based dosing of phenobarbital (eFigure 2 in Supplement 1). A frequently asked questions document was also emailed to hospitalist, family medicine, and ED faculty (eTable 2 in Supplement 1). Postimplementation support included ad hoc consultation with quality improvement leadership and presentation of preliminary safety and outcome data to hospitalist faculty (March 9, 2023) and the substance use disorder committee (March 19, 2023).

#### Implementation Factors and Adaptations

A benzodiazepine-based AWS order set remained available in the EHR, with options for scheduled chlordiazepoxide and symptom-triggered lorazepam based on CIWA-Ar scores. Initially, practitioners were not directed to use the phenobarbital order set over the benzodiazepine-based order set; however, a systemwide intravenous benzodiazepine shortage prompted a best practice alert (eFigure 3 in Supplement 1), implemented September 15, 2022, upon opening the benzodiazepine order set, which encouraged use of the phenobarbital order set. Initially, intravenous phenobarbital required 30 minutes of telemetry monitoring for infusion-related hypotension, but this requirement was removed in January 2023 after no adverse events were observed.

### Measures

#### Outcomes

The study evaluated implementation and clinical and safety outcomes. Implementation outcomes included adoption and penetration.^20^ Adoption was defined as use of the phenobarbital EHR order set during the first 14 days of hospitalization, as well as changes in cumulative benzodiazepine and phenobarbital exposure. Penetration was assessed by order set use across care locations (ED, acute care, or ICU) and prescriber type (emergency, medical, or surgical), reflecting integration with various hospital systems of care. Clinical outcomes were assessed by symptom control, defined as the maximum CIWA-Ar score recorded during each 24-hour period for the first 4 days. In addition, time from AWS treatment initiation to (1) last dose of benzodiazepine or phenobarbital and (2) hospital discharge was used to define AWS treatment duration and time to hospital discharge, respectively. Safety outcomes included prolonged use of physical restraints (>1 calendar day within 14 days), intubation after initiation of AWS treatment (within 14 days), and in-hospital mortality. Prolonged restraint use was defined as more than 1 calendar day to distinguish from transient use (eg, for brief agitation) and considered more reflective of inadequate symptom control.

#### Exposure and Covariates

Before vs after implementation was defined as initiation of AWS treatment before vs on or after March 24, 2022. Sociodemographic characteristics documented in the EHR were used to approximate patient identities and lived experiences. These included age at admission, sex, race, ethnicity, homelessness status, and insurance type. Data on race and ethnicity are included in this study because previous studies have found variation in racial and ethnic distribution in presentations of AWS.^21^

Clinical characteristics were classified as either preexisting or acute. Preexisting morbidity was measured using the combined comorbidity score (CCS, Gagne Index, range −2 to 26), which estimates 1-year mortality risk using *International Statistical Classification of Diseases and Related Health Problems, Tenth Revision* diagnoses (eTable 3 in Supplement 1).^22,23^ The CCS was dichotomized at 5 or above, corresponding to a greater than 20% risk of 1-year mortality (ie, severe chronic morbidity).^23,24^ Prior AWS and alcohol use disorder or alcohol-related diagnoses were also evaluated. Acute illness severity was measured using the highest National Early Warning Score (range, 0-20) within 48 hours of presentation,^25^ dichotomized as mild-to-moderate (<7) or severe (≥7) to enhance interpretability.^26,27^ Additional acute diagnoses documented within 48 hours of presentation included arrhythmias; brain injuries (including trauma, hemorrhage, and stroke); gastrointestinal disorders; liver disease; nutrition, electrolyte, and acid-base disorders; other substance use disorders (excluding alcohol); psychiatric conditions; seizure; sepsis, shock, or infection; and/or trauma (excluding brain injuries) (eTable 3 in Supplement 1).

Care delivery factors included prescriber specialty (emergency, medical, or surgical) and care location (ED, acute care, or ICU) at the time of AWS treatment initiation. These were combined into 1 variable with 5 discrete categories: (1) ED prescriber in the ED, (2) medical prescriber in the ED, (3) medical prescriber in acute care, (4) surgical prescriber in acute care, and (5) medical prescriber in the ICU.

### Statistical Analysis

The sample was nested within a larger retrospective cohort evaluating AWS management in UW Medicine at large (eFigure 1 in Supplement 1). Analyses were conducted in May 2024. Descriptive statistics characterized the study sample, stratified by preimplementation vs postimplementation of the phenobarbital order set. Standardized mean differences (SMDs), calculated using the stddiff command in Stata, were used to assess balance in baseline characteristics across preimplementation and postimplementation groups. SMDs were computed as the difference in means standardized by pooled SD (continuous variables), difference in proportions standardized by pooled variance (binary variables), and overall distributional differences across categories (categorical variables). Covariates with SMD greater than 0.15 were included in the multivariable models, balancing parsimony, adequate control of confounding, and epidemiologic plausibility.^28,29^ Data-informed covariate selection using SMDs was pursued on the basis of modest group sizes, limiting statistical significance testing, and an a priori assumption that baseline characteristics would not differ over 24 months, limiting hypothesis-guided covariate selection. Statistical significance was defined as 2-sided *P* < .05.

Negative binomial regression was used to evaluate associations between order set implementation and cumulative benzodiazepine and phenobarbital use, symptom control (24-hour maximum CIWA-Ar scores), AWS treatment duration, and time to hospital discharge. Logistic regression assessed associations with binary safety outcomes: prolonged physical restraints, intubation, and in-hospital mortality. Models used complete case analysis. To enhance clinical interpretability, recycled estimates were used to estimate differences in each outcome, before vs after implementation. No corrections for multiple comparisons were applied, as all outcomes were prespecified and interpreted for clinical relevance rather than statistical significance. Analyses were conducted in Stata statistical software version 18.0 (StataCorp).

## Results

A total of 254 patients met eligibility criteria, including 154 before and 100 after implementation of the phenobarbital order set (eFigure 1 in Supplement 1). The 2 groups were generally similar in sociodemographic, clinical, and care delivery characteristics (Table 1). Most patients were middle-aged (mean [SD] age, 53.0 [14.9] years), male (177 men [66.9%]), and insured by Medicaid or Medicare (186 patients [73.2%]). Prior health care encounters were documented for 152 patients (59.8%), including 85 (55.9%) with prior alcohol use disorder or alcohol-related diagnoses, 40 (26.3%) with prior AWS, and 14 (9.2%) with severe chronic morbidity (CCS 1-year mortality risk >20%). During the first 48 hours, 25 patients (9.8%) met criteria for severe acute illness (National Early Warning Score ≥7). The most common diagnoses were nutrition, electrolyte, and acid-base disorders (57 patients [22.4%]) and sepsis, shock, or infection (41 patients [16.2%]). For 236 patients (92.9%), AWS treatment was initiated within the first 24 hours of hospitalization (Table 1). Most patients (156 patients [61.4%]) initiated AWS treatment with ED prescribers in the ED, followed by 18.5% (47 patients) with medical prescribers in acute care. During the first 24 hours of initial ED management and admission to the hospital, 192 patients (75.6%) were managed in acute care only (no ICU care). Small-to-moderate SMDs suggested higher postimplementation prevalence of homelessness, brain injury, other substance use disorder, and trauma (eTable 4 in Supplement 1); these variables were included in the adjusted regression models.

**Table 1.** Characteristics of the Study Sample, Before and After Implementation of the Phenobarbital Electronic Health Record Order Set

Characteristic	Patients, No. (%)	
	Before phenobarbital order set (n = 154)	After phenobarbital order set (n = 100)
Sociodemographic		
Age, mean (SD), y	53.1 (14.7)	52.7 (15.3)
Sex		
Female	53 (34.4)	31 (31.0)
Male	101 (65.6)	69 (69.0)
Race		
American Indian or Alaska Native	4 (2.6)	7 (7.0)
Asian or Native Hawaiian or Pacific Islander	4 (2.6)	2 (2.0)
Black	10 (6.5)	4 (4.0)
White	134 (84.2)	82 (82.0)
Multiple races	5 (3.3)	3 (3.0)
Other or unknown^a^	1 (0.7)	2 (2.0)
Hispanic or Latinx ethnicity	12 (7.8)	10 (10.0)
Documented homelessness	4 (2.6)	6 (6.0)
Insurance		
Commercial	30 (19.5)	23 (23.0)
Medicaid	74 (48.1)	44 (44.0)
Medicare	39 (25.3)	29 (29.0)
Self-pay	10 (6.5)	4 (4.0)
Tricare	1 (0.7)	0
Clinical		
Prior encounters within the health care system	96 (62.3)	56 (56.0)
Severe preexisting morbidity (Combined Comorbidity score ≥5), No./total No. (%)^b^	8/96 (8.3)	6/56 (10.7)
Prior alcohol use disorder (including in remission) or alcohol-related diagnosis, No./total No. (%)^b^	56/96 (58.3)	29/56 (51.8)
Prior AWS diagnosis, No./total No. (%)	27/96 (20.1)	13/56 (23.2)
First Clinical Institute Withdrawal Assessment for Alcohol Revised score, median (IQR)^c^	10 (6-14)	9 (4-16)
Severe acute illness (National Early Warning System score ≥7)	17 (11.0)	8 (8.0)
Hospital diagnoses		
Arrhythmia	14 (9.1)	12 (12.0)
Brain injury (trauma, hemorrhage, or stroke)	4 (2.6)	6 (6.0)
Gastrointestinal tract disorder	17 (11.0)	15 (15.0)
Liver disease	18 (11.7)	9 (9.0)
Nutrition, electrolyte, or acid-base disorder	35 (22.7)	22 (22.0)
Other substance use disorder (excluding alcohol)	9 (5.8)	11 (11.0)
Psychiatric condition	11 (7.1)	8 (8.0)
Seizure	14 (9.1)	9 (9.0)
Sepsis, shock, or infection	25 (16.2)	16 (16.0)
Trauma	6 (3.9)	10 (10.0)
Care delivery		
Initiated AWS treatment within 24 h of hospital presentation	144 (93.5)	92 (92.0)
Specialty of prescriber and hospital location at initiation of AWS treatment		
ED prescriber in the ED	97 (63.0)	59 (59.0)
Medical prescriber in the ED	18 (11.7)	12 (12.0)
Medical prescriber in acute care	27 (17.5)	20 (20.0)
Surgical prescriber in acute care	1 (0.7)	1 (1.0)
Medical prescriber in the intensive care unit	11 (7.1)	8 (8.0)
Acute care only (no intensive care) during first 24 h from hospital presentation	115 (74.7)	77 (77.0)

Abbreviations: AWS, alcohol withdrawal syndrome; ED, emergency department.

^a^ The other and unknown category includes declined to answer and unavailable or unknown.

^b^ Results limited to patients with prior health records (96 in the preimplementation group and 56 in the postimplementation group).

^c^ One hundred twenty-nine patients in the preimplementation group and 83 patients in the postimplementation group (83%) had at least 1 Clinical Institute Withdrawal Assessment for Alcohol Revised measurement.

Implementation outcomes indicated successful adoption and penetration of the phenobarbital order set. As expected, no patients received treatment via the order set before implementation, compared with 67 (67.0%) after implementation (Table 2). Before implementation, 52 patients (33.8%) received a median (IQR) of 3.7 (1.9-10.5) mg/kg of phenobarbital cumulatively, whereas after implementation, 71 (71.0%) received a median (IQR) 12.6 (9.7-16.1) mg/kg (eTable 5 in Supplement 1). In adjusted analyses, mean cumulative benzodiazepine per patient decreased by an estimated 22.4 lorazepam-equivalent mg (95% CI, 16.7-28.1 lorazepam-equivalent mg) and mean cumulative phenobarbital per patient increased by 554.7 mg (95% CI, 92.2-1017.1 mg) after implementation (Table 2). The order set was used by prescribers across all care locations (eTable 6 in Supplement 1).

**Table 2.** Implementation, Clinical Outcomes, and Safety Outcomes Before and After Implementation of the Phenobarbital Electronic Health Record Order Set

Variable	Median (IQR)		Estimated difference adjusted for baseline difference between groups, % (95% CI)	*P* value
	Before phenobarbital order set (n = 154)	After phenobarbital order set (n = 100)		
Implementation				
Phenobarbital order set use, unadjusted No. (%)	0	67 (67.0)	67.0 (58.8 to 76.1)^a^	<.001
Cumulative benzodiazepine, lorazepam-equivalent mg	12.0 (5.0 to 31.0)	1.5 (0.0 to 5.8)	−22.4 (−28.1 to −16.7)^a^	<.001
Cumulative phenobarbital, mg	0.0 (0.0 to 130.0)	767.0 (0.0 to 1124.3)	554.7 (92.2 to 1017.1)^a^	<.001
Clinical outcomes				
Symptom control				
Maximum, Clinical Institute Withdrawal Assessment for Alcohol Revised score, points				
Arrival to 24 h	15.0 (10.5 to 20.0)	13.0 (8.0 to 19.0)	−1.7 (−3.8 to 0.5)^b^	.14
24 to 48 h	10.0 (8.0 to 17.0)	6.0 (4.0 to 12.0)	−4.3 (−6.3 to −2.4)^b^	<.001
48 to 72 h	9.0 (4.0 to 13.0)	4.5 (2.0 to 8.0)	−4.2 (−6.1 to −2.3)^b^	<.001
72 to 96 h	8.0 (4.0 to 12.0)	4.0 (2.0 to 7.0)	−5.0 (−7.7 to −2.2)^b^	.001
Alcohol withdrawal syndrome treatment duration, h	57.6 (24.9 to 108.1)	30.2 (4.2 to 54.9)	−30.1 (−43.5 to −16.7)^b^	<.001
Time to hospital discharge, d	4.8 (2.8 to 7.4)	3.2 (2.0 to 5.8)	−2.2 (−3.7 to −0.7)^b^	.004
Safety, unadjusted No. (%)				
Prolonged physical restraints (>1 d)^c^	18 (11.7)	8 (8.0)	−5.1 (−12.4 to 2.1)^a^	.20
Intubation^c^	11 (7.1)	4 (4.0)	−4.8 (−10.5 to 1.0)^a^	.14
In-hospital death	6 (3.9)	4 (4.0)	−0.3 (−5.5 to 4.9)^a^	.92

^a^ Logistic regression model adjusted for baseline group differences (standardized mean difference >0.15) regarding homelessness, brain injury, other substance use disorder excluding alcohol, and trauma (see eTable 4 in Supplement 1).

^b^ Negative binomial regression model adjusted for baseline group differences (standardized mean difference >0.15) regarding homelessness, brain injury, other substance use disorder excluding alcohol, and trauma (see eTable 4 in Supplement 1).

^c^ During first 14 days from hospital presentation.

**Figure 1.**
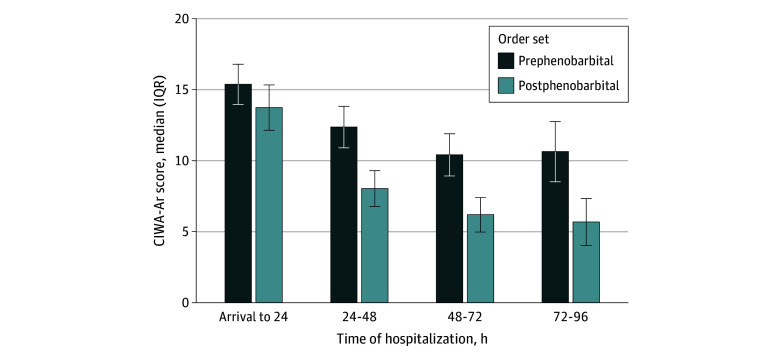
Daily Maximum Clinical Institute Withdrawal Assessment for Alcohol Revised (CIWA-Ar) Scores Before and After Implementation of the Phenobarbital Electronic Health Record Order Set, Adjusted for Baseline Differences Between Groups Depicted results are recycled estimates from the negative binomial regression model adjusting for baseline group differences (standardized mean difference >0.15) regarding homelessness, brain injury, other substance use disorder excluding alcohol, and trauma (see eTable 4 in Supplement 1). Error bars denote IQRs.

**Figure 2.**
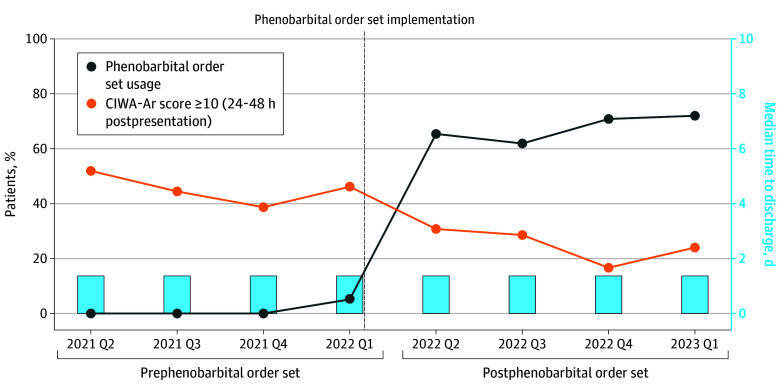
Temporal Trends in the Proportion of Patients Treated With the Phenobarbital Electronic Health Record Order Set, the Proportion With Elevated Clinical Institute Withdrawal Assessment for Alcohol Revised (CIWA-Ar) Scores (≥10) at 24 to 48 Hours, and the Median Time From Alcohol Withdrawal Syndrome Treatment Initiation to Hospital Discharge On September 15, 2022, a national benzodiazepine shortage prompted addition of a best practice alert upon opening the benzodiazepine order set, encouraging use of the phenobarbital order set. Q indicates quarter.

Significant improvements in clinical outcomes were observed after implementation (Table 2). CIWA-Ar scores were similar between groups in the first 24 hours, reflecting comparable AWS severity at presentation, but were significantly lower after implementation on subsequent days of treatment (Table 2 and Figure 1). Adjusted analyses estimated a 4.2- to 5.0-point reduction in maximum daily CIWA-Ar scores from 24 to 96 hours. AWS treatment duration was 30.1 hours (95% CI, 16.7-43.5 hours) shorter, and time from AWS treatment initiation to hospital discharge was 2.2 days (95% CI, 0.7-3.7 days) shorter after implementation. Temporal trends in CIWA-Ar scores, time to hospital discharge, and order set use are shown in Figure 2.

Safety outcomes—prolonged restraints, intubation, and in-hospital mortality—did not differ significantly between groups (Table 2). Adjusted analyses showed trends favoring the postimplementation group: 5.1% lower probability of prolonged restraints (95% CI, −12.0% to 2.1%) and 4.8% lower probability of intubation (95% CI, −10.5% to 1.0%). In-hospital mortality was rare in both groups (6 patients [3.9%] in the preimplementation group vs 4 patients [4.0%] in the postimplementation group).

## Discussion

This quality improvement study evaluated the implementation, clinical outcomes, and safety of an EHR order set protocolizing phenobarbital monotherapy for AWS in a community hospital with limited prior experience using this approach. Implementation of the phenobarbital order set was associated with significant uptake (67.0% after implementation) across all care locations (ED, acute care, and ICU), reduced benzodiazepine use (−22.4 lorazepam-equivalent mg), and increased phenobarbital use (554.7 mg). After implementation, AWS symptom control (after day 0), treatment duration, and time to discharge all improved significantly, with no evidence of adverse safety effects; rather, trends suggested potential reductions in prolonged use of physical restraints and intubation, although estimates did not reach statistical significance.

This study contributes novel findings in support of hospital-wide implementation of phenobarbital monotherapy for AWS, beyond academic ED and ICU settings. Although 2 randomized clinical trials^14,15^ and multiple quasi-experimental observational studies have supported intravenous weight-based phenobarbital for AWS in academic EDs^30,31,32,33^ and ICUs,^4,17,18,34,35,36,37,38,39^ this approach has not been evaluated in community-based acute care settings. Published acute care protocols have relied on complicated regimens of intramuscular, oral, or smaller, fractionated intravenous doses of phenobarbital to mitigate theoretical risk of infusion-related hypotension.^40,41,42^ However, findings from this study suggest that weight-based intravenous loading (10 or 15 mg/kg given over 30 minutes) is safe and associated with improved clinical outcomes for AWS in acute care settings without intensive monitoring. The results align with prior ED and ICU studies showing phenobarbital is associated with reduced use of physical restraints^16,17^ and intubation^16,17,18,35,39,43,44^ compared with benzodiazepine-based treatment regimens. Also consistent with recent data,^45^ death in this general hospital sample was rare (approximately 4% of patients in both treatment groups).

In addition to reassuring safety outcomes, phenobarbital monotherapy was associated with improved clinical outcomes. Consistent with ICU-based studies, implementation of the phenobarbital order set was associated with reduced AWS symptom severity^18,43^ and shorter hospital stays^16,17,18,35,39,44^ compared with preexisting benzodiazepine-based treatment. Prior studies that failed to show reduced length of stay had smaller samples and/or used lower phenobarbital doses or combination therapy with benzodiazepines rather than monotherapy.^34,37,38^ Given that AWS complicates approximately 1 million US hospitalizations annually,^19,21,46^ a 2-day reduction in length of stay—as observed in this study—could yield $6 billion in annual savings (assuming $3000 per inpatient day).^47^ These findings underscore the potential clinical and economic value of standardized phenobarbital protocols for AWS.

The intervention described—an EHR order set protocolizing phenobarbital monotherapy for AWS—offers a scalable model for other hospitals considering new or modified treatments for AWS. Previous qualitative research has highlighted the benefits of weight-based dosing and phenobarbital’s long half-life in reducing clinician workload compared with symptom-triggered benzodiazepine regimens.^16^ Although clinician perspectives were not assessed in this study, high postimplementation use of the order set suggests strong acceptance. Although ASAM guidelines recommend phenobarbital as a first-line treatment for AWS when prescribed by experienced practitioners,^5^ this study suggests that practitioners with limited experience can administer weight-based intravenous phenobarbital safely and effectively when guided by a structured order set.

Phenobarbital is increasingly being adopted as a treatment for AWS in US hospitals,^4^ but dosing practices remain variable.^44^ This study supports a simple protocol using 10 or 15 mg/kg, but further research is needed to compare different dosing strategies. In addition, the lack of head-to-head randomized clinical trials comparing phenobarbital monotherapy with traditional benzodiazepine-based regimens remains a critical gap in the literature.

### Limitations

This study’s preimplementation and postimplementation design limits causal inference. Although significant improvements were observed across multiple outcomes following order set implementation, external factors, such as broader trends in hospital care, must be considered. More robust analytic approaches (difference-in-differences or interrupted time-series) were not feasible owing to the absence of suitable comparator hospitals (ie, differences in baseline practices, quality improvement efforts, and prior experience with phenobarbital) and the study’s relatively short preimplementation observation period, which was limited by transition to a new EHR system. Models adjusted for baseline group differences, but residual confounding remains possible given the observational design and reliance on EHR-derived data. A benzodiazepine shortage during the study may have increased use of the phenobarbital order set, independently of practitioner preference. A Hawthorne effect is also possible, although practitioners were unaware of planned analyses, reducing the likelihood that this effect could account for the observed improvements. The study was conducted at a single urban community hospital, in a predominantly White, non-Hispanic population, which may limit generalizability. Given known geographic and sociodemographic variation in AWS prevalence, care, and outcomes,^6,19,48,49^ additional research in diverse hospital settings is needed.

## Conclusions

In this quality improvement study, implementation of an EHR order set for phenobarbital monotherapy in a community hospital was associated with high uptake, improved clinical outcomes, and no difference in safety outcomes compared with benzodiazepine-based treatment. The findings indicated that weight-based intravenous phenobarbital was associated with positive clinical and safety outcomes in the treatment of AWS, not only in ED and ICU locations, but also in acute care settings, supporting its broader adoption in hospital-based AWS management.

## Notes

Supplement 1. **eFigure 1.** Flow diagram of pre- and post-phenobarbital orderset study sample using data from a larger two-year retrospective cohort study of hospitalized patients with AWS in the UW Medicine health system **eTable 1.** Complete version of the phenobarbital orderset implemented in the electronic health record at the study site community hospital on March 24, 2022 **eFigure 2.** Educational slides presented to hospitalist faculty pre-implementation of the phenobarbital EHR orderset for AWS **eTable 2.** A frequently asked questions (FAQ) document distributed by email to Hospitalist, Family Medicine and Emergency Department faculty pre-implementation of the phenobarbital EHR orderset for AWS **eFigure 3.** Best Practice Alert prompted by opening the UW Medicine benzodiazepine-based orderset for AWS implemented on September 15, 2022 in response to an intravenous benzodiazepine shortage **eTable 3.** ICD-10 Codes used to define the sample, acute/inpatient diagnoses, pre-existing/chronic diagnoses for the Combined Comorbidity Score (CCS), and other pre-existing/chronic diagnoses relevant to treatment of AWS **eTable 4.** Standardized mean differences in characteristics of pre- and post-phenobarbital orderset groups—used to identify covariates for inclusion in multivariable regression models (i.e., analyses adjusted for baseline group differences) **eTable 5.** Use of phenobarbital pre- and post-implementation of the phenobarbital EHR orderset **eTable 6.** Use of the phenobarbital EHR orderset post-implementation by specialty of prescriber and hospital location

Supplement 2.
Data Sharing Statement
