# Supplementary material for: Hospital-Wide Implementation, Clinical Outcomes, and Safety of Phenobarbital for Alcohol Withdrawal
Source: JAMA Netw Open. 2025 Aug 25;8(8):e2528694. doi: 10.1001/jamanetworkopen.2025.28694 (PMC12379078; doi:10.1001/jamanetworkopen.2025.28694)
Supplement: Supplement 1. — eFigure 1. Flow diagram of pre- and post-phenobarbital orderset study sample using data from a larger two-year retrospective cohort study of hospitalized patients with AWS in the UW Medicine health system eTable 1. Complete version of the phenobarbital orderset implemented in the electronic health record at the study site community hospital on March 24, 2022 eFigure 2. Educational slides presented to hospitalist faculty pre-implementation of the phenobarbital EHR orderset for AWS eTable 2. A frequently asked questions (FAQ) document distributed by email to Hospitalist, Family Medicine and Emergency Department faculty pre-implementation of the phenobarbital EHR orderset for AWS eFigure 3. Best Practice Alert prompted by opening the UW Medicine benzodiazepine-based orderset for AWS implemented on September 15, 2022 in response to an intravenous benzodiazepine shortage eTable 3. ICD-10 Codes used to define the sample, acute/inpatient diagnoses, pre-existing/chronic diagnoses for the Combined Comorbidity Score (CCS), and other pre-existing/chronic diagnoses relevant to treatment of AWS eTable 4. Standardized mean differences in characteristics of pre- and post-phenobarbital orderset groups—used to identify covariates for inclusion in multivariable regression models (i.e., analyses adjusted for baseline group differences) eTable 5. Use of phenobarbital pre- and post-implementation of the phenobarbital EHR orderset eTable 6. Use of the phenobarbital EHR orderset post-implementation by specialty of prescriber and hospital location [file jamanetwopen-e2528694-s001.pdf]

Supplemental Online Content

Wolpaw BJ, Oren HO, Quinnan-Hostein et al. Hospital-wide implementation, clinical outcomes, and safety of phenobarbital for alcohol withdrawal. *JAMA Netw Open*. 2025;8(8):e2528694.
doi:10.1001/jamanetworkopen.2025.28694 2025.

eFigure 1. Flow diagram of pre- and post-phenobarbital orderset study sample using data from a larger two-year retrospective cohort study of hospitalized patients with AWS in the UW Medicine health system

eTable 1. Complete version of the phenobarbital orderset implemented in the electronic health record at the study site community hospital on March 24, 2022

eFigure 2. Educational slides presented to hospitalist faculty pre-implementation of the phenobarbital EHR orderset for AWS

eTable 2. A frequently asked questions (FAQ) document distributed by email to Hospitalist, Family Medicine and Emergency Department faculty pre-implementation of the phenobarbital EHR orderset for AWS

eFigure 3. Best Practice Alert prompted by opening the UW Medicine benzodiazepine-based orderset for AWS implemented on September 15, 2022 in response to an intravenous benzodiazepine shortage

eTable 3. *ICD-10* Codes used to define the sample, acute/inpatient diagnoses, pre-existing/chronic diagnoses for the Combined Comorbidity Score (CCS), and other pre-existing/chronic diagnoses relevant to treatment of AWS

eTable 4. Standardized mean differences in characteristics of pre- and post-phenobarbital orderset groups—used to identify covariates for inclusion in multivariable regression models (i.e., analyses adjusted for baseline group differences)

eTable 5. Use of phenobarbital pre- and post-implementation of the phenobarbital EHR orderset

eTable 6. Use of the phenobarbital EHR orderset post-implementation by specialty of prescriber and hospital location

© 2025 Wolpaw BJ et al. *JAMA Network Open*.

eFigure 1. Flow diagram of pre- and post-phenobarbital orderset study sample using data from a larger two-year retrospective cohort study of hospitalized patients with AWS in the

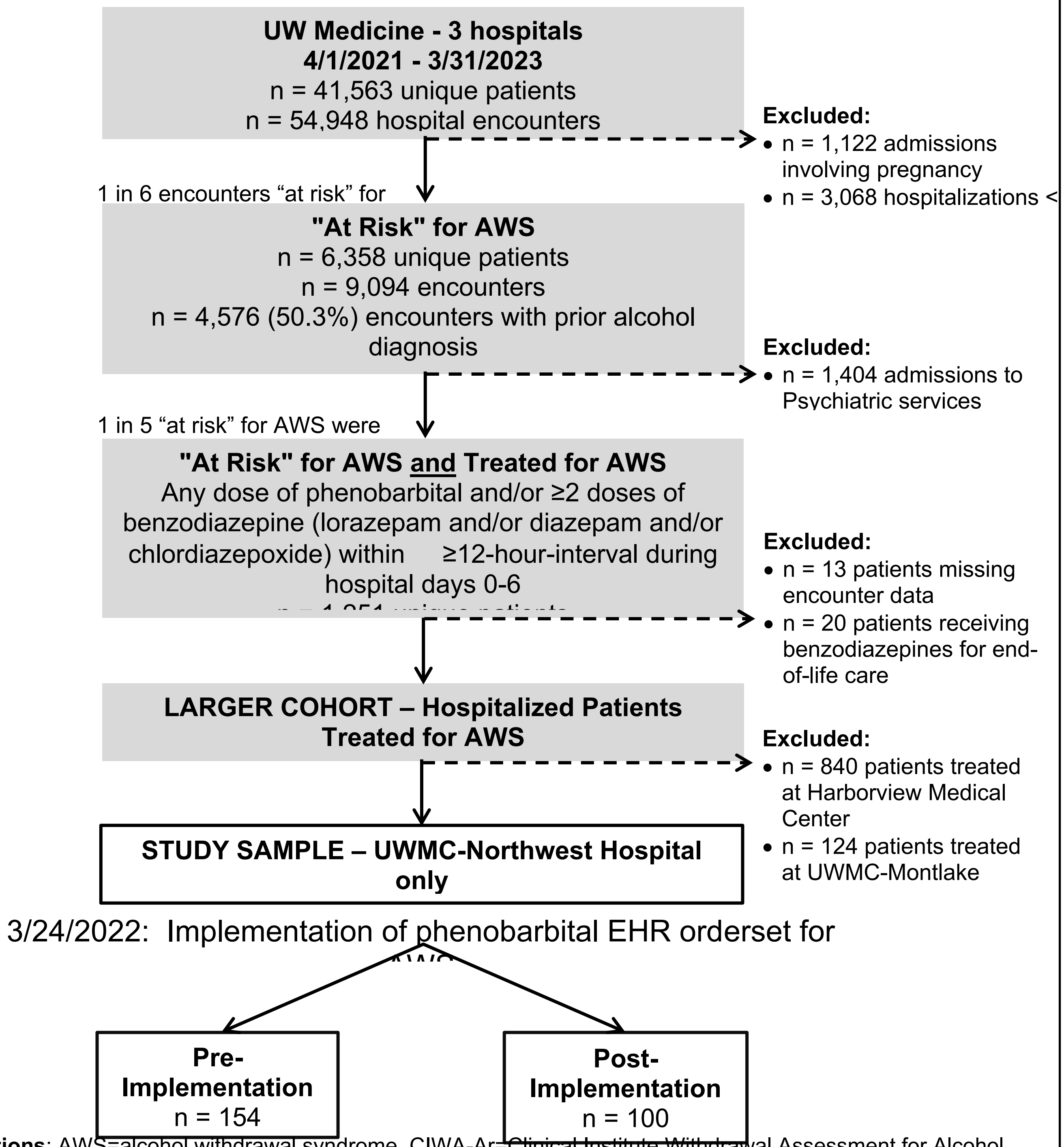

Abbreviations: AWS=alcohol withdrawal syndrome, CIWA-Ar=Clinical Institute Withdrawal Assessment for Alcohol Revised, UW=University of Washington

Timing of this pre-post study aligned within a larger retrospective cohort study of hospitalized adults receiving treatment for AWS across all UW Medicine hospitals between April 1, 2021 and March 31, 2023. The inclusion and exclusion criteria were refined using systematic chart review. For this pre-post study, the subset of patients hospitalized at the study site community hospital during the 11 months before and 12 months after implementation of the phenobarbital orderset (March 24, 2022) were included.

© 2025 Wolpaw BJ et al. *JAMA Network Open*.

eTable 1. Complete version of the phenobarbital orderset implemented in the electronic health record at the study site community hospital on March 24, 2022

Phenobarbital for Alcohol Withdrawal Syndrome (AWS)

Do not use with other alcohol withdrawal orders
Do not order benzodiazepines concurrently
Protocol uses ideal body weight not actual body weight.

Side effects and contraindications:
Phenobarbital toxicity typically begins at serum levels >40 ug/dL. The most concerning sign of toxicity is respiratory depression (low respiratory rate).

If anticipating a patient will discharge from the hospital shortly after receiving phenobarbital, they should be counseled to abstain from alcohol as a return to drinking after phenobarbital loading can heighten the toxic effects of alcohol.

General

[ ] Fall Precautions
[ ] Seizure precautions

Nursing

Vital signs

- 10 minutes after start of phenobarbital loading dose infusion and per unit standard
- 5 minutes after phenobarbital IV push doses
- With every CIWA Assessment
  - Assess and record RASS or CIWA score PRN with minimum assessment Q4h
- Notify provider AND STAT RN if:
  - CIWA score ≥20 or RASS ≥+2
  - Phenobarbital level ≥40 ug/mL
- Hold phenobarbital and notify provider if RR <10 , SBP <100 mmHg, or does not rouse easily to voice
- CIWA Assessment
  1) If CIWA is 10 or LESS, perform CIWA assessment Q4H while awake
  2) If CIWA is 11 to 14, perform CIWA assessment Q2H
  3) If CIWA is 15 or GREATER, perform CIWA assessment Q1H

Studies

See attached document for further discussion of the use of phenobarbital level. Level is not necessary prior to redosing for clinically uncontrolled withdrawal.

[X] Phenobarbital Level
AM lab x2

Medications

ORDERING GUIDELINES

1. Choose loading dose according to perceived risk (higher or lower) for severe AWS
   a. Higher risk for severe AWS if:
      i. h/o ICU admission for AWS
      ii. h/o alcohol withdrawal seizures or delirium tremens
      iii. BAL > 200 mg/dL and showing signs of AWS
2. Re-dose with 130-260mg Q10minutes PRN for clinical concern for uncontrolled AWS up to a total of cumulative dose of 30mg/kg IBW.
3. Choose maintenance dose according to perceived risk (higher or lower) for severe AWS and ongoing symptoms of AWS following the phenobarbital load

© 2025 Wolpaw BJ et al. *JAMA Network Open*.

Phenobarbital Initial Treatment [make single select so providers can/must only order one]

[ ] Phenobarbital (loading dose for higher risk of severe AWS)
15 mg/kg (IBW), Intravenous, Administer over 30 minutes, ONCE

[ ] Phenobarbital (loading dose for lower risk of severe AWS or heavily pre-treated with benzodiazepines)
10 mg/kg (IBW), Intravenous, Administer over 30 minutes, ONCE

Phenobarbital Additional Treatment

[X] Phenobarbital (Additional dose #1, prn)
130mg IVPB ONCE
Comment: RN to administer 10 minutes AFTER previous dose IF uncontrolled agitation or CIWA-Ar ≥ 15 or RASS ≥ +1 (if in ICU). Notify provider.

[X] Phenobarbital (Additional dose #2, prn)
130mg IVPB ONCE
Comment: RN to administer 10 minutes AFTER previous phenobarbital dose IF uncontrolled agitation or CIWA-Ar ≥ 15 or RASS ≥ +1 (if in ICU). Notify provider.

[X] Phenobarbital (Additional dose #3, PRN)
260mg IVPB ONCE
Comment: administer 10 minutes AFTER previous dose IF uncontrolled agitation or CIWA-Ar ≥ 15 or RASS ≥ +1 (if in ICU). Notify provider.

Phenobarbital Maintenance Dosing

[ ] Phenobarbital (maintenance dose for higher risk of severe AWS)
2 mg/kg (IDEAL) PO BID for 6 doses start Day 2

[ ] Phenobarbital (maintenance dose for lower risk of severe AWS)
1 mg/kg (IDEAL) PO BID for 4 doses start Day 2

Antiemetics

[ ] Ondansetron 4-8mg IV, EVERY 8 HOURS PRN, Nausea, Vomiting

Thiamine / Folic Acid / Multivitamin

[X] Thiamine (if evidence of malnutrition)
500mg IV TID x 3 days, give prior to any food or dextrose containing IVF

[ ] Thiamine (if no evidence of malnutrition)
100mg IV DAILY x3 days, give prior to any food or dextrose containing IVF

[X] Thiamine 100mg PO Daily starting on Day 4

[X] MVI c/ Folic acid tablet 1 tab PO DAILY

Abbreviations: AWS=alcohol withdrawal syndrome, BAL=blood alcohol level, BID=Bis in Die (twice a day), CIWA-Ar=Clinical Institute Withdrawal Assessment for Alcohol Revised, ICU=intensive care unit, IBW=ideal body weight, IV=intravenous, IVPB=intravenous piggyback, IVF=intravenous fluid, MVI=multivitamin, PO=per os (by mouth), PRN=pro re nata (as needed), RASS=Richmond Agitation-Sedation Scale, RN=registered nurse, RR=respiratory rate, SBP=systolic blood pressure

© 2025 Wolpaw BJ et al. *JAMA Network Open*.

eFigure 2. Educational slides presented to hospitalist faculty pre-implementation of the phenobarbital EHR orderset for AWS

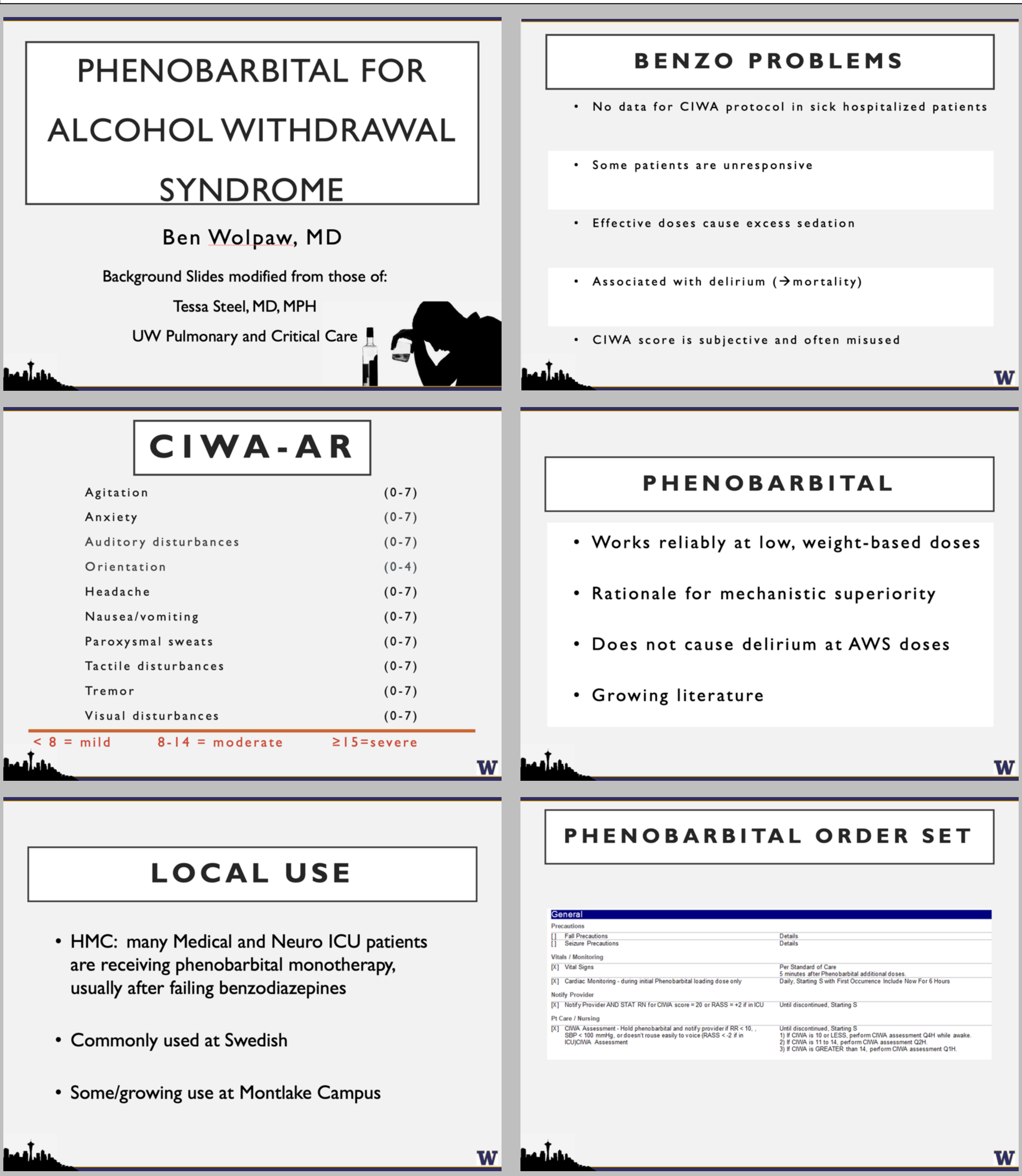

© 2025 Wolpaw BJ et al. *JAMA Network Open*.

eFigure 2. Educational slides presented to Hospitalist faculty pre-implementation of the phenobarbital EHR orderset for AWS (continued)

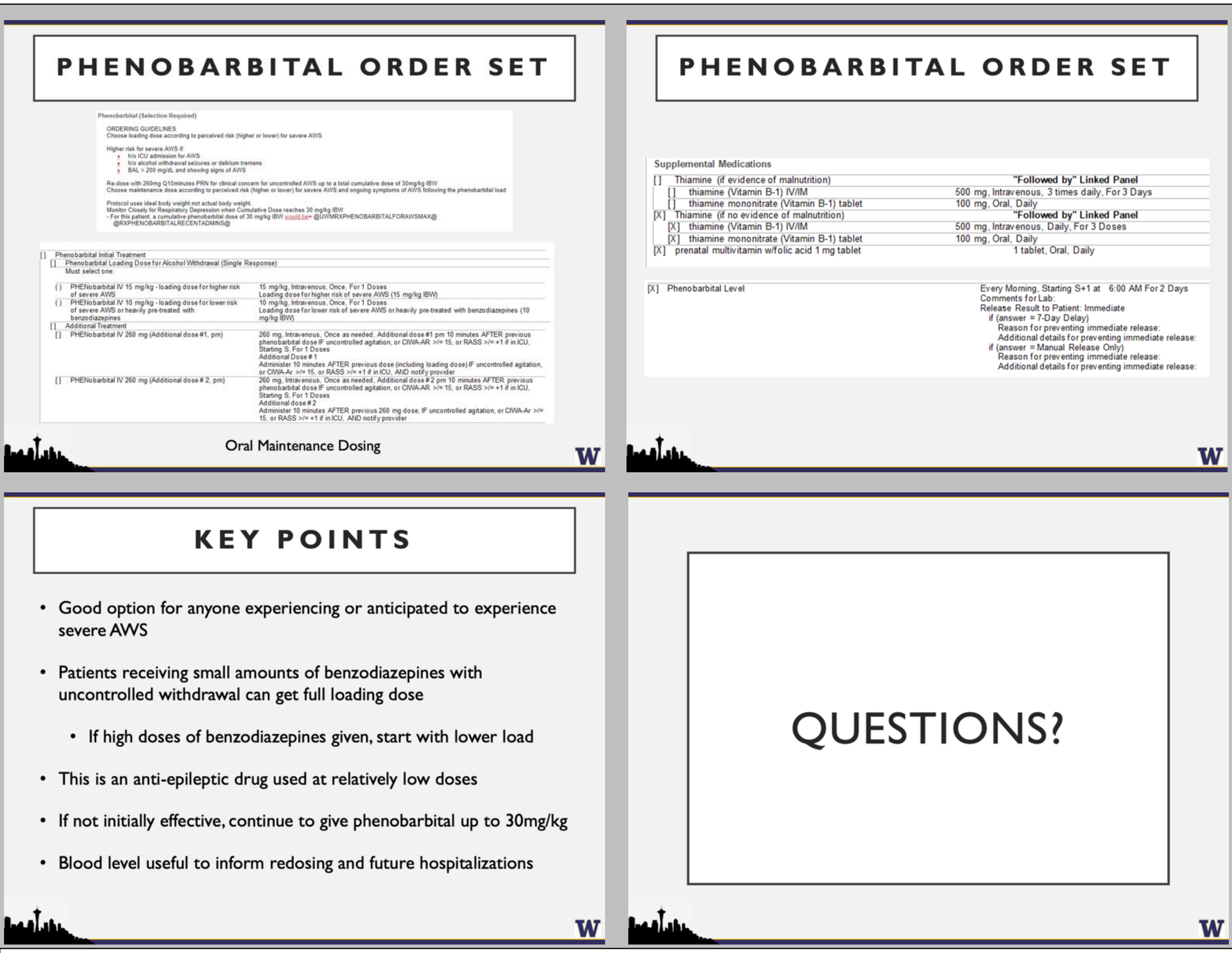

Abbreviations: AWS=alcohol withdrawal syndrome, BAL=blood alcohol level, BID=Bis in Die (twice a day), CIWA-Ar=Clinical Institute Withdrawal Assessment for Alcohol Revised, EHR=electronic health record, ICU=intensive care unit, IBW=ideal body weight, IV=intravenous, IVPB=intravenous piggyback, IVF=intravenous fluid, Montlake=University of Washington Medical Center-Montlake, MVI=multivitamin, PO=per os (by mouth), PRN=pro re nata (as needed), RASS=Richmond Agitation-Sedation Scale, RN=registered nurse, RR=respiratory rate, SBP=systolic blood pressure, Swedish=Seattle-area health system affiliated with Providence St. Joseph Health, TID=ter in die (three times a day)

© 2025 Wolpaw BJ et al. *JAMA Network Open*.

eTable 2. A frequently asked questions (FAQ) document distributed by email to Hospitalist, Family Medicine, and Emergency Department faculty pre-implementation of the phenobarbital EHR orderset for AWS

Who should get phenobarbital monotherapy for alcohol withdrawal?	This is an option for any patient who requires treatment for alcohol withdrawal syndrome (American Society for Addiction Medicine recommends either phenobarbital OR benzodiazepines as first line therapy). This protocol is *particularly* well-suited to patients at high risk for delirium with relative contraindications to benzodiazepine therapy (cirrhosis, older age, encephalopathy), or severe withdrawal physiology and benzodiazepine resistance (e.g., >40 mg of diazepam or >4 mg of lorazepam in one hour). The only true contraindication to the relatively low doses of phenobarbital used for alcohol withdrawal (compared to what is used for epilepsy) is allergy to barbiturates. Close monitoring is advised in cases of decompensated cirrhosis, severely impaired hepatic function, and respiratory failure without mechanical ventilation. However, in these cases, the risks of benzodiazepines are equivalent (or greater). In higher risk situations, a lower initial load of phenobarbital is advised: assess effect, then reload as needed.
Can patients be discharged if they have received phenobarbital?	Yes. The concern is that if someone drinks alcohol with phenobarbital in their system, the toxic effects of alcohol can be exaggerated. The same is true for patients who are discharged on chlordiazepoxide or diazepam. Patients need to be counseled that if they do drink, the effects of alcohol will be more pronounced. Patients who are not able to comply with discharge recommendations are not good candidates for discharge on phenobarbital (or any outpatient benzodiazepine). If a patient does discharge on phenobarbital, an oral maintenance dose is optional but not necessary as the long half-life of phenobarbital reduces the risk of recurrent withdrawal.
What should you look out for in a patient who has received phenobarbital?	Phenobarbital toxicity occurs primarily at serum levels >40 mg/dL, which is MUCH higher than the rough target of ~15-20 mg/dL used for alcohol withdrawal. Signs of toxicity at high serum levels include somnolence, ataxia, nystagmus, confusion, slurred speech, respiratory depression, and hypotension. After receiving phenobarbital, it is NORMAL for patients to sleep deeply for several hours. Their respiratory rate should be normal and they should still protect their airway, but they may not wake to voice or light stimulus. The respiratory rate is key: if normal, the patient's respiratory drive is not depressed. Those with obstructive sleep apnea may still obstruct their upper airway, just as they do during sleep at baseline.
What do you do if a patient has uncontrolled withdrawal after the phenobarbital loading dose has been given?	Give more phenobarbital! Give 260mg IV about every 15 minutes until the patient is calm/asleep, as long as cumulative dosing is less than 30mg/kg. If you have reached this limit and the patient's alcohol withdrawal is not controlled (rare), alternative sources of agitated delirium and hyperautonomia should be considered (e.g., head trauma, other intoxication or withdrawal syndrome, serotonin syndrome, neuroleptic malignant syndrome). The patient should be managed in an ICU with adjuvants such as dexmedetomidine, ketamine, and if needed, intubation and propofol infusion.

Abbreviations: AWS=alcohol withdrawal syndrome, EHR=electronic health record, ICU=Intensive Care Unit, IV=intravenous

© 2025 Wolpaw BJ et al. *JAMA Network Open*.

eFigure 3. Best Practice Alert prompted by opening the UW Medicine benzodiazepine-based orderset for AWS, implemented on September 15, 2022 in response to an intravenous benzodiazepine shortage

LORAZEPAM SHORTAGE ALERT

Due to a national injectable lorazepam shortage, consider the following:

1. Phenobarbital is an alternative treatment option – consider using the *'Phenobarbital Protocol for Alcohol Withdrawal Syndrome'* order set instead.
2. When using this order set, consider selecting oral benzodiazepines and/or phenobarbital 130 mg IV push.
3. Lorazepam inj has been replaced with diazepam inj in this order set. Monitor for

✓ OK

Abbreviations: AWS=alcohol withdrawal syndrome, inj=injection dose, UW=University of Washington

Initially, providers were not directed to use the new phenobarbital orderset over the existing benzodiazepine-based orderset; however, a national intravenous benzodiazepine shortage prompted the addition of a best practice alert

© 2025 Wolpaw BJ et al. *JAMA Network Open*.

eTable 3. *ICD-10* Codes used to define the sample, acute/inpatient diagnoses, pre-existing/chronic diagnoses for the Combined Comorbidity Score (CCS), and other pre-existing/chronic diagnoses relevant to treatment of AWS

Study Measure	ICD-10 Codes
Sample definition	
Alcohol-related disorders	F10.10, F10.120, F10.121, F10.129, F10.130, F10.131, F10.132, F10.139, F10.14, F10.150, F10.151, F10.159, F10.180, F10.181, F10.182, F10.188, F10.19, F10.20, F10.220, F10.221, F10.229, F10.230, F10.231, F10.232, F10.239, F10.24, F10.250, F10.251, F10.259, F10.26, F10.27, F10.280, F10.281, F10.282, F10.288, F10.29, F10.920, F10.921, F10.929, F10.930, F10.931, F10.932, F10.939, F10.94, F10.950, F10.951, F10.959, F10.96, F10.97, F10.980, F10.981, F10.982, F10.988, F10.99, G31.2, G62.1, I42.6, K29.20, K29.21, K70.0, K70.10, K70.11, K70.2, K70.30, K70.31, K70.40, K70.41, K70.9, O35.4XX0, O35.4XX1, O35.4XX2, O35.4XX3, O35.4XX4, O35.4XX5, O35.4XX9, O99.310, O99.311, O99.312, O99.313, O99.314, O99.315, Y90.0, Y90.1, Y90.2, Y90.3, Y90.4, Y90.5, Y90.6, Y90.7, Y90.8, Y90.9
Acute/Inpatient diagnoses	
Arrhythmia	I44.0, I44.1, I44.3, I44.30, I44.39, I44.4, I44.5, I44.6, I44.60, I44.69, I44.7, I45.0, I45.1, I45.2, I45.4, I45.5, I45.6, I45.7, I45.8, I45.9, I47, I47.0, I47.1, I47.2, I47.20, I47.21, I47.29, I47.9, I48, I48.0, I48.1, I48.11, I48.19, I48.2, I48.20, I48.21, I48.3, I48.4, I48.9, I48.9, I48.91, I48.92, I49, I49.0, I49.01, I49.02, I49.2, I49.3, I49.4, I49.40, I49.49, I49.5, I49.5, I49.8, I49.9, R00.0, R00.1, R00.8, T82.1, T82.11, T82.110, T82/110A, T82.110D, T82.110S, T82.111, T82.111A, T82.111D, T82.111S, T82.118, T82.118A, T82.118D, T82.118S, T82.119, T82.119A, T82.119D, T82.119S, T82.12, T82.120, T82.120A, T82.120D, T82.120S, T82.121, T82.121A, T82.121D, T82.121S, T82.128, T82.128A, T82.128D, T82.128S, T82.129, T82.129A, T82.129D, T82.129S, T82.19, T82.190, T82.190A, T82.190D, T82.190S, T82.191, T82.191A, T82.191D, T82.191S, T82.198, T82.198A, T82.198D, T82.198S, T82.199, T82.199A, T82.199D, T82.199S, Z45.0, Z95.0, Z95.810, Z95.818, Z95.9
Brain injury (trauma / hemorrhage / stroke)	S02.0XXA, S02.0XXB, S02.101A, S02.101B, S02.102A, S02.102B, S02.109A, S02.109B, S02.10XA, S02.10XB, S02.110A, S02.110B, S02.111A, S02.111B, S02.112A, S02.112B, S02.113A, S02.113B, S02.118A, S02.118B, S02.119A, S02.119B, S02.11AA, S02.11AB, S02.11BA, S02.11BB, S02.11CA, S02.11CB, S02.11DA, S02.11DB, S02.11EA, S02.11EB, S02.11FA, S02.11FB, S02.11GA, S02.11GB, S02.11HA, S02.11HB, S02.121A, S02.121B, S02.122A, S02.122B, S02.129A, S02.129B, S02.19XA, S02.19XB, S02.2XXA, S02.2XXB, S02.30XA, S02.30XB, S02.31XA, S02.31XB, S02.32XA, S02.32XB, S02.3XXA, S02.3XXB, S02.400A, S02.400B, S02.401A, S02.401B, S02.402A, S02.402B, S02.40AA, S02.40AB, S02.40BA, S02.40BB, S02.40CA, S02.40CB, S02.40DA, S02.40DB, S02.40EA, S02.40EB, S02.40FA, S02.40FB, S02.411A, S02.411B, S02.412A, S02.412B, S02.413A, S02.413B, S02.42XA, S02.42XB, S02.5XXA, S02.5XXB, S02.600A, S02.600B, S02.601A, S02.601B, S02.602A, S02.602B, S02.609A, S02.609B, S02.610A, S02.610B, S02.611A, S02.611B, S02.612A, S02.612B, S02.61XA, S02.61XB, S02.620A, S02.620B, S02.621A, S02.621B, S02.622A, S02.622B, S02.62XA, S02.62XB, S02.630A, S02.630B, S02.631A, S02.631B, S02.632A, S02.632B, S02.63XA, S02.63XB, S02.640A, S02.640B, S02.641A, S02.641B, S02.642A, S02.642B, S02.64XA, S02.64XB, S02.650A, S02.650B, S02.651A, S02.651B, S02.652A, S02.652B, S02.65XA, S02.65XB, S02.66XA, S02.66XB, S02.670A, S02.670B, S02.671A, S02.671B, S02.672A, S02.672B, S02.67XA, S02.67XB, S02.69XA, S02.69XB, S02.80XA, S02.80XB, S02.81XA, S02.81XB, S02.82XA, S02.82XB, S02.831A, S02.831B, S02.832A, S02.832B, S02.839A, S02.839B, S02.841A, S02.841B, S02.842A, S02.842B, S02.849A, S02.849B, S02.85XA, S02.85XB, S02.8XXA, S02.8XXB, S02.91XA, S02.91XB, S02.92XA, S02.92XB, S06.0X0A, S06.0X1A, S06.0X2A, S06.0X3A, S06.0X4A, S06.0X5A, S06.0X6A, S06.0X7A, S06.0X8A, S06.0X9A, S06.1X0A, S06.1X1A, S06.1X2A, S06.1X3A, S06.1X4A, S06.1X5A, S06.1X6A, S06.1X7A, S06.1X8A, S06.1X9A, S06.2X0A, S06.2X1A, S06.2X2A, S06.2X3A, S06.2X4A, S06.2X5A, S06.2X6A, S06.2X7A, S06.2X8A, S06.2X9A, S06.300A, S06.301A, S06.302A, S06.303A, S06.304A, S06.305A, S06.306A, S06.307A, S06.308A, S06.309A, S06.310A, S06.311A, S06.312A, S06.313A, S06.314A, S06.814A, S06.815A, S06.816A, S06.817A, S06.818A, S06.819A, S06.820A, S06.821A, S06.822A, S06.823A, S06.824A, S06.825A, S06.826A, S06.827A, S06.828A, S06.829A, S06.890A, S06.891A, S06.892A, S06.893A, S06.894A, S06.895A, S06.896A, S06.897A, S06.898A, S06.899A, S06.9X0A, S06.9X1A, S06.9X2A, S06.9X3A, S06.9X4A, S06.9X5A, S06.9X6A, S06.9X7A, S06.9X8A, S06.9X9A, R40.3, R40.20, R40.1, R40.0, K72.91, K72.11, K72.01, K71.11,

© 2025 Wolpaw BJ et al. *JAMA Network Open*.

	K70.41, G93.1, E15, E13.641, E13.11, E13.01, E11.641, E11.11, E11.01, R29.740, R29.739, R29.738, R29.737, R29.736, R29.735, R29.734, R29.733, R29.732, R29.731, R29.730, R29.729, R29.728, R29.727, R29.726, R29.725, R29.724, R29.723, R29.722, R29.721, R29.720, R29.719, R29.718, R29.717, R29.716, R29.715, R29.714, R29.713, R29.712, R29.711, R29.710, R29.709, R29.708, R29.707, R29.706, R29.705, R29.704, R29.703, R29.702, R29.701, R29.700, I97.821, I97.820, I97.811, I97.810, I63.9, I63.89, I63.81, I63.8, I63.6, I63.59, I63.549, I63.543, I63.542, I63.541, I63.539, I63.533, I63.532, I63.531, I63.529, I63.523, I63.522, I63.521, I63.519, I63.513, I63.512, I63.511, I63.50, I63.49, I63.449, I63.443, I63.442, I63.441, I63.439, I63.433, I63.432, I63.431, I63.429, I63.423, I63.422, I63.421, I63.419, I63.413, I63.412, I63.411, I63.40, I63.39, I63.349, I63.343, I63.342, I63.341, I63.339, I63.333, I63.332, I63.331, I63.329, I63.323, I63.322, I63.321, I63.319, I63.313, I63.312, I63.311, I63.30, I63.29, I63.239, I63.233, I63.232, I63.231, I63.22, I63.219, I63.213, I63.212, I63.211, I63.20, I63.19, I63.139, I63.133, I63.132, I63.131, I63.12, I63.119, I63.113, I63.112, I63.111, I63.10, I63.09, I63.039, I63.033, I63.032, I63.031, I63.02, I63.019, I63.013, I63.012, I63.011, I63.00, G43.619, G43.611, G43.609, G43.601, I62.9, I62.1, I62.03, I62.02, I62.01, I62.00, I61.9, I61.8, I61.6, I61.5, I61.4, I61.3, I61.2, I61.1, I61.0, I60.9, I60.8, I60.7, I60.6, I60.52, I60.51, I60.50, I60.4, I60.32, I60.31, I60.30, I60.22, I60.21, I60.20, I60.2, I60.12, I60.11, I60.10, I60.02, I60.01, I60.00, I69.298, I69.293, I69.292, I69.291, I69.290, I69.269, I69.265, I69.264, I69.263, I69.262, I69.261, I69.259, I69.254, I69.253, I69.252, I69.251, I69.249, I69.244, I69.243, I69.242, I69.241, I69.239, I69.234, I69.233, I69.232, I69.231, I69.228, I69.223, I69.222, I69.221, I69.220, I69.219, I69.218, I69.215, I69.214, I69.213, I69.212, I69.211, I69.210, I69.21, I69.20, I69.198, I69.193, I69.192, I68.8, I68.2, I68.0, I67.9, I67.89, I67.858, I67.850, I67.848, I67.841, I67.83, I67.82, I67.81, I67.7, I67.6, I67.5, I67.3, I67.2, I67.1, G46.8, G46.7, G46.6, G46.5, G46.4, G46.3, G46.2, G46.1, G46.0, A52.05, A52.04, I69.998, I69.993, I69.992, I69.991, I69.990, I69.969, I69.965, I69.964, I69.963, I69.962, I69.961, I69.959, I69.954, I69.953, I69.952, I69.951, I69.949, I69.944, I69.943, I69.942, I69.941, I69.939, I69.934, I69.933, I69.932, I69.931, I69.928, I69.923, I69.922, I69.921, I69.920, I69.919, I69.918, I69.915, I69.914, I69.913, I69.912, I69.911, I69.910, I69.91, I69.90, I69.898, I69.893, I69.892, I69.891, I69.890, I69.869, I69.865, I69.864, I69.863, I69.862, I69.861, I69.859, I69.854, I69.853, I69.852, I69.851, I69.849, I69.844, I69.843, I69.842, I69.841, I69.839, I69.834, I69.833, I69.832, I69.831, I69.828, I69.823, I69.822, I69.821, I69.820, I69.819, I69.818, I69.815, I69.814, I69.813, I69.812, I69.811, I69.810, I69.81, I69.80, I69.398, I69.393, I69.392, I69.391, I69.390, I69.369, I69.365, I69.364, I69.363, I69.362, I69.361, I69.359, I69.354, I69.353, I69.352, I69.351, I69.349, I69.344, I69.343, I69.342, I69.341, I69.339, I69.334, I69.333, I69.332, I69.331, I69.328, I69.323, I69.322, I69.321, I69.320, I69.319, I69.318, I69.315, I69.314, I69.313, I69.312, I69.311, I69.310, I69.31, I69.30
Gastrointestinal tract disorder	O87.2, O22.43, O22.42, O22.41, O22.40, K92.2, K92.1, K92.0, K91.32, K91.31, K91.30, K91.3, K83.2, K82.A2, K82.2, K75.0, K68.19, K68.12, K68.11, K67, K65.9, K65.8, K65.4, K65.3, K65.2, K65.1, K65.0, K64.9, K64.8, K64.5, K64.4, K64.3, K64.2, K64.1, K64.0, K63.1, K63.0, K62.9, K62.89, K62.82, K62.81, K62.7, K62.6, K62.5, K62.4, K62.3, K62.2, K62.1, K62.0, K61.5, K61.4, K61.39, K61.31, K61.3, K61.2, K61.1, K61.0, K60.5, K60.4, K60.3, K60.2, K60.1, K60.0, K59.4, K57.93, K57.92, K57.91, K57.90, K57.81, K57.80, K57.53, K57.52, K57.51, K57.50, K57.41, K57.40, K57.33, K57.32, K57.31, K57.30, K57.21, K57.20, K57.13, K57.12, K57.11, K57.10, K57.01, K57.00, K56.7, K56.699, K56.691, K56.690, K56.69, K56.609, K56.601, K56.600, K56.60, K56.52, K56.51, K56.50, K56.5, K56.49, K56.41, K56.3, K56.2, K56.1, K56.0, K55.21, K52.9, K52.89, K52.839, K52.838, K52.832, K52.831, K52.82, K52.81, K52.3, K52.29, K52.22, K52.21, K52.2, K52.1, K52.0, K51.919, K51.918, K51.914, K51.913, K51.912, K51.911, K51.90, K51.819, K51.818, K51.814, K51.813, K51.812, K51.811, K51.80, K51.519, K51.518, K51.514, K51.513, K51.512, K51.511, K51.50, K51.419, K51.418, K51.414, K51.413, K51.412, K51.411, K51.40, K51.319, K51.318, K51.314, K51.313, K51.312, K51.311, K51.30, K51.219, K51.218, K51.214, K51.213, K51.212, K51.211, K51.20, K51.019, K51.018, K51.014, K51.013, K51.012, K51.011, K51.00, K50.919, K50.918, K50.914, K50.913, K50.912, K50.911, K50.90, K50.819, K50.818, K50.814, K50.813, K50.812, K50.811, K50.80, K50.119, K50.118, K50.114, K50.113, K50.112, K50.111, K50.10, K50.019, K50.018, K50.014, K50.013, K50.012, K50.011, K50.00, K46.9, K46.1, K46.0, K45.8, K45.1, K45.0, K44.9, K44.1, K44.0, K43.9, K43.7, K43.6, K43.5, K43.4, K43.3, K26.1, K26.0, K25.9, K25.7, K25.6, K25.5, K25.4, K25.3, K25.2, K25.1, K25.0, K23, K22.9, K22.8, K22.719, K22.711, K22.710, K22.70, K22.6, K22.5, K22.4, K22.3, K22.2, K22.11, K22.10, K22.0, K21.9, K21.01, K21.00, K21.0, K20.91, K20.90, K20.9, K20.81, K20.80, K20.8, K20.0, I85.11, I85.01, B57.31, B37.81, A74.81, A63.0, A60.9, A60.1, A56.3, A54.85, A54.6, A51.1, A18.31
Liver disease	P35.3, O98.43, O98.42, O98.419, O98.413, O98.412, O98.411, K70.11, K70.10, B58.1, B26.81, B25.1, B19.9, B19.21, B19.20, B19.11, B19.10, B19.0, B18.9, B18.8, B18.2, B18.1, B18.0, B17.9, B17.8, B17.2, B17.11, B17.10, B17.0, B16.9, B16.2, B16.1, B16.0, B15.9, B15.0, B00.81, A51.45,

© 2025 Wolpaw BJ et al. *JAMA Network Open*.

	K91.82, K72.91, K72.90, K72.11, K72.10, K72.01, K72.00, K70.41, K70.40, O26.63, O26.62, O26.619, O26.613, O26.612, O26.611, K77, K76.9, K76.89, K76.81, K76.7, K76.6, K76.5, K76.4, K76.3, K76.2, K76.1, K76.0, K75.9, K75.89, K75.1, K74.69, K74.60, K74.5, K74.4, K74.3, K74.2, K74.1, K74.02, K74.01, K74.00, K74.0, K71.9, K71.8, K71.7, K71.11, K71.10, K71.0, K70.9, K70.31, K70.30, K70.2, K70.0, B67.5, B67.0, A52.74, K92.9, K92.89, K92.81, K90.9, K90.89, K90.81, K90.49, K90.41, K90.4, K90.2, K90.1, K90.0, K75.81, K75.4, K75.3, K75.2, K73.9, K73.8, K73.2, K73.1, K73.0, K71.6, K71.51, K71.50, K71.4, K71.3, K71.2, K68.9, K66.9, K66.8, K66.1, K66.0, K63.9, K63.89, K63.81, K63.4, K63.3, K63.2, K59.9, K59.89, K59.81, K59.8, K59.39, K59.31, K59.3, K59.2, K59.1, K59.09, K59.04, K59.03, K59.02, K59.01, K59.00, K58.9, K58.8, K58.2, K58.1, K58.0, K55.9, K55.8, K55.33, K55.32, K55.31, K55.30, K55.21, K55.20, K55.1, K55.069, K55.062, K55.061, K55.059, K55.052, K55.051, K55.049, K55.042, K55.041, K55.039, K55.032, K55.031, K55.029, K55.022, K55.021, K55.019, K55.012, K55.011, K55.0, B57.32, A18.83
Nutrition / electrolyte / acid-base disorder	O25.3, O25.2, O25.13, O25.12, O25.11, O25.10, E88.9, E88.89, E88.81, E88.49, E88.42, E88.41, E88.40, E88.3, E88.2, E88.1, E88.09, E88.02, E88.01, E87.8, E87.79, E87.71, E87.70, E87.6, E87.5, E87.4, E87.3, E87.2, E87.1, E87.0, E86.9, E86.1, E86.0, E85.9, E85.89, E85.82, E85.81, E85.8, E85.4, E85.3, E85.2, E85.1, E85.0, E83.9, E83.89, E83.81, E83.59, E83.52, E83.51, E83.50, E83.49, E83.42, E83.41, E83.40, E83.39, E83.32, E83.31, E83.30, E83.2, E83.19, E83.119, E83.118, E83.111, E83.110, E83.10, E83.09, E83.01, E83.00, E80.7, E80.6, E80.5, E80.4, E80.3, E80.29, E80.21, E80.20, E80.1, E80.0, E79.9, E79.8, E79.2, E79.1, E79.0, E78.9, E78.89, E78.81, E78.79, E78.72, E78.71, E78.70, E78.6, E77.9, E77.8, E77.1, E77.0, E76.9, E76.8, E76.3, E76.29, E76.22, E76.219, E76.211, E76.210, E76.1, E76.03, E76.02, E76.01, E75.6, E75.5, E75.4, E75.3, E75.29, E75.26, E75.25, E75.249, E75.248, E75.243, E75.242, E75.241, E75.240, E75.23, E75.22, E75.21, E75.19, E75.11, E75.10, E75.09, E75.02, E75.01, E75.00, E74.9, E74.89, E74.819, E74.818, E74.810, E74.8, E74.4, E74.39, E74.31, E74.29, E74.21, E74.20, E74.19, E74.12, E74.11, E74.10, E74.09, E74.04, E74.03, E74.02, E74.01, E74.00, E73.9, E73.8, E73.1, E73.0, E72.9, E72.89, E72.81, E72.8, E72.59, E72.53, E72.52, E72.51, E72.50, E72.4, E72.3, E72.29, E72.23, E72.22, E72.21, E72.20, E72.19, E72.12, E72.11, E72.10, E72.09, E72.04, E72.03, E72.02, E72.01, E72.00, E71.548, E71.542, E71.541, E71.540, E71.53, E71.529, E71.528, E71.522, E71.521, E71.520, E71.518, E71.511, E71.510, E71.50, E71.448, E71.440, E71.43, E71.42, E71.41, E71.40, E71.39, E71.32, E71.318, E71.314, E71.313, E71.312, E71.311, E71.310, E71.30, E71.2, E71.19, E71.128, E71.121, E22.2, E40, E41, E42, E43, E44, E44.0, E44.1, E45, E46, E64, E64.0, E64.1, E64.2, E64.3, E64.8, E64.9, E63.0, E63.1, E63.8, E63.9, R63.4, R64
Other substance use disorder (excluding alcohol)	T40.695A, T40.694A, T40.693A, T40.692A, T40.691A, T40.605A, T40.604A, T40.603A, T40.602A, T40.601A, T40.4X5A, T40.4X4A, T40.4X3A, T40.4X2A, T40.4X1A, T40.495A, T40.494A, T40.493A, T40.492A, T40.491A, T40.425A, T40.424A, T40.423A, T40.422A, T40.421D, T40.415A, T40.414A, T40.413A, T40.412A, T40.411A, T40.3X5A, T40.3X4A, T40.3X3A, T40.3X2A, T40.3X1A, T40.2X5A, T40.2X4A, T40.2X3A, T40.2X2A, T40.2X1A, T40.1X4A, T40.1X3A, T40.1X2A, T40.1X1A, T40.0X5A, T40.0X4A, T40.0X3A, T40.0X2A, T40.0X1A, F11.99, F11.988, F11.982, F11.981, F11.959, F11.951, F11.950, F11.94, F11.93, F11.929, F11.922, F11.921, F11.920, F11.90, F11.29, F11.288, F11.282, F11.281, F11.259, F11.251, F11.250, F11.24, F11.23, F11.229, F11.222, F11.221, F11.220, F11.20, F11.19, F11.188, F11.182, F11.181, F11.159, F11.151, F11.150, F11.14, F11.13, F11.129, F11.122, F11.121, F11.120, F11.10, T40.7X5A, T40.7X4A, T40.7X3A, T40.7X2A, T40.7X1A, F12.99, F12.988, F12.980, F12.959, F12.951, F12.950, F12.93, F12.929, F12.922, F12.921, F12.920, F12.90, F12.29, F12.288, F12.280, F12.259, F12.251, F12.250, F12.23, F12.229, F12.222, F12.221, F12.220, F12.20, F12.19, F12.188, F12.180, F12.159, F12.151, F12.150, F12.13, F12.129, F12.122, F12.121, F12.120, F12.10, T42.75XA, T42.74XA, T42.73XA, T42.72XA, T42.71XA, T42.6X5A, T42.6X4A, T42.6X3A, T42.6X2A, T42.6X1A, F13.99, F13.988, F13.982, F13.981, F13.980, F13.97, F13.96, F13.959, F13.951, F13.950, F13.94, F13.939, F13.932, F13.931, F13.930, F13.929, F13.921, F13.920, F13.90, F13.29, F13.288, F13.282, F13.281, F13.280, F13.27, F13.26, F13.259, F13.251, F13.250, F13.24, F13.239, F13.232, F13.231, F13.230, F13.229, F13.221, F13.220, F13.20, F13.19, F13.188, F13.182, F13.181, F13.180, F13.159, F13.151, F13.150, F13.14, F13.139, F13.132, F13.131, F13.130, F13.129, F13.121, F13.120, F13.10, T43.695A, T43.694A, T43.693A, T43.692A, T43.691A, T43.644A, T43.643A, T43.642A, T43.641A, T43.635A, T43.634A, T43.633A, T43.632A, T43.631A, T43.625A, T43.624A, T43.623A, T43.622A, T40.992A, T40.991A, T40.905A, T40.904A, T40.903A, T40.902A, T40.901A, T40.8X4A, T40.8X3A, T40.8X2A, T40.8X1A, F16.99, F16.988, F16.983, F16.980, F16.959, F16.951, F16.950, F16.94, F16.929, F16.921, F16.920, F16.90, F16.29, F16.288, F16.283, F16.280, F16.259, F16.251, F16.250, F16.24, F16.229, F16.221,

© 2025 Wolpaw BJ et al. *JAMA Network Open*.

	F16.220, F16.20, F16.19, F16.188, F16.183, F16.180, F16.159, F16.151, F16.150, F16.14, F16.129, F16.122, F16.121, F16.120, F16.10, T41.0X5A, T41.0X4A, T41.0X3A, T41.0X2A, T41.0X1A, F18.180, F18.17, F18.159, F18.151, F18.150, F18.14, F18.129, F18.121, F18.120, F18.10, O99.325, O99.324, O99.323, O99.322, O99.321, O99.320, O35.5XX9, O35.5XX5, O35.5XX4, O35.5XX3, O35.5XX2, O35.5XX1, O35.5XX0, F19.99, F19.988, F19.982, F19.981, F19.980, F19.97, F19.96, F19.959, F19.951, F19.950, F19.94, F19.939, F19.932, F19.931, F19.930, F19.929, F19.922, F19.921, F19.920, F19.90, F19.29, F19.288, F19.282, F19.281, F19.280, F19.27, F19.26, F19.259, F19.251, F19.250, F19.24, F19.239, F19.232, F19.231, F19.230, F19.229, F19.222, F19.221, F19.220, F19.20, F19.19, F19.188, F19.182, F19.181, F19.180, F19.17, F19.16, F19.159, F19.151, F19.150, F19.14, F19.139, F19.132, F19.131, F19.130, F19.129, F19.122, F19.121, F19.120, F19.10, T43.94XA, T43.93XA, T43.92XA, T43.91XA, T43.8X4A, T43.8X3A, T43.8X2A, T43.8X1A, T40.421A, T42.4X4A, T42.4X3A, T42.4X2A, T42.4X1A
Psychiatric condition	F55.0, F55.1, F55.2, F55.8, F55.3, F55.4, F40.241, F43.0, F43.22, F43.21, F43.24, F43.23, F43.25, F43.29, F43.20, F40.01, F40.02, F40.00, F10.180, F10.150, F10.151, F10.159, F10.280, F10.250, F10.251, F10.259, F10.980, F10.950, F10.951, F10.959, F40.290, F50.02, F50.01, F50.00, F60.2, F06.4, F41.9, F40.210, T71.232A, T71.222A, T71.132A, T71.162A, T71.192A, T71.122A, T71.152A, T71.112A, F60.6, F50.82, F50.81, F31.31, F31.30, F31.32, F31.5, F31.4, F31.0, F31.2, F31.11, F31.12, F31.13, F31.10, F31.61, F31.62, F31.64, F31.63, F31.60, F31.75, F31.71, F31.73, F31.77, F31.9, F31.81, F45.22, F60.3, F23, F50.2, F12.180, F12.150, F12.151, F12.159, F12.280, F12.250, F12.251, F12.259, F12.980, F12.950, F12.951, F12.959, F06.1, F20.2, F93.9, T61.02XA, F40.240, F14.180, F14.150, F14.151, F14.159, F14.280, F14.250, F14.251, F14.259, F14.980, F14.950, F14.951, F14.959, F91.0, F91.2, F91.1, F91.9, F44.7, F44.4, F44.5, F44.6, F34.0, F22, F60.7, F48.1, F94.2, F20.1, F34.81, F44.0, F44.9, F44.1, F44.81, F44.2, F64.1, F52.6, F34.1, F50.9, F42.4, F65.2, F68.A, F68.10, F68.13, F68.12, F68.11, F40.230, F40.242, F40.243, F40.231, F40.233, F40.232, F40.220, F52.31, F52.22, F65.0, F65.81, F64.2, F64.9, F41.1, F40.291, F16.180, F16.150, F16.151, F16.159, F16.280, F16.250, F16.251, F16.259, F16.980, F16.950, F16.951, F16.959, F60.4, F42.3, F52.0, F45.20, F45.21, F63.9, F18.180, F18.150, F18.151, F18.159, F18.280, F18.250, F18.251, F18.259, F18.980, F18.950, F18.951, F18.959, X82.0XXA, X82.1XXA, X82.2XXA, X74.01XA, X79.XXXA, X83.0XXA, X71.2XXA, X71.3XXA, X71.0XXA, X71.1XXA, X71.9XXA, X83.1XXA, X75.XXXA, X83.2XXA, X72.XXXA, X77.3XXA, X77.1XXA, X73.1XXA, X80.XXXA, X81.1XXA, X81.0XXA, X81.8XXA, X78.1XXA, X73.2XXA, X74.8XXA, X74.09XA, X77.2XXA, X77.8XXA, X73.8XXA, X78.8XXA, X83.8XXA, X74.02XA, X78.0XXA, X73.0XXA, X76.XXXA, F15.180, F15.150, F15.151, F15.159, F15.280, F15.250, F15.251, F15.259, F15.980, F15.950, F15.951, F15.959, F45.41, F45.42, F41.0, F60.0, F20.0, F65.9, F63.0, F65.4, F34.9, F07.0, F60.9, F40.9, F98.3, T39.1X2A, T44.6X2A, T36.5X2A, T43.622A, T50.7X2A, T38.7X2A, T46.4X2A, T38.812A, T37.4X2A, T45.0X2A, T48.6X2A, T44.0X2A, T45.7X2A, T45.512A, T47.6X2A, T50.6X2A, T36.7X2A, T38.6X2A, T46.6X2A, T37.2X2A, T37.1X2A, T45.1X2A, T42.8X2A, T49.1X2A, T39.4X2A, T45.522A, T38.2X2A, T48.3X2A, T45.3X2A, T48.4X2A, T40.412A, T44.2X2A, T38.0X2A, T45.622A, T40.1X2A, T47.0X2A, T42.0X2A, T42.1X2A, T50.Z12A, T41.0X2A, T38.3X2A, T41.1X2A, T45.4X2A, T49.4X2A, T41.3X2A, T49.0X2A, T49.2X2A, T50.1X2A, T40.8X2A, T36.3X2A, T40.3X2A, T43.632A, T50.0X2A, T42.5X2A, T50.A22A, T43.1X2A, T50.912A, T49.5X2A, T40.0X2A, T38.4X2A, T48.992A, T47.8X2A, T46.992A, T47.1X2A, T48.5X2A, T43.292A, T46.2X2A, T42.6X2A, T46.5X2A, T37.3X2A, T43.592A, T50.A92A, T44.992A, T48.292A, T50.992A, T38.5X2A, T45.692A, T41.292A, T38.992A, T38.892A, T47.4X2A, T40.692A, T39.8X2A, T39.392A, T40.2X2A, T44.3X2A, T44.1X2A, T45.8X2A, T40.992A, T43.692A, T43.8X2A, T37.8X2A, T40.4X2A, T40.492A, T36.8X2A, T49.8X2A, T50.Z92A, T50.B92A, T49.6X2A, T48.0X2A, T36.0X2A, T46.7X2A, T50.A12A, T43.3X2A, T44.4X2A, T44.5X2A, T39.312A, T39.2X2A, T36.6X2A, T39.092A, T47.3X2A, T43.212A, T43.222A, T48.1X2A, T50.B12A, T47.2X2A, T42.2X2A, T37.0X2A, T43.022A, T36.4X2A, T41.5X2A, T45.612A, T38.1X2A, T40.422A, T43.012A, T48.902A, T46.902A, T47.92XA, T41.42XA, T43.202A, T42.72XA, T43.502A, T48.202A, T44.902A, T50.902A, T45.602A, T41.202A, T38.902A, T38.802A, T40.602A, T39.92XA, T45.92XA, T40.902A, T43.602A, T43.92XA, T37.92XA, T36.92XA, T49.92XA, T45.2X2A, F43.11, F43.12, F43.10, F53.0, O90.6, F52.4, F32.81, F54, F06.2, F06.0, F53.1, F53, F63.1, F43.9, F94.1, F20.5, F98.21, F65.50, F25.0, F25.1, F25.9, F60.1, F20.9, F20.81, F21, T61.12XA, F13.180, F13.150, F13.151, F13.159, F13.280, F13.250, F13.251, F13.259, F13.980, F13.950, F13.951, F13.959, F94.0, F93.0, F52.1, R37, F65.51, F65.52, F24, F40.11, F40.10, F45.0, F45.9, R45.851, T14.91, T14.91XA, T51.2X2A, T64.02XA, T57.0X2A, T52.1X2A, T56.7X2A, T56.3X2A, T59.7X2A, T65.4X2A, T58.2X2A, T58.02XA, T58.8X2A, T58.92XA, T58.12XA, T53.0X2A, T65.212A, T59.4X2A, T53.5X2A, T53.1X2A, T56.2X2A,

© 2025 Wolpaw BJ et al. *JAMA Network Open*.

	T63.042A, T63.622A, T63.832A, T63.892A, T63.592A, T63.692A, T63.792A, T63.612A, T63.632A, T63.512A, T63.92XA, T63.812A, T63.712A, T63.822A, T56.4X2A, T63.022A, T54.2X2A, T54.3X2A, T65.0X2A, T55.1X2A, T53.4X2A, T51.0X2A, T65.832A, T59.5X2A, T59.2X2A, T51.3X2A, T52.3X2A, T60.1X2A, T65.822A, T63.322A, T63.462A, T56.5X2A, F64.0, F65.1, F63.3, F20.3, F45.1, F98.9, F59, F69, F09, F39, F07.9, F29, F52.9, F52.5, F65.3, Z04.6, Z13.30, Z13.39, Z13.31, Z13.32
Seizure	G40.A11, G40.A19, G40.A01, G40.A09, R56.01, G40.42, G40.833, G40.834, G40.911, G40.919, G40.901, G40.909, G40.501, G40.509, G40.823, G40.824, G40.821, G40.822, G40.311, G40.319, G40.301, G40.309, G40.B11, G40.B19, G40.B01, G40.B09, G40.813, G40.814, G40.811, G40.812, G40.011, G40.019, G40.001, G40.009, G40.211, G40.219, G40.201, G40.209, G40.111, G40.119, G40.101, G40.109, G40.803, G40.804, G40.801, G40.802, G40.411, G40.419, G40.401, G40.409, G40.89, R56.1, R56.00, R56.9
Sepsis / shock / infection	O86.29, O86.22, O86.21, O86.20, O23.43, O23.42, O23.41, O23.40, O23.33, O23.32, O23.31, O23.30, O23.23, O23.22, O23.21, O23.20, O23.13, O23.12, O23.11, O23.10, O23.03, O23.02, O23.01, O23.00, N39.0, N34.3, N34.2, N34.1, N34.0, N30.91, N30.90, N30.81, N30.80, N30.41, N30.40, N30.31, N30.30, N30.21, N30.20, N30.11, N30.10, N30.01, N30.00, N15.1, N13.6, N12, N11.9, N11.8, N11.1, N11.0, N10, T81.44XA, R65.20, P37.9, P37.8, P37.5, P37.4, P37.3, P37.2, P37.1, P37.0, P35.9, P35.8, P35.4, P35.2, P35.1, P35.0, P23.8, P23.6, P23.5, P23.4, P23.3, P23.2, P23.1, P23.0, O98.73, O98.72, O98.719, O98.713, O98.712, O98.711, O98.63, O98.62, O98.619, O98.613, O98.612, O98.611, O98.33, O98.32, O98.319, O98.313, O98.312, O98.311, O98.23, O98.22, O98.219, O98.213, O98.212, O98.211, O98.13, O98.12, O98.119, O98.113, O98.112, O98.111, O98.03, O98.02, O98.019, O98.013, O98.012, O98.011, O86.04, O85, O26.43, O26.42, O26.41, O26.40, O08.82, O07.37, O04.87, O03.87, O03.37, K65.2, J65, I76, I02.9, I00, B99.9, B99.8, B97.89, B97.81, B97.7, B97.6, B97.5, B97.4, B97.39, B97.35, B97.34, B97.33, B97.32, B97.31, B97.30, B97.29, B97.21, B97.19, B97.12, B97.11, B97.10, B97.0, B96.89, B96.82, B96.81, B96.7, B96.6, B96.5, B96.4, B96.3, B96.29, B96.23, B96.22, B96.21, B96.20, B96.1, B96.0, B95.8, B95.7, B95.62, B95.61, B95.5, B95.4, B95.3, B95.2, B95.1, B95.0, B89, B88.9, B88.8, B88.3, B88.2, B88.1, B88.0, B87.9, B87.89, B87.82, B87.81, B87.4, B87.3, B87.2, B87.1, B87.0, B86, B85.4, B85.3, B85.2, B85.1, B85.0, B83.9, B83.8, B83.4, B83.3, B83.2, B83.1, B83.0, B82.9, B82.0, B81.8, B81.4, B81.3, B81.2, B81.1, B81.0, B80, B79, B78.9, B78.7, B52.8, B52.0, B51.9, B51.8, B51.0, B50.9, B50.8, B50.0, B49, B48.8, B48.4, B48.3, B48.2, B48.1, B48.0, B47.9, B47.1, B47.0, B46.9, B46.8, B46.5, B46.4, B46.3, B46.2, B46.1, B46.0, B45.9, B45.8, B45.7, B45.3, B45.2, B45.0, B44.9, B44.89, B44.81, B44.7, B44.2, B44.1, B44.0, B43.9, B43.8, B43.2, B43.1, B43.0, B42.9, B42.89, B42.82, B42.7, B42.1, B42.0, B41.9, B41.8, B41.7, B37.41, B37.3, B37.2, B37.0, B36.9, B36.8, B36.3, B36.2, B36.1, B36.0, B35.9, B35.8, B35.6, B35.5, B35.4, B35.3, B35.2, B35.1, B35.0, B34.9, B34.8, B34.4, B34.3, B34.2, B34.1, B34.0, B33.8, B33.4, B33.3, B33.24, B33.23, B33.22, B33.21, B33.20, B33.1, B33.0, B30.9, B30.8, B30.3, B30.2, B30.1, B30.0, B27.99, B27.91, B27.90, B27.89, B27.81, B27.80, B27.19, B27.11, B27.10, B27.09, B27.01, B27.00, B26.9, B26.89, B26.85, B26.84, B26.83, B26.82, B26.81, B26.3, B26.0, B25.9, B25.8, B25.2, B25.1, B20, B19.9, B19.21, B19.20, B19.11, B19.10, B19.0, B18.9, B18.8, B18.2, B18.1, B18.0, B17.9, B17.8, B17.2, B17.11, B17.10, B17.0, B16.9, B16.2, B16.1, B16.0, B15.9, B15.0, B10.89, B10.82, B10.81, B09, B08.8, B08.79, B08.72, B08.71, B08.70, B08.69, B08.62, B08.61, B08.60, B08.4, B08.3, B08.22, B08.21, B08.20, B08.1, B08.09, B08.04, B08.03, B08.02, B08.011, B08.010, B07.9, B07.8, B07.0, B06.9, B06.89, B06.82, B06.09, B06.00, B05.9, B05.89, B05.81, B05.4, B05.3, B04, B03, B02.9, B02.8, B02.7, B02.39, B02.34, B02.33, B02.32, B02.31, B02.30, B02.29, B02.24, B02.23, B02.22, B02.21, B01.9, B01.89, B01.81, B01.12, B00.9, B00.89, B00.82, B00.81, B00.59, B00.53, B00.52, B00.51, B00.50, B00.1, B00.0, A99, A98.8, A98.5, A98.4, A98.3, A98.2, A98.1, A98.0, A96.9, A96.8, A96.2, A96.1, A96.0, A95.9, A95.1, A95.0, A94, A93.8, A93.2, A93.1, A93.0, A92.9, A92.8, A92.5, A92.4, A92.39, A92.32, A92.30, A92.1, A92.0, A91, A90, A79.9, A79.89, A79.81, A79.1, A79.0, A78, A77.9, A77.8, A77.49, A77.41, A77.40, A77.3, A77.2, A77.1, A77.0, A75.9, A75.3, A75.2, A75.1, A75.0, A74.9, A74.89, A74.81, A74.0, A71.9, A71.1, A71.0, A70, A69.9, A52.15, A52.09, A52.06, A52.05, A52.04, A52.03, A52.02, A52.01, A52.00, A51.9, A51.5, A51.49, A51.46, A51.45, A51.44, A51.43, A51.42, A51.32, A51.31, A51.2, A51.1, A51.0, A50.9, A50.7, A50.6, A50.59, A50.57, A50.56, A50.55, A50.54, A50.53, A50.52, A50.51, A50.49, A50.45, A50.44, A50.43, A50.40, A50.39, A50.32, A50.31, A50.30, A50.2, A50.1, A50.09, A50.08, A50.07, A50.06, A50.02, A50.01, A49.9, A49.8, A49.3, A49.2, A49.1, A49.02, A49.01, A48.8, A48.52, A48.4, A48.3, A48.2, A48.0, A44.9, A44.8, A44.1, A44.0, A43.9, A43.8, A42.9, A42.89, A42.2, A42.1, A42.0, A39.9, A39.89, A39.84, A39.83, A39.82, A39.53, A39.52, A39.51, A39.50, A39.1, A38.9, A38.8, A38.1, A38.0, A37.90, A37.80, A37.10, A37.00, A36.9, A36.89, A36.86, A36.85, A36.84, A36.83, A36.81, A35, A34, A33, A32.9, A32.89, A32.82, A32.81, A32.0, A31.9, A31.8, A31.2, A30.9, A30.8, A30.5, A30.4,

© 2025 Wolpaw BJ et al. *JAMA Network Open*.

	A30.3, A30.2, A30.1, A30.0, A28.9, A28.8, A28.2, A28.1, A28.0, A27.9, A27.89, A27.0, A26.9, A26.8, A26.0, A25.9, A25.1, A25.0, A24.9, A24.3, A24.2, A24.1, A24.0, A23.9, A23.8, A23.3, A23.2, A23.1, A23.0, A22.9, A22.8, A22.7, A22.2, A22.0, A21.9, A21.8, A21.7, A21.3, A21.1, A21.0, A20.9, A20.8, A20.7, A20.1, A20.0, A19.9, A19.8, A19.2, A19.1, A19.0, A18.89, A18.85, A18.84, A18.83, A18.82, A18.81, A18.7, A18.6, A18.59, A18.54, A18.53, A18.52, A18.51, A18.50, A18.4, A18.39, A18.32, A18.31, A18.2, A18.18, A18.17, A18.16, A18.15, A18.14, A18.13, A18.12, A18.11, A18.10, A18.09, A18.03, A18.02, A18.01, A17.9, A17.89, A17.83, A17.81, A15.9, A15.8, A15.7, A15.6, A15.4, A15.0, A09, A08.8, A08.4, A08.39, A08.32, A08.31, A08.2, A08.19, A08.11, A08.0, A07.9, A07.8, A07.4, A07.3, A07.2, A07.1, A07.0, A06.9, A06.89, A06.82, A06.81, A06.7, A06.6, A06.5, A06.4, A06.3, A06.2, A06.1, A06.0, A04.9, A04.8, A04.72, A04.71, A04.7, A04.6, A04.5, A04.4, A04.3, A04.2, A04.1, A04.0, A03.9, A03.8, A03.3, A03.2, A03.1, A03.0, A02.9, A02.8, A02.29, A02.25, A02.24, A02.23, A02.20, A02.1, A02.0, A01.4, A01.3, A01.2, A01.1, A01.09, A01.05, A01.04, A01.02, A01.00, A00.9, A00.1, A00.0, G14, G05.4, G05.3, G04.91, G04.90, G04.89, G04.81, G04.39, G04.32, G04.31, G04.30, G04.2, G04.02, G04.01, G04.00, G03.9, G03.8, G03.2, G03.1, G03.0, G02, G01, G00.9, G00.8, G00.3, G00.2, G00.1, G00.0, D86.81, B60.11, B58.2, B57.42, B57.41, B45.1, B42.81, B40.81, B38.4, B37.5, B27.92, B27.82, B27.12, B27.02, B26.2, B26.1, B10.09, B10.01, B06.02, B06.01, B05.1, B05.0, B02.1, B02.0, B01.11, B01.0, B00.4, B00.3, A92.31, A92.2, A89, A88.8, A88.1, A88.0, A87.9, A87.8, A87.2, A87.1, A87.0, A86, A85.8, A85.2, A85.1, A85.0, A84.9, A84.89, A84.81, A84.8, A84.1, A84.0, A83.9, A83.8, A83.6, A83.5, A83.4, A83.3, A83.2, A83.1, A83.0, A82.9, A82.1, A82.0, A81.9, A81.89, A81.83, A81.82, A81.81, A81.2, A81.1, A81.09, A81.01, A81.00, A80.9, A80.4, A80.39, A80.30, A80.2, A80.1, A80.0, A69.21, A54.81, A52.3, A52.2, A52.19, A52.17, A52.14, A52.13, A52.12, A52.11, A52.10, A51.41, A50.42, A50.41, A42.82, A42.81, A39.81, A39.0, A32.12, A32.11, A27.81, A20.3, A17.82, A17.1, A17.0, A02.21, A01.01, J95.851, J85.1, J40, J39.9, J39.8, J39.3, J39.2, J39.1, J39.0, J38.7, J38.6, J38.5, J38.4, J38.3, J38.2, J38.1, J38.02, J38.01, J38.00, J37.1, J37.0, J36, J35.9, J35.8, J35.3, J35.2, J35.1, J35.03, J35.02, J35.01, J34.9, J34.89, J34.81, J34.3, J34.2, J34.1, J34.0, J33.9, J33.8, J33.1, J33.0, J32.9, J32.8, J32.4, J32.3, J32.2, J32.1, J32.0, J31.2, J30.81, J30.5, J30.2, J30.1, J30.0, J21.9, J21.8, J21.1, J21.0, J20.9, J20.8, J20.7, J20.6, J20.5, J20.4, J20.3, J20.2, J20.1, J20.0, J18.9, J18.8, J18.1, J18.0, J17, J16.8, J16.0, J15.9, J15.8, J15.7, J15.6, J15.5, J15.4, J15.3, J15.29, J15.212, J15.211, J15.20, J15.1, J15.0, J14, J13, J12.9, J12.89, J12.82, J12.81, J12.3, J12.2, J12.1, J12.0, J11.89, J11.83, J11.82, J11.81, J11.2, J11.1, J11.08, J11.00, J10.89, J10.83, J10.82, J10.81, J10.2, J10.1, J10.08, J10.01, J10.00, J09.X9, J09.X3, J09.X2, J09.X1, J06.9, J06.0, J05.11, J05.10, J05.0, J04.31, J04.30, J04.2, J04.11, J04.10, J04.0, J03.91, J03.90, J03.81, J03.80, J03.01, J03.00, J02.9, J02.8, J02.0, J01.91, J01.90, J01.81, J01.80, J01.41, J01.40, J01.31, J01.30, J01.21, J01.20, J01.11, J01.10, J01.01, J01.00, J00, B77.81, B59, B58.3, B39.2, B39.1, B39.0, B38.2, B38.1, B38.0, B37.1, B25.0, B08.5, B06.81, B05.2, B01.2, B00.2, A56.4, A54.84, A54.5, A52.73, A52.72, A50.05, A50.04, A50.03, A48.1, A43.0, A37.91, A37.81, A37.11, A37.01, A36.2, A36.1, A36.0, A31.0, A22.1, A21.2, A20.2, A15.5, A02.22, A01.03, T88.2XXA, T81.19XA, T81.12XA, T81.11XA, T81.10XA, R65.21, R57.9, R57.8, R57.1, R57.0, O75.1, O08.3, O07.31, O04.81, O03.81, O03.31, O26.53, O26.52, O26.51, O26.50, I95.9, I95.89, I95.81, I95.3, I95.2, I95.1, I95.0, M86.9, M86.8X9, M86.8X8, M86.8X7, M86.8X6, M86.8X5, M86.8X4, M86.8X3, M86.8X2, M86.8X1, M86.8X0, M86.69, M86.68, M86.679, M86.672, M86.671, M86.669, M86.662, M86.661, M86.659, M86.652, M86.651, M86.649, M86.642, M86.641, M86.639, M86.632, M86.631, M86.629, M86.622, M86.621, M86.619, M86.612, M86.611, M86.60, M86.59, M86.58, M86.579, M86.572, M86.571, M86.569, M86.562, M86.561, M86.559, M86.552, M86.551, M86.549, M86.542, M86.541, M86.539, M86.532, M86.531, M86.529, M86.522, M86.521, M86.519, M86.512, M86.511, M86.50, M86.49, M86.48, M86.479, M86.472, M86.471, M86.469, M86.462, M86.461, M86.459, M86.452, M86.451, M86.449, M86.442, M86.441, M86.439, M86.432, M86.431, M86.429, M86.422, M86.421, M86.419, M86.412, M86.411, M86.40, M86.39, M86.38, M86.379, M86.372, M86.371, M86.369, M86.362, M86.361, M86.359, M86.352, M86.351, M86.349, M86.342, M86.341, M86.339, M86.332, M86.331, M86.329, M86.322, M86.321, M86.319, M86.312, M86.311, M86.30, M86.29, M86.28, M86.279, M86.272, M86.271, M86.269, M86.262, M86.261, M86.259, M86.252, M86.251, M86.249, M86.242, M86.241, M86.239, M86.232, M86.231, M86.229, M86.222, M86.221, M86.219, M86.212, M86.211, M86.20, M86.19, M86.18, M86.179, M86.172, M86.171, M86.169, M86.162, M86.161, M86.159, M86.152, M86.151, M86.149, M86.142, M86.141, M86.139, M86.132, M86.131, M86.129, M86.122, M86.121, M86.119, M86.112, M86.111, M86.10, M86.09, M86.08, M86.079, M86.072, M86.071, M86.069, M86.062, M86.061, M86.059, M86.052, M86.051, M86.049, M86.042, M86.041, M86.039, M86.032, M86.031, M86.029, M86.022, M86.021, M86.019, M86.012, M86.011, M86.00, M71.09, M71.08, M71.079, M71.072, M71.071, M71.069, M71.062, M71.061, M71.059, M71.052, M71.051, M71.049, M71.042, M71.041, M71.039, M71.032, M71.031, M71.029, M71.022, M71.021, M71.019, M71.012, M71.011, M71.00, M65.08, M65.079, M65.072, M65.071, M65.069,

© 2025 Wolpaw BJ et al. *JAMA Network Open*.

	M65.062, M65.061, M65.059, M65.052, M65.051, M65.049, M65.042, M65.041, M65.039, M65.032, M65.031, M65.029, M65.022, M65.021, M65.019, M65.012, M65.011, M65.00, M46.28, M46.27, M46.26, M46.25, M46.24, M46.23, M46.22, M46.21, M46.20, M01.X9, M01.X8, M01.X79, M01.X72, M01.X71, M01.X69, M01.X62, M01.X61, M01.X59, M01.X52, M01.X51, M01.X49, M01.X42, M01.X41, M01.X39, M01.X32, M01.X31, M01.X29, M01.X22, M01.X21, M01.X19, M01.X12, M01.X11, M01.X0, M00.9, M00.89, M00.88, M00.879, M00.872, M00.871, M00.869, M00.862, M00.861, M00.859, M00.852, M00.851, M00.849, M00.842, M00.841, M00.839, M00.832, M00.831, M00.829, M00.822, M00.821, M00.819, M00.812, M00.811, M00.80, M00.29, M00.28, M00.279, M00.272, M00.271, M00.269, M00.262, M00.261, M00.259, M00.252, M00.251, M00.249, M00.242, M00.241, M00.239, M00.232, M00.231, M00.229, M00.222, M00.221, M00.219, M00.212, M00.211, M00.20, M00.19, M00.18, M00.179, M00.172, M00.171, M00.169, M00.162, M00.161, M00.159, M00.152, M00.151, M00.149, M00.142, M00.141, M00.139, M00.132, M00.131, M00.129, M00.122, M00.121, M00.119, M00.112, M00.111, M00.10, M00.09, M00.08, M00.079, M00.072, M00.071, M00.069, M00.062, M00.061, M00.059, M00.052, M00.051, M00.049, M00.042, M00.041, M00.039, M00.032, M00.031, M00.029, M00.022, M00.021, M00.019, M00.012, M00.011, M00.00, H05.029, H05.023, H05.022, H05.021, L30.3, L08.9, L08.89, L08.82, L08.81, L08.1, L08.0, L05.92, L05.91, L05.02, L05.01, L04.9, L04.8, L04.3, L04.2, L04.1, L04.0, L03.91, L03.90, L03.898, L03.891, L03.818, L03.811, L03.329, L03.327, L03.326, L03.325, L03.324, L03.323, L03.322, L03.321, L03.319, L03.317, L03.316, L03.315, L03.314, L03.313, L03.312, L03.311, L03.222, L03.221, L03.213, L03.212, L03.211, L03.129, L03.126, L03.125, L03.124, L03.123, L03.122, L03.121, L03.119, L03.116, L03.115, L03.114, L03.113, L03.112, L03.111, L03.049, L03.042, L03.041, L03.039, L03.032, L03.031, L03.029, L03.022, L03.021, L03.019, L03.012, L03.011, L02.93, L02.92, L02.91, L02.838, L02.831, L02.828, L02.821, L02.818, L02.811, L02.639, L02.632, L02.631, L02.629, L02.622, L02.621, L02.619, L02.612, L02.611, L02.539, L02.532, L02.531, L02.529, L02.522, L02.521, L02.519, L02.512, L02.511, L02.439, L02.436, L02.435, L02.434, L02.433, L02.432, L02.431, L02.429, L02.426, L02.425, L02.424, L02.423, L02.422, L02.421, L02.419, L02.416, L02.415, L02.414, L02.413, L02.412, L02.411, L02.33, L02.32, L02.31, L02.239, L02.236, L02.235, L02.234, L02.233, L02.232, L02.231, L02.229, L02.226, L02.225, L02.224, L02.223, L02.222, L02.221, L02.219, L02.216, L02.215, L02.214, L02.213, L02.212, L02.211, L02.13, L02.12, L02.11, L02.03, L02.02, L02.01, L01.1, L01.09, L01.03, L01.02, L01.01, L01.00, L00, H05.019, H05.013, H05.012, H05.011, A51.39, A46, A43.1, A36.3, A31.1
Trauma	V90.00XA, V90.01XA, V90.02XA, V90.03XA, V90.04XA, V90.05XA, V90.06XA, V90.08XA, V90.09XA, V90.10XA, V90.11XA, V90.12XA, V90.13XA, V90.14XA, V90.15XA, V90.16XA, V90.18XA, V90.19XA, V90.20XA, V90.21XA, V90.22XA, V90.23XA, V90.24XA, V90.25XA, V90.26XA, V90.27XA, V90.28XA, V90.29XA, V90.30XA, V90.31XA, V90.32XA, V90.33XA, V90.34XA, V90.35XA, V90.36XA, V90.37XA, V90.38XA, V90.39XA, V90.80XA, V90.81XA, V90.82XA, V90.83XA, V90.84XA, V90.85XA, V90.86XA, V90.87XA, V90.88XA, V90.89XA, V92.00XA, V92.01XA, V92.02XA, V92.03XA, V92.04XA, V92.05XA, V92.06XA, V92.07XA, V92.08XA, V92.09XA, V92.10XA, V92.11XA, V92.12XA, V92.13XA, V92.14XA, V92.15XA, V92.16XA, V92.19XA, V92.20XA, V92.21XA, V92.22XA, V92.23XA, V92.24XA, V92.25XA, V92.26XA, V92.27XA, V92.28XA, V92.29XA, W16.011A, W16.021A, W16.031A, W16.111A, W16.121A, W16.131A, W16.211A, W16.221A, W16.311A, W16.321A, W16.331A, W16.41XA, W16.511A, W16.521A, W16.531A, W16.611A, W16.621A, W16.711A, W16.721A, W16.811A, W16.821A, W16.831A, W16.91XA, W22.041A, W65.XXXA, W67.XXXA, W69.XXXA, W73.XXXA, W74.XXXA, X71.0XXA, X71.1XXA, X71.2XXA, X71.3XXA, X71.8XXA, X71.9XXA, X92.0XXA, X92.1XXA, X92.2XXA, X92.3XXA, X92.8XXA, X92.9XXA, Y21.0XXA, Y21.1XXA, Y21.2XXA, Y21.3XXA, Y21.4XXA, Y21.8XXA, Y21.9XXA, V00.111A, V00.121A, V00.131A, V00.141A, V00.151A, V00.181A, V00.211A, V00.221A, V00.281A, V00.311A, V00.321A, V00.381A, V00.811A, V00.821A, V00.831A, V00.841A, V00.891A, W00.0XXA, W00.1XXA, W00.2XXA, W00.9XXA, W01.0XXA, W01.10XA, W01.110A, W01.111A, W01.118A, W01.119A, W01.190A, W01.198A, W03.XXXA, W04.XXXA, W05.0XXA, W05.1XXA, W05.2XXA, W06.XXXA, W07.XXXA, W08.XXXA, W09.0XXA, W09.1XXA, W09.2XXA, W09.8XXA, W10.0XXA, W10.1XXA, W10.2XXA, W10.8XXA, W10.9XXA, W11.XXXA, W12.XXXA, W13.0XXA, W13.1XXA, W13.2XXA, W13.3XXA, W13.4XXA, W13.8XXA, W13.9XXA, W14.XXXA, W15.XXXA, W16.012A, W16.022A, W16.032A, W16.112A, W16.122A, W16.132A, W16.212A, W16.222A, W16.312A, W16.322A, W16.332A, W16.42XA, W16.512A, W16.522A, W16.532A, W16.612A, W16.622A, W16.712A, W16.722A, W16.812A, W16.822A, W16.832A, W16.92XA, W17.0XXA, W17.1XXA, W17.2XXA, W17.3XXA, W17.4XXA, W17.81XA, W17.82XA, W17.89XA, W18.11XA, W18.12XA, W18.2XXA, Y36.391A, Y37.300A, Y37.301A, Y37.330A, Y37.331A, Y37.390A, Y37.391A, Y38.3X1A, Y38.3X2A, Y38.3X3A, W32.0XXA, W32.1XXA, W33.00XA, W33.01XA, W33.02XA, W33.03XA, W33.09XA,

© 2025 Wolpaw BJ et al. *JAMA Network Open*.

W33.10XA, W33.11XA, W33.12XA, W33.13XA, W33.19XA, W34.00XA, W34.09XA, W34.10XA, W34.19XA, X72.XXXA, X73.0XXA, X73.1XXA, X73.2XXA, X73.8XXA, X73.9XXA, X74.8XXA, X74.9XXA, X93.XXXA, X94.0XXA, X94.1XXA, X94.2XXA, X94.8XXA, X94.9XXA, X95.8XXA, X95.9XXA, Y22.XXXA, Y23.0XXA, Y23.1XXA, Y23.2XXA, Y23.3XXA, Y23.8XXA, Y23.9XXA, Y24.8XXA, Y24.9XXA, Y35.001A, Y35.002A, Y37.431A, Y37.92XA, Y38.4X1A, Y38.4X2A, Y38.4X3A, W24.0XXA, W24.1XXA, W30.0XXA, W30.1XXA, W30.2XXA, W30.3XXA, W30.81XA, W30.89XA, W30.9XXA, W31.0XXA, W31.1XXA, W31.2XXA, W31.3XXA, W31.81XA, W31.82XA, W31.83XA, W31.89XA, W31.9XXA, V02.10XA, V02.11XA, V02.12XA, V02.131A, V02.138A, V02.19XA, V02.90XA, V02.91XA, V02.92XA, V02.931A, V02.938A, V02.99XA, V03.10XA, V03.11XA, V03.12XA, V03.131A, V03.138A, V03.19XA, V03.90XA, V03.91XA, V03.92XA, V03.931A, V03.938A, V03.99XA, V04.10XA, V04.11XA, V04.12XA, V04.131A, V04.138A, V04.19XA, V04.90XA, V04.91XA, V04.92XA, V04.931A, V04.938A, V04.99XA, V09.20XA, V09.21XA, V09.29XA, V09.3XXA, V12.3XXA, V12.4XXA, V12.5XXA, V12.9XXA, V13.3XXA, V13.4XXA, V13.5XXA, V13.9XXA, V14.3XXA, V14.4XXA, V14.5XXA, V14.9XXA, V19.40XA, V19.49XA, V19.50XA, V19.59XA, V19.60XA, V19.69XA, V19.9XXA, V20.3XXA, V20.4XXA, V20.5XXA, V20.9XXA, V21.3XXA, V21.4XXA, V21.5XXA, V21.9XXA, V22.3XXA, V22.4XXA, V22.5XXA, V22.9XXA, V23.3XXA, V23.4XXA, V23.5XXA, V23.9XXA, V24.3XXA, V24.4XXA, V24.5XXA, V24.9XXA, V25.3XXA, V25.4XXA, V25.5XXA, V25.9XXA, V26.3XXA, V26.4XXA, V26.5XXA, V26.9XXA, V27.3XXA, V27.4XXA, V27.5XXA, V27.9XXA, V28.3XXA, V28.4XXA, V28.5XXA, V28.9XXA, V29.40XA, V29.49XA, V29.50XA, V29.59XA, V29.60XA, V29.69XA, V29.81XA, V29.88XA, V29.9XXA, V30.4XXA, V30.5XXA, V30.6XXA, V30.7XXA, V30.9XXA, V31.4XXA, V31.5XXA, V31.6XXA, V31.7XXA, V31.9XXA, V32.4XXA, V32.5XXA, V32.6XXA, V32.7XXA, V32.9XXA, V33.4XXA, V33.5XXA, V33.6XXA, V33.7XXA, V33.9XXA, V34.4XXA, V34.5XXA, V34.6XXA, V34.7XXA, V34.9XXA, V35.4XXA, V35.5XXA, V35.6XXA, V35.7XXA, V35.9XXA, V36.4XXA, V36.5XXA, V36.6XXA, V36.7XXA, V36.9XXA, V37.4XXA, V37.5XXA, V37.6XXA, V37.7XXA, V37.9XXA, V38.4XXA, V38.5XXA, V38.6XXA, V38.7XXA, V38.9XXA, V39.40XA, V39.49XA, V39.50XA, V39.59XA, V39.60XA, V39.69XA, V39.81XA, V39.89XA, V39.9XXA, V40.4XXA, V40.5XXA, V40.6XXA, V40.7XXA, V40.9XXA, V41.4XXA, V41.5XXA, V41.6XXA, V41.7XXA, V41.9XXA, V42.4XXA, V42.5XXA, V42.6XXA, V42.7XXA, V42.9XXA, V43.41XA, V43.42XA, V43.43XA, V43.44XA, V43.51XA, V43.52XA, V43.53XA, V43.54XA, V43.61XA, V43.62XA, V43.63XA, V43.64XA, V43.71XA, V43.72XA, V43.73XA, V43.74XA, V43.91XA, V43.92XA, V43.93XA, V43.94XA, V44.4XXA, V44.5XXA, V44.6XXA, V44.7XXA, V44.9XXA, V58.9XXA, V59.40XA, V59.49XA, V59.50XA, V59.59XA, V59.60XA, V59.69XA, V59.81XA, V59.88XA, V59.9XXA, V60.4XXA, V60.5XXA, V60.6XXA, V60.7XXA, V60.9XXA, V61.4XXA, V61.5XXA, V61.6XXA, V61.7XXA, V61.9XXA, V62.4XXA, V62.5XXA, V62.6XXA, V62.7XXA, V62.9XXA, V63.4XXA, V63.5XXA, V63.6XXA, V63.7XXA, V63.9XXA, V64.4XXA, V64.5XXA, V64.6XXA, V64.7XXA, V64.9XXA, V65.4XXA, V65.5XXA, V65.6XXA, V65.7XXA, V65.9XXA, V66.4XXA, V66.5XXA, V66.6XXA, V66.7XXA, V66.9XXA, V67.4XXA, V67.5XXA, V67.6XXA, V67.7XXA, V67.9XXA, V68.4XXA, V68.5XXA, V68.6XXA, V68.7XXA, V68.9XXA, V69.40XA, V69.49XA, V69.50XA, V69.59XA, V69.60XA, V69.69XA, V69.81XA, V69.88XA, V69.9XXA, V70.4XXA, V70.5XXA, V70.6XXA, V70.7XXA, V70.9XXA, V71.4XXA, V71.5XXA, V71.6XXA, V71.7XXA, V71.9XXA, V72.4XXA, V72.5XXA, V72.6XXA, V72.7XXA, V72.9XXA, V73.4XXA, V73.5XXA, V73.6XXA, V73.7XXA, V73.9XXA, V74.4XXA, V74.5XXA, V74.6XXA, V74.7XXA, V74.9XXA, V75.4XXA, V75.5XXA, V75.6XXA, V75.7XXA, V75.9XXA, V76.4XXA, V76.5XXA, V76.6XXA, V76.7XXA, V76.9XXA, V77.4XXA, V77.5XXA, V77.6XXA, V77.7XXA, V77.9XXA, V78.4XXA, V78.5XXA, V78.6XXA, V78.7XXA, V78.9XXA, V79.40XA, V79.49XA, V79.50XA, V79.59XA, V79.60XA, V79.69XA, V79.81XA, V79.88XA, V79.9XXA, V80.31XA, V80.32XA, V80.41XA, V80.42XA, V80.51XA, V80.52XA, V81.1XXA, V82.1XXA, V83.0XXA, V83.1XXA, V83.2XXA, V83.3XXA, V84.0XXA, V84.1XXA, V84.2XXA, V84.3XXA, V85.0XXA, V85.1XXA, V85.2XXA, V85.3XXA, V86.01XA, V86.02XA, V86.03XA, V86.04XA, V86.05XA, V86.06XA, V86.09XA, V86.11XA, V86.12XA, V86.13XA, V86.14XA, V86.15XA, V86.16XA, V86.19XA, V86.21XA, V86.22XA, V86.23XA, V86.24XA, V86.25XA, V86.26XA, V86.29XA, V86.31XA, V86.32XA, V86.33XA, V86.34XA, V86.35XA, V86.36XA, V86.39XA, V87.0XXA, V87.1XXA, V87.2XXA, V87.3XXA, V87.4XXA, V87.5XXA, V87.6XXA, V87.7XXA, V87.8XXA, V89.2XXA, X81.0XXA, X82.0XXA, X82.1XXA, X82.2XXA, X82.8XXA, Y02.0XXA, Y03.0XXA, Y03.8XXA, Y32.XXXA, V10.0XXA, V10.1XXA, V10.2XXA, V10.3XXA, V10.4XXA, V10.5XXA, V10.9XXA, V11.0XXA, V11.1XXA, V11.2XXA, V11.3XXA, V11.4XXA, V11.5XXA, V11.9XXA, V12.0XXA, V12.1XXA, V12.2XXA, V13.0XXA, V13.1XXA, V13.2XXA, V14.0XXA, V14.1XXA, V14.2XXA, V15.0XXA, V15.1XXA, V15.2XXA, V15.3XXA, V15.4XXA, V15.5XXA, V15.9XXA, V16.0XXA, V16.1XXA, V16.2XXA, V16.3XXA, V16.4XXA, V16.5XXA, V16.9XXA, V17.0XXA, V17.1XXA, V17.2XXA, V17.3XXA, V17.4XXA, V17.5XXA, V17.9XXA, V18.0XXA, V18.1XXA,

© 2025 Wolpaw BJ et al. *JAMA Network Open*.

V18.2XXA, V18.3XXA, V18.4XXA, V18.5XXA, V18.9XXA, V19.00XA, V19.09XA, V19.10XA,
V19.19XA, V19.20XA, V19.29XA, V19.3XXA, V19.81XA, V19.88XA, V01.00XA, V01.01XA,
V01.02XA, V01.031A, V01.038A, V01.09XA, V01.10XA, V01.11XA, V01.12XA, V01.131A,
V01.138A, V01.19XA, V01.90XA, V01.91XA, V01.92XA, V01.931A, V01.938A, V01.99XA,
V02.00XA, V02.01XA, V02.02XA, V02.031A, V02.038A, V02.09XA, V03.00XA, V03.01XA,
V03.02XA, V03.031A, V03.038A, V03.09XA, V04.00XA, V04.01XA, V04.02XA, V04.031A,
V04.038A, V04.09XA, V05.00XA, V05.01XA, V05.02XA, V05.031A, V05.038A, V05.09XA,
V05.10XA, V05.11XA, V05.12XA, V05.131A, V05.138A, V05.19XA, V05.90XA, V05.91XA,
V05.92XA, V05.931A, V05.938A, V05.99XA, V06.00XA, V06.01XA, V06.02XA, V06.031A,
V06.038A, V06.09XA, V06.10XA, V06.11XA, V06.12XA, V06.131A, V06.138A, V06.19XA,
V06.90XA, V06.91XA, V06.92XA, V06.931A, V06.938A, V06.99XA, V09.00XA, V09.01XA,
V09.09XA, V09.1XXA, V09.9XXA, V20.0XXA, V20.1XXA, V20.2XXA, V21.0XXA, V21.1XXA,
V21.2XXA, V22.0XXA, V22.1XXA, V22.2XXA, V23.0XXA, V23.1XXA, V23.2XXA, V24.0XXA,
V24.1XXA, V24.2XXA, V25.0XXA, V25.1XXA, V25.2XXA, V26.0XXA, V26.1XXA, V26.2XXA,
V27.0XXA, V27.1XXA, V27.2XXA, V28.0XXA, V28.1XXA, V28.2XXA, V29.00XA, V29.09XA,
V29.10XA, V29.19XA, V29.20XA, V29.29XA, V29.3XXA, V30.0XXA, V30.1XXA, V30.2XXA,
V30.3XXA, V31.0XXA, V31.1XXA, V31.2XXA, V31.3XXA, V32.0XXA, V32.1XXA, V32.2XXA,
V32.3XXA, V33.0XXA, V33.1XXA, V33.2XXA, V33.3XXA, V34.0XXA, V34.1XXA, V34.2XXA,
V34.3XXA, V35.0XXA, V35.1XXA, V35.2XXA, V35.3XXA, V36.0XXA, V36.1XXA, V36.2XXA,
V36.3XXA, V37.0XXA, V37.1XXA, V37.2XXA, V37.3XXA, V38.0XXA, V38.1XXA, V38.2XXA,
V38.3XXA, V39.00XA, V39.09XA, V39.10XA, V39.19XA, V39.20XA, V39.29XA, V39.3XXA,
V40.0XXA, V40.1XXA, V40.2XXA, V40.3XXA, V41.0XXA, V41.1XXA, V41.2XXA, V41.3XXA,
V42.0XXA, V42.1XXA, V42.2XXA, V42.3XXA, V43.01XA, V43.02XA, V43.03XA, V43.04XA,
V43.11XA, V43.12XA, V43.13XA, V43.14XA, V43.21XA, V43.22XA, V43.23XA, V43.24XA,
V43.31XA, V43.32XA, V43.33XA, V43.34XA, V44.0XXA, V44.1XXA, V44.2XXA, V44.3XXA,
V45.0XXA, V45.1XXA, V45.2XXA, V45.3XXA, V46.0XXA, V46.1XXA, V46.2XXA, V46.3XXA,
V47.01XA, V47.02XA, V47.0XXA, V47.11XA, V47.12XA, V47.1XXA, V47.2XXA, V47.31XA,
V47.32XA, V47.3XXA, V48.0XXA, V48.1XXA, V48.2XXA, V48.3XXA, V49.00XA, V49.09XA,
V49.10XA, V49.19XA, V50.1XXA, V50.2XXA, V50.3XXA, V51.0XXA, V51.1XXA, V51.2XXA,
V51.3XXA, V52.0XXA, V52.1XXA, V52.2XXA, V52.3XXA, V53.0XXA, V53.1XXA, V53.2XXA,
V53.3XXA, V54.0XXA, V54.1XXA, V54.2XXA, V54.3XXA, V55.0XXA, V55.1XXA, V55.2XXA,
V55.3XXA, V56.0XXA, V56.1XXA, V56.2XXA, V56.3XXA, V57.0XXA, V57.1XXA, V57.2XXA,
V57.3XXA, V58.0XXA, V58.1XXA, V58.2XXA, V58.3XXA, V59.00XA, V59.09XA, V59.10XA,
V59.19XA, V59.20XA, V59.29XA, V59.3XXA, V60.0XXA, V60.1XXA, V60.2XXA, V60.3XXA,
V61.0XXA, V61.1XXA, V61.2XXA, V61.3XXA, V62.0XXA, V62.1XXA, V62.2XXA, V62.3XXA,
V63.0XXA, V63.1XXA, V63.2XXA, V63.3XXA, V64.0XXA, V64.1XXA, V64.2XXA, V64.3XXA,
V65.0XXA, V65.1XXA, V65.2XXA, V65.3XXA, V66.0XXA, V66.1XXA, V66.2XXA, V66.3XXA,
V67.0XXA, V67.1XXA, V67.2XXA, V67.3XXA, V68.0XXA, V68.1XXA, V68.2XXA, V68.3XXA,
V69.00XA, V69.09XA, V69.10XA, V69.19XA, V69.20XA, V69.29XA, V69.3XXA, V70.0XXA,
V70.1XXA, V70.2XXA, V70.3XXA, V71.0XXA, V71.1XXA, V71.2XXA, V71.3XXA, V72.0XXA,
V72.1XXA, V72.2XXA, V72.3XXA, V73.0XXA, V73.1XXA, V73.2XXA, V73.3XXA, V74.0XXA,
V74.1XXA, V74.2XXA, V74.3XXA, V75.0XXA, V75.1XXA, V75.2XXA, V75.3XXA, V76.0XXA,
V76.1XXA, V76.2XXA, V76.3XXA, V77.0XXA, V77.1XXA, V77.2XXA, V77.3XXA, V78.0XXA,
V78.1XXA, V78.2XXA, V78.3XXA, V79.00XA, V79.09XA, V79.10XA, V79.19XA, V79.20XA,
V79.29XA, V79.3XXA, V80.010A, V80.018A, V80.02XA, V80.11XA, V80.12XA, V80.21XA,
V80.22XA, V80.61XA, V80.62XA, V80.710A, V80.711A, V80.720A, V80.721A, V80.730A,
V80.731A, V80.790A, V80.791A, V80.81XA, V80.82XA, V80.910A, V80.918A, V80.919A,
V80.920A, V80.928A, V80.929A, V81.0XXA, V81.2XXA, V81.3XXA, V81.4XXA, V81.5XXA,
V81.6XXA, V81.7XXA, V81.81XA, V81.82XA, V81.83XA, V81.89XA, V81.9XXA, V82.0XXA,
V82.2XXA, V82.3XXA, V82.4XXA, V82.5XXA, V82.6XXA, V82.7XXA, V82.8XXA, V82.9XXA,
V83.4XXA, V83.5XXA, V83.6XXA, V83.7XXA, V83.9XXA, V84.4XXA, V84.5XXA, V84.6XXA,
V84.7XXA, V84.9XXA, V85.4XXA, V85.5XXA, V85.6XXA, V85.7XXA, V85.9XXA, V86.41XA,
V86.42XA, V86.43XA, V86.44XA, V86.45XA, V86.46XA, V86.49XA, V86.51XA, V86.52XA,
V86.53XA, V86.54XA, V86.55XA, V86.56XA, V86.59XA, V86.61XA, V86.62XA, V86.63XA,
V86.64XA, V86.65XA, V86.66XA, V86.69XA, V86.71XA, V86.72XA, V86.73XA, V86.74XA,
V86.75XA, V86.76XA, V86.79XA, V86.91XA, V86.92XA, V86.93XA, V86.94XA, V86.95XA,
V86.96XA, V86.99XA, V87.9XXA, V88.0XXA, V88.1XXA, V88.2XXA, V88.3XXA, V88.4XXA,
V88.5XXA, V88.6XXA, V88.7XXA, V88.8XXA, V88.9XXA, V89.0XXA, V89.1XXA, V89.3XXA,
V89.9XXA, V91.00XA, V91.01XA, V91.02XA, V91.03XA, V91.04XA, V91.05XA, V91.06XA,
V91.07XA, V91.08XA, V91.09XA, V91.10XA, V91.11XA, V91.12XA, V91.13XA, V91.14XA,
V91.15XA, V91.16XA, V91.18XA, V91.19XA, V91.20XA, V91.21XA, V91.22XA, V91.23XA,

V91.24XA, V91.25XA, V91.26XA, V91.29XA, V91.30XA, V91.31XA, V91.32XA, V91.33XA, V91.34XA, V91.35XA, V91.36XA, V91.37XA, V91.38XA, V91.39XA, V91.80XA, V91.81XA, V91.82XA, V91.83XA, V91.84XA, V91.85XA, V91.86XA, V91.87XA, V91.88XA, V91.89XA, V93.00XA, V93.01XA, V93.02XA, V93.03XA, V93.04XA, V93.09XA, V93.10XA, V93.11XA, V93.12XA, V93.13XA, V93.14XA, V93.19XA, V93.20XA, V93.21XA, V93.22XA, V93.23XA, V93.24XA, V93.29XA, V93.30XA, V93.31XA, V93.32XA, V93.33XA, V93.34XA, V93.35XA, V93.36XA, V93.38XA, V93.39XA, V93.40XA, V93.41XA, V93.42XA, V93.43XA, V93.44XA, V93.48XA, V93.49XA, V93.50XA, V93.51XA, V93.52XA, V93.53XA, V93.54XA, V93.59XA, V93.60XA, V93.61XA, V93.62XA, V93.63XA, V93.64XA, V93.69XA, V93.80XA, V93.81XA, V93.82XA, V93.83XA, V93.84XA, V93.85XA, V93.86XA, V93.87XA, V93.88XA, V93.89XA, V94.0XXA, V94.11XA, V94.12XA, V94.21XA, V94.22XA, V94.31XA, V94.32XA, V94.4XXA, V94.810A, V94.811A, V94.818A, V94.89XA, V94.9XXA, V95.00XA, V95.01XA, V95.02XA, V95.03XA, V95.04XA, V95.05XA, V95.09XA, V95.10XA, V95.11XA, V95.12XA, V95.13XA, V95.14XA, V95.15XA, V95.19XA, V95.20XA, V95.21XA, V95.22XA, V95.23XA, V95.24XA, V95.25XA, V95.29XA, V95.30XA, V95.31XA, V95.32XA, V95.33XA, V95.34XA, V95.35XA, V95.39XA, V95.40XA, V95.41XA, V95.42XA, V95.43XA, V95.44XA, V95.45XA, V95.49XA, V95.8XXA, V95.9XXA, V96.00XA, V96.01XA, V96.02XA, V96.03XA, V96.04XA, V96.05XA, V96.09XA, V96.10XA, V96.11XA, V96.12XA, V96.13XA, V96.14XA, V96.15XA, V96.19XA, V96.20XA, V96.21XA, V96.22XA, V96.23XA, V96.24XA, V96.25XA, V96.29XA, V96.8XXA, V96.9XXA, V97.0XXA, V97.1XXA, V97.21XA, V97.22XA, V97.29XA, V97.31XA, V97.32XA, V97.33XA, V97.39XA, V97.810A, V97.811A, V97.818A, V97.89XA, V98.0XXA, V98.1XXA, V98.2XXA, V98.3XXA, V98.8XXA, V99.XXXA, X81.1XXA, X83.0XXA, Y02.1XXA, Y08.81XA, Y36.100A, Y36.101A, Y36.110A, Y36.111A, Y36.120A, Y36.121A, Y36.130A, Y36.131A, Y36.140A, Y36.141A, Y36.190A, Y36.191A, Y37.100A, Y37.101A, Y37.110A, Y37.111A, Y37.120A, Y37.121A, Y37.130A, Y37.131A, Y37.140A, Y37.141A, Y37.190A, Y37.191A, Y38.1X1A, Y38.1X2A, Y38.1X3A, T63.711A, T63.712A, T63.713A, T63.714A, T63.791A, T63.792A, T63.793A, T63.794A, T65.821A, T65.822A, T65.823A, T65.824A, W42.0XXA, W42.9XXA, W53.09XA, W53.19XA, W53.29XA, W53.89XA, W54.1XXA, W54.8XXA, W55.03XA, W55.09XA, W55.12XA, W55.19XA, W55.22XA, W55.29XA, W55.32XA, W55.39XA, W55.42XA, W55.49XA, W55.52XA, W55.59XA, W55.82XA, W55.89XA, W56.02XA, W56.09XA, W56.12XA, W56.19XA, W56.22XA, W56.29XA, W56.32XA, W56.39XA, W56.42XA, W56.49XA, W56.52XA, W56.59XA, W56.82XA, W56.89XA, W58.02XA, W58.03XA, W58.09XA, W58.12XA, W58.13XA, W58.19XA, W59.02XA, W59.09XA, W59.12XA, W59.13XA, W59.19XA, W59.22XA, W59.29XA, W59.82XA, W59.83XA, W59.89XA, W60.XXXA, W61.02XA, W61.09XA, W61.12XA, W61.19XA, W61.22XA, W61.29XA, W61.32XA, W61.33XA, W61.39XA, W61.42XA, W61.43XA, W61.49XA, W61.52XA, W61.59XA, W61.62XA, W61.69XA, W61.92XA, W61.99XA, W62.0XXA, W62.1XXA, W62.9XXA, W64.XXXA, W92.XXXA, W93.01XA, W93.02XA, W93.11XA, W93.12XA, W93.2XXA, W93.8XXA, W94.0XXA, W94.11XA, W94.12XA, W94.21XA, W94.22XA, W94.23XA, W94.29XA, W94.31XA, W94.32XA, W94.39XA, W99.XXXA, X30.XXXA, X31.XXXA, X32.XXXA, X34.XXXA, X35.XXXA, X36.0XXA, X36.1XXA, X37.0XXA, X37.1XXA, X37.2XXA, X37.3XXA, X37.41XA, X37.42XA, X37.43XA, X37.8XXA, X37.9XXA, X38.XXXA, X39.01XA, X39.08XA, X39.8XXA, X52.XXXA, X83.2XXA, T63.002A, T63.003A, T63.004A, T63.012A, T63.013A, T63.014A, T63.022A, T63.023A, T63.024A, T63.032A, T63.033A, T63.034A, T63.042A, T63.043A, T63.044A, T63.062A, T63.063A, T63.064A, T63.072A, T63.073A, T63.074A, T63.082A, T63.083A, T63.084A, T63.092A, T63.093A, T63.094A, T63.112A, T63.113A, T63.114A, T63.122A, T63.123A, T63.124A, T63.192A, T63.193A, T63.194A, T63.2X2A, T63.2X3A, T63.2X4A, T63.302A, T63.303A, T63.304A, T63.312A, T63.313A, T63.314A, T63.322A, T63.323A, T63.324A, T63.332A, T63.333A, T63.334A, T63.392A, T63.393A, T63.394A, T63.412A, T63.413A, T63.414A, T63.421A, T63.422A, T63.423A, T63.424A, T63.431A, T63.432A, T63.433A, T63.434A, T63.441A, T63.442A, T63.443A, T63.444A, T63.451A, T63.452A, T63.453A, T63.454A, T63.461A, T63.462A, T63.463A, T63.464A, T63.481A, T63.482A, T63.483A, T63.484A, T63.511A, T63.512A, T63.513A, T63.514A, T63.591A, T63.592A, T63.593A, T63.594A, T63.611A, T63.612A, T63.613A, T63.614A, T63.621A, T63.622A, T63.623A, T63.624A, T63.631A, T63.632A, T63.633A, T63.634A, T63.691A, T63.692A, T63.693A, T63.694A, T63.811A, T63.812A, T63.813A, T63.814A, T63.821A, T63.822A, T63.823A, T63.824A, T63.831A, T63.832A, T63.833A, T63.834A, T63.891A, T63.892A, T63.893A, T63.894A, T63.91XA, T63.92XA, T63.93XA, T63.94XA, W53.01XA, W53.11XA, W53.21XA, W53.81XA, W54.0XXA, W55.01XA, W55.11XA, W55.21XA, W55.31XA, W55.41XA, W55.51XA, W55.81XA, W56.01XA, W56.11XA, W56.21XA, W56.31XA, W56.41XA, W56.51XA, W56.81XA, W57.XXXA, W58.01XA, W58.11XA, W59.01XA, W59.11XA, W59.21XA, W59.81XA, W61.01XA, W61.11XA, W61.21XA, W61.51XA, W61.61XA, W61.91XA, W18.40XA, W18.41XA, W18.42XA, W18.43XA, W18.49XA, X50.0XXA, X50.1XXA, X50.3XXA, X50.9XXA, V00.01XA, V00.02XA, V00.031A, V00.038A, V00.09XA,

© 2025 Wolpaw BJ et al. *JAMA Network Open*.

V00.112A, V00.122A, V00.132A, V00.142A, V00.152A, V00.182A, V00.212A, V00.222A, V00.282A, V00.312A, V00.322A, V00.382A, V00.812A, V00.822A, V00.832A, V00.842A, V00.892A, W18.00XA, W18.01XA, W18.02XA, W18.09XA, W20.0XXA, W20.1XXA, W20.8XXA, W21.00XA, W21.01XA, W21.02XA, W21.03XA, W21.04XA, W21.05XA, W21.06XA, W21.07XA, W21.09XA, W21.11XA, W21.12XA, W21.13XA, W21.19XA, W21.210A, W21.211A, W21.220A, W21.221A, W21.31XA, W21.32XA, W21.39XA, W21.4XXA, W21.81XA, W21.89XA, W21.9XXA, W22.01XA, W22.02XA, W22.03XA, W22.042A, W22.09XA, W22.10XA, W22.11XA, W22.12XA, W22.19XA, W22.8XXA, W50.0XXA, W50.1XXA, W50.2XXA, W50.3XXA, W50.4XXA, W51.XXXA, W52.XXXA, X79.XXXA, Y00.XXXA, Y04.0XXA, Y04.1XXA, Y04.2XXA, Y04.8XXA, Y08.01XA, Y08.02XA, Y08.09XA, Y29.XXXA, Y35.301A, Y35.302A, Y35.303A, Y35.309A, Y35.311A, Y35.312A, Y35.313A, Y35.319A, Y35.391A, Y35.392A, Y35.393A, Y35.399A, Y35.811A, Y35.812A, Y35.813A, Y35.819A, Y36.440A, Y36.441A, Y37.440A, Y37.441A, T17.200A, T17.210A, T17.220A, T17.290A, T17.300A, T17.310A, T17.320A, T17.390A, T17.400A, T17.410A, T17.420A, T17.490A, T17.500A, T17.510A, T17.520A, T17.590A, T17.800A, T17.810A, T17.820A, T17.890A, T17.900A, T17.910A, T17.920A, T17.990A, Y36.460A, Y36.461A, Y36.470A, Y36.471A, Y37.460A, Y37.461A, Y37.470A, Y37.471A, T15.00XA, T15.01XA, T15.02XA, T15.10XA, T15.11XA, T15.12XA, T15.80XA, T15.81XA, T15.82XA, T15.90XA, T15.91XA, T15.92XA, T16.1XXA, T16.2XXA, T16.9XXA, T17.0XXA, T17.1XXA, T17.208A, T17.218A, T17.228A, T17.298A, T17.308A, T17.318A, T17.328A, T17.398A, T17.408A, T17.418A, T17.428A, T17.498A, T17.508A, T17.518A, T17.528A, T17.598A, T17.808A, T17.818A, T17.828A, T17.898A, T17.908A, T17.918A, T17.928A, T17.998A, T18.0XXA, T18.100A, T18.108A, T18.110A, T18.118A, T18.120A, T18.128A, T18.190A, T18.198A, T18.2XXA, T18.3XXA, T18.4XXA, T18.5XXA, T18.8XXA, T18.9XXA, T19.0XXA, T19.1XXA, T19.2XXA, T19.3XXA, T19.4XXA, T19.8XXA, T19.9XXA, T74.01XA, T74.02XA, T74.11XA, T74.12XA, T74.21XA, T74.22XA, T74.31XA, T74.32XA, T74.4XXA, T74.91XA, T74.92XA, T76.01XA, T76.02XA, T76.11XA, T76.12XA, T76.21XA, T76.22XA, T76.31XA, T76.32XA, T76.91XA, T76.92XA, V00.118A, V00.128A, V00.138A, V00.148A, V00.158A, V00.188A, V00.218A, V00.228A, V00.288A, V00.318A, V00.328A, V00.388A, V00.818A, V00.828A, V00.838A, V00.848A, V00.898A, W23.0XXA, W23.1XXA, W34.010A, W34.011A, W34.018A, W34.110A, W34.111A, W34.118A, W35.XXXA, W36.1XXA, W36.2XXA, W36.3XXA, W36.8XXA, W36.9XXA, W37.0XXA, W37.8XXA, W38.XXXA, W39.XXXA, W40.0XXA, W40.1XXA, W40.8XXA, W40.9XXA, W49.01XA, W49.02XA, W49.03XA, W49.04XA, W49.09XA, W49.9XXA, W85.XXXA, W86.0XXA, W86.1XXA, W86.8XXA, W88.0XXA, W88.1XXA, W88.8XXA, W89.0XXA, W89.1XXA, W89.8XXA, W89.9XXA, W90.0XXA, W90.1XXA, W90.2XXA, W90.8XXA, X74.01XA, X74.02XA, X74.09XA, X75.XXXA, X81.8XXA, X83.1XXA, X83.8XXA, X95.01XA, X95.02XA, X95.09XA, X96.0XXA, X96.1XXA, X96.2XXA, X96.3XXA, X96.4XXA, X96.8XXA, X96.9XXA, Y02.8XXA, Y07.01, Y07.02, Y07.03, Y07.04, Y07.11, Y07.12, Y07.13, Y07.14, Y07.410, Y07.411, Y07.420, Y07.421, Y07.430, Y07.432, Y07.433, Y07.434, Y07.435, Y07.436, Y07.490, Y07.491, Y07.499, Y07.50, Y07.510, Y07.511, Y07.512, Y07.513, Y07.519, Y07.521, Y07.528, Y07.529, Y07.53, Y07.59, Y07.6, Y07.9, Y08.89XA, Y24.0XXA, Y25.XXXA, Y31.XXXA, Y33.XXXA, Y35.041A, Y35.042A, Y35.043A, Y35.049A, Y35.101A, Y35.102A, Y35.103A, Y35.109A, Y35.111A, Y35.112A, Y35.113A, Y35.119A, Y35.121A, Y35.122A, Y35.123A, Y35.129A, Y35.191A, Y35.192A, Y35.193A, Y35.199A, Y35.831A, Y35.832A, Y35.833A, Y35.839A, Y35.891A, Y35.892A, Y35.893A, Y36.000A, Y36.001A, Y36.010A, Y36.011A, Y36.020A, Y36.021A, Y36.030A, Y36.031A, Y36.040A, Y36.041A, Y36.050A, Y36.051A, Y36.090A, Y36.091A, Y36.200A, Y36.201A, Y36.210A, Y36.211A, Y36.220A, Y36.221A, Y36.230A, Y36.231A, Y36.240A, Y36.241A, Y36.250A, Y36.251A, Y36.260A, Y36.261A, Y36.270A, Y36.271A, Y36.290A, Y36.291A, Y36.310A, Y36.311A, Y36.320A, Y36.321A, Y36.410A, Y36.411A, Y36.450A, Y36.451A, Y36.490A, Y36.491A, Y36.500A, Y36.501A, Y36.510A, Y36.511A, Y36.520A, Y36.521A, Y36.530A, Y36.531A, Y36.540A, Y36.541A, Y36.590A, Y36.591A, Y36.6X0A, Y36.6X1A, Y36.810A, Y36.811A, Y36.820A, Y36.821A, Y36.880A, Y36.881A, Y36.91XA, Y37.000A, Y37.001A, Y37.010A, Y37.011A, Y37.020A, Y37.021A, Y37.030A, Y37.031A, Y37.040A, Y37.041A, Y37.050A, Y37.051A, Y37.090A, Y37.091A, Y37.200A, Y37.201A, Y37.210A, Y37.211A, Y37.220A, Y37.221A, Y37.230A, Y37.231A, Y37.240A, Y37.241A, Y37.250A, Y37.251A, Y37.260A, Y37.261A, Y37.270A, Y37.271A, Y37.290A, Y37.291A, Y37.310A, Y37.311A, Y37.320A, Y37.321A, Y37.410A, Y37.411A, Y37.450A, Y37.451A, Y37.490A, Y37.491A, Y37.500A, Y37.501A, Y37.510A, Y37.511A, Y37.520A, Y37.521A, Y37.530A, Y37.531A, Y37.540A, Y37.541A, Y37.590A, Y37.591A, Y37.6X0A, Y37.6X1A, Y37.91XA, Y38.0X1A, Y38.0X2A, Y38.0X3A, Y38.2X1A, Y38.2X2A, Y38.2X3A, Y38.5X1A, Y38.5X2A, Y38.5X3A, Y38.6X1A, Y38.6X2A, Y38.6X3A, Y38.811A, Y38.812A, Y38.891A, Y38.892A, Y38.893A, Y38.9X1A, Y38.9X2A, X58.XXXA, Y09, Y35.91XA, Y35.92XA, Y35.93XA, Y35.99XA, Y36.890A, Y36.891A, Y36.90XA, Y37.90XA, Y38.80XA,

© 2025 Wolpaw BJ et al. *JAMA Network Open*.

S12.000A, S12.000B, S12.001A, S12.001B, S12.01XA, S12.01XB, S12.02XA, S12.02XB,
S12.030A, S12.030B, S12.031A, S12.031B, S12.040A, S12.040B, S12.041A, S12.041B,
S12.090A, S12.090B, S12.091A, S12.091B, S12.100A, S12.100B, S12.101A, S12.101B,
S12.110A, S12.110B, S12.111A, S12.111B, S12.112A, S12.112B, S12.120A, S12.120B,
S12.121A, S12.121B, S12.130A, S12.130B, S12.131A, S12.131B, S12.14XA, S12.14XB,
S12.150A, S12.150B, S12.151A, S12.151B, S12.190A, S12.190B, S12.191A, S12.191B,
S12.200A, S12.200B, S12.201A, S12.201B, S12.230A, S12.230B, S12.231A, S12.231B,
S12.24XA, S12.24XB, S12.250A, S12.250B, S12.251A, S12.251B, S12.290A, S12.290B,
S12.291A, S12.291B, S12.300A, S12.300B, S12.301A, S12.301B, S12.330A, S12.330B,
S12.331A, S12.331B, S12.34XA, S12.34XB, S12.350A, S12.350B, S12.351A, S12.351B,
S12.390A, S12.390B, S12.391A, S12.391B, S12.400A, S12.400B, S12.401A, S12.401B,
S12.430A, S12.430B, S12.431A, S12.431B, S12.44XA, S12.44XB, S12.450A, S12.450B,
S12.451A, S12.451B, S12.490A, S12.490B, S12.491A, S12.491B, S12.500A, S12.500B,
S12.501A, S12.501B, S12.530A, S12.530B, S12.531A, S12.531B, S12.54XA, S12.54XB,
S12.550A, S12.550B, S12.551A, S12.551B, S12.590A, S12.590B, S12.591A, S12.591B,
S12.600A, S12.600B, S12.601A, S12.601B, S12.630A, S12.630B, S12.631A, S12.631B,
S12.64XA, S12.64XB, S12.650A, S12.650B, S12.651A, S12.651B, S12.690A, S12.690B,
S12.691A, S12.691B, S12.8XXA, S12.9XXA, S22.000A, S22.000B, S22.001A, S22.001B,
S22.002A, S22.002B, S22.008A, S22.008B, S22.009A, S22.009B, S22.010A, S22.010B,
S22.011A, S22.011B, S22.012A, S22.012B, S22.018A, S22.018B, S22.019A, S22.019B,
S22.020A, S22.020B, S22.021A, S22.021B, S22.022A, S22.022B, S22.028A, S22.028B,
S22.029A, S22.029B, S22.030A, S22.030B, S22.031A, S22.031B, S22.032A, S22.032B,
S22.038A, S22.038B, S22.039A, S22.039B, S22.040A, S22.040B, S22.041A, S22.041B,
S22.042A, S22.042B, S22.048A, S22.048B, S22.049A, S22.049B, S22.050A, S22.050B,
S22.051A, S22.051B, S22.052A, S22.052B, S22.058A, S22.058B, S22.059A, S22.059B,
S22.060A, S22.060B, S22.061A, S22.061B, S22.062A, S22.062B, S22.068A, S22.068B,
S22.069A, S22.069B, S22.070A, S22.070B, S22.071A, S22.071B, S22.072A, S22.072B,
S22.078A, S22.078B, S22.079A, S22.079B, S22.080A, S22.080B, S22.081A, S22.081B,
S22.082A, S22.082B, S22.088A, S22.088B, S22.089A, S22.089B, S32.000A, S32.000B,
S32.001A, S32.001B, S32.002A, S32.002B, S32.008A, S32.008B, S32.009A, S32.009B,
S32.010A, S32.010B, S32.011A, S32.011B, S32.012A, S32.012B, S32.018A, S32.018B,
S32.019A, S32.019B, S32.020A, S32.020B, S32.021A, S32.021B, S32.022A, S32.022B,
S32.028A, S32.028B, S32.029A, S32.029B, S32.030A, S32.030B, S32.031A, S32.031B,
S32.032A, S32.032B, S32.038A, S32.038B, S32.039A, S32.039B, S32.040A, S32.040B,
S32.041A, S32.041B, S32.042A, S32.042B, S32.048A, S32.048B, S32.049A, S32.049B,
S32.050A, S32.050B, S32.051A, S32.051B, S32.052A, S32.052B, S32.058A, S32.058B,
S32.059A, S32.059B, S32.10XA, S32.10XB, S32.110A, S32.110B, S32.111A, S32.111B,
S32.112A, S32.112B, S32.119A, S32.119B, S32.120A, S32.120B, S32.121A, S32.121B,
S32.122A, S32.122B, S32.129A, S32.129B, S32.130A, S32.130B, S32.131A, S32.131B,
S32.132A, S32.132B, S32.139A, S32.139B, S32.14XA, S32.14XB, S32.15XA, S32.15XB,
S32.16XA, S32.16XB, S32.17XA, S32.17XB, S32.19XA, S32.19XB, S32.2XXA, S32.2XXB,
S32.9XXA, S32.9XXB, S22.20XA, S22.20XB, S22.21XA, S22.21XB, S22.22XA, S22.22XB,
S22.23XA, S22.23XB, S22.24XA, S22.24XB, S22.31XA, S22.31XB, S22.32XA, S22.32XB,
S22.39XA, S22.39XB, S22.41XA, S22.41XB, S22.42XA, S22.42XB, S22.43XA, S22.43XB,
S22.49XA, S22.49XB, S22.5XXA, S22.5XXB, S22.9XXA, S22.9XXB, S32.301A, S32.301B,
S32.302A, S32.302B, S32.309A, S32.309B, S32.311A, S32.311B, S32.312A, S32.312B,
S32.313A, S32.313B, S32.314A, S32.314B, S32.315A, S32.315B, S32.316A, S32.316B,
S32.391A, S32.391B, S32.392A, S32.392B, S32.399A, S32.399B, S32.401A, S32.401B,
S32.402A, S32.402B, S32.409A, S32.409B, S32.411A, S32.411B, S32.412A, S32.412B,
S32.413A, S32.413B, S32.414A, S32.414B, S32.415A, S32.415B, S32.416A, S32.416B,
S32.421A, S32.421B, S32.422A, S32.422B, S32.423A, S32.423B, S32.424A, S32.424B,
S32.425A, S32.425B, S32.426A, S32.426B, S32.431A, S32.431B, S32.432A, S32.432B,
S32.433A, S32.433B, S32.434A, S32.434B, S32.435A, S32.435B, S32.436A, S32.436B,
S32.441A, S32.441B, S32.442A, S32.442B, S32.443A, S32.443B, S32.444A, S32.444B,
S32.445A, S32.445B, S32.446A, S32.446B, S32.451A, S32.451B, S32.452A, S32.452B,
S32.453A, S32.453B, S32.454A, S32.454B, S32.455A, S32.455B, S32.456A, S32.456B,
S32.461A, S32.461B, S32.462A, S32.462B, S32.463A, S32.463B, S32.464A, S32.464B,
S32.465A, S32.465B, S32.466A, S32.466B, S32.471A, S32.471B, S32.472A, S32.472B,
S32.473A, S32.473B, S32.474A, S32.474B, S32.475A, S32.475B, S32.476A, S32.476B,
S32.481A, S32.481B, S32.482A, S32.482B, S32.483A, S32.483B, S32.484A, S32.484B,
S32.485A, S32.485B, S32.486A, S32.486B, S32.491A, S32.491B, S32.492A, S32.492B,

S32.499A, S32.499B, S32.501A, S32.501B, S32.502A, S32.502B, S32.509A, S32.509B, S32.511A, S32.511B, S32.512A, S32.512B, S32.519A, S32.519B, S32.591A, S32.591B, S32.592A, S32.592B, S32.599A, S32.599B, S32.601A, S32.601B, S32.602A, S32.602B, S32.609A, S32.609B, S32.611A, S32.611B, S32.612A, S32.612B, S32.613A, S32.613B, S32.614A, S32.614B, S32.615A, S32.615B, S32.616A, S32.616B, S32.691A, S32.691B, S32.692A, S32.692B, S32.699A, S32.699B, S32.810A, S32.810B, S32.811A, S32.811B, S32.82XA, S32.82XB, S32.89XA, S32.89XB, M97.31XA, M97.32XA, M97.41XA, M97.42XA, S42.001A, S42.001B, S42.002A, S42.002B, S42.009A, S42.009B, S42.011A, S42.011B, S42.012A, S42.012B, S42.013A, S42.013B, S42.014A, S42.014B, S42.015A, S42.015B, S42.016A, S42.016B, S42.017A, S42.017B, S42.018A, S42.018B, S42.019A, S42.019B, S42.021A, S42.021B, S42.022A, S42.022B, S42.023A, S42.023B, S42.024A, S42.024B, S42.025A, S42.025B, S42.026A, S42.026B, S42.031A, S42.031B, S42.032A, S42.032B, S42.033A, S42.033B, S42.034A, S42.034B, S42.035A, S42.035B, S42.036A, S42.036B, S42.101A, S42.101B, S42.102A, S42.102B, S42.109A, S42.109B, S42.111A, S42.111B, S42.112A, S42.112B, S42.113A, S42.113B, S42.114A, S42.114B, S42.115A, S42.115B, S42.116A, S42.116B, S42.121A, S42.121B, S42.122A, S42.122B, S42.123A, S42.123B, S42.124A, S42.124B, S42.125A, S42.125B, S42.126A, S42.126B, S42.131A, S42.131B, S42.132A, S42.132B, S42.133A, S42.133B, S42.134A, S42.134B, S42.135A, S42.135B, S42.136A, S42.136B, S42.141A, S42.141B, S42.142A, S42.142B, S42.143A, S42.143B, S42.144A, S42.144B, S42.145A, S42.145B, S42.146A, S42.146B, S42.151A, S42.151B, S42.152A, S42.152B, S42.153A, S42.153B, S42.154A, S42.154B, S42.155A, S42.155B, S42.156A, S42.156B, S42.191A, S42.191B, S42.192A, S42.192B, S42.199A, S42.199B, S42.201A, S42.201B, S42.202A, S42.202B, S42.209A, S42.209B, S42.211A, S42.211B, S42.212A, S42.212B, S42.213A, S42.213B, S42.214A, S42.214B, S42.215A, S42.215B, S42.216A, S42.216B, S42.221A, S42.221B, S42.222A, S42.222B, S42.223A, S42.223B, S42.224A, S42.224B, S42.225A, S42.225B, S42.226A, S42.226B, S42.231A, S42.231B, S42.232A, S42.232B, S42.239A, S42.239B, S42.241A, S42.241B, S42.242A, S42.242B, S42.249A, S42.249B, S42.251A, S42.251B, S42.252A, S42.252B, S42.253A, S42.253B, S42.254A, S42.254B, S42.255A, S42.255B, S42.256A, S42.256B, S42.261A, S42.261B, S42.262A, S42.262B, S42.263A, S42.263B, S42.264A, S42.264B, S42.265A, S42.265B, S42.266A, S42.266B, S42.271A, S42.272A, S42.279A, S42.291A, S42.291B, S42.292A, S42.292B, S42.293A, S42.293B, S42.294A, S42.294B, S42.295A, S42.295B, S42.296A, S42.296B, S42.301A, S42.301B, S42.302A, S42.302B, S42.309A, S42.309B, S42.311A, S42.312A, S42.319A, S42.321A, S42.321B, S42.322A, S42.322B, S42.323A, S42.323B, S42.324A, S42.324B, S42.325A, S42.325B, S42.326A, S42.326B, S42.331A, S42.331B, S42.332A, S42.332B, S42.333A, S42.333B, S42.334A, S42.334B, S42.335A, S42.335B, S42.336A, S42.336B, S42.341A, S42.341B, S42.342A, S42.342B, S42.343A, S42.343B, S42.344A, S42.344B, S42.345A, S42.345B, S42.346A, S42.346B, S42.351A, S42.351B, S42.352A, S42.352B, S42.353A, S42.353B, S42.354A, S42.354B, S42.355A, S42.355B, S42.356A, S42.356B, S42.361A, S42.361B, S42.362A, S42.362B, S42.363A, S42.363B, S42.364A, S42.364B, S42.365A, S42.365B, S42.366A, S42.366B, S42.391A, S42.391B, S42.392A, S42.392B, S42.399A, S42.399B, S42.401A, S42.401B, S42.402A, S42.402B, S42.409A, S42.409B, S42.411A, S42.411B, S42.412A, S42.412B, S42.413A, S42.413B, S42.414A, S42.414B, S42.415A, S42.415B, S42.416A, S42.416B, S42.421A, S42.421B, S42.422A, S42.422B, S42.423A, S42.423B, S42.424A, S42.424B, S42.425A, S42.425B, S42.426A, S42.426B, S42.431A, S42.431B, S42.432A, S42.432B, S42.433A, S42.433B, S42.434A, S42.434B, S42.435A, S42.435B, S42.436A, S42.436B, S42.441A, S42.441B, S42.442A, S42.442B, S42.443A, S42.443B, S42.444A, S42.444B, S42.445A, S42.445B, S42.446A, S42.446B, S42.447A, S42.447B, S42.448A, S42.448B, S42.449A, S42.449B, S42.451A, S42.451B, S42.452A, S42.452B, S42.453A, S42.453B, S42.454A, S42.454B, S42.455A, S42.455B, S42.456A, S42.456B, S42.461A, S42.461B, S42.462A, S42.462B, S42.463A, S42.463B, S42.464A, S42.464B, S42.465A, S42.465B, S42.466A, S42.466B, S42.471A, S42.471B, S42.472A, S42.472B, S42.473A, S42.473B, S42.474A, S42.474B, S42.475A, S42.475B, S42.476A, S42.476B, S42.481A, S42.482A, S42.489A, S42.491A, S42.491B, S42.492A, S42.492B, S42.493A, S42.493B, S42.494A, S42.494B, S42.495A, S42.495B, S42.496A, S42.496B, S42.90XA, S42.90XB, S42.91XA, S42.91XB, S42.92XA, S42.92XB, S49.001A, S49.002A, S49.009A, S49.011A, S49.012A, S49.019A, S49.021A, S49.022A, S49.029A, S49.031A, S49.032A, S49.039A, S49.041A, S49.042A, S49.049A, S49.091A, S49.092A, S49.099A, S49.101A, S49.102A, S49.109A, S49.111A, S49.112A, S49.119A, S49.121A, S49.122A, S49.129A, S49.131A, S49.132A, S49.139A, S49.141A, S49.142A, S49.149A, S49.191A, S49.192A, S49.199A, S52.001A, S52.001B, S52.001C,

© 2025 Wolpaw BJ et al. *JAMA Network Open*.

S52.002A, S52.002B, S52.002C, S52.009A, S52.009B, S52.009C, S52.011A, S52.012A, S52.019A, S52.021A, S52.021B, S52.021C, S52.022A, S52.022B, S52.022C, S52.023A, S52.023B, S52.023C, S52.024A, S52.024B, S52.024C, S52.025A, S52.025B, S52.025C, S52.026A, S52.026B, S52.026C, S52.031A, S52.031B, S52.031C, S52.032A, S52.032B, S52.032C, S52.033A, S52.033B, S52.033C, S52.034A, S52.034B, S52.034C, S52.035A, S52.035B, S52.035C, S52.036A, S52.036B, S52.036C, S52.041A, S52.041B, S52.041C, S52.042A, S52.042B, S52.042C, S52.043A, S52.043B, S52.043C, S52.044A, S52.044B, S52.044C, S52.045A, S52.045B, S52.045C, S52.046A, S52.046B, S52.046C, S52.091A, S52.091B, S52.091C, S52.092A, S52.092B, S52.092C, S52.099A, S52.099B, S52.099C, S52.101A, S52.101B, S52.101C, S52.102A, S52.102B, S52.102C, S52.109A, S52.109B, S52.109C, S52.111A, S52.112A, S52.119A, S52.121A, S52.121B, S52.121C, S52.122A, S52.122B, S52.122C, S52.123A, S52.123B, S52.123C, S52.124A, S52.124B, S52.124C, S52.125A, S52.125B, S52.125C, S52.126A, S52.126B, S52.126C, S52.131A, S52.131B, S52.131C, S52.132A, S52.132B, S52.132C, S52.133A, S52.133B, S52.133C, S52.134A, S52.134B, S52.134C, S52.135A, S52.135B, S52.135C, S52.136A, S52.136B, S52.136C, S52.181A, S52.181B, S52.181C, S52.182A, S52.182B, S52.182C, S52.189A, S52.189B, S52.189C, S52.201A, S52.201B, S52.201C, S52.202A, S52.202B, S52.202C, S52.209A, S52.209B, S52.209C, S52.211A, S52.212A, S52.219A, S52.221A, S52.221B, S52.221C, S52.222A, S52.222B, S52.222C, S52.223A, S52.223B, S52.223C, S52.224A, S52.224B, S52.224C, S52.225A, S52.225B, S52.225C, S52.226A, S52.226B, S52.226C, S52.231A, S52.231B, S52.231C, S52.232A, S52.232B, S52.232C, S52.233A, S52.233B, S52.233C, S52.234A, S52.234B, S52.234C, S52.235A, S52.235B, S52.235C, S52.236A, S52.236B, S52.236C, S52.241A, S52.241B, S52.241C, S52.242A, S52.242B, S52.242C, S52.243A, S52.243B, S52.243C, S52.244A, S52.244B, S52.244C, S52.245A, S52.245B, S52.245C, S52.246A, S52.246B, S52.246C, S52.251A, S52.251B, S52.251C, S52.252A, S52.252B, S52.252C, S52.253A, S52.253B, S52.253C, S52.254A, S52.254B, S52.254C, S52.255A, S52.255B, S52.255C, S52.256A, S52.256B, S52.256C, S52.261A, S52.261B, S52.261C, S52.262A, S52.262B, S52.262C, S52.263A, S52.263B, S52.263C, S52.264A, S52.264B, S52.264C, S52.265A, S52.265B, S52.265C, S52.266A, S52.266B, S52.266C, S52.271A, S52.271B, S52.271C, S52.272A, S52.272B, S52.272C, S52.279A, S52.279B, S52.279C, S52.281A, S52.281B, S52.281C, S52.282A, S52.282B, S52.282C, S52.283A, S52.283B, S52.283C, S52.291A, S52.291B, S52.291C, S52.292A, S52.292B, S52.292C, S52.299A, S52.299B, S52.299C, S52.301A, S52.301B, S52.301C, S52.302A, S52.302B, S52.302C, S52.309A, S52.309B, S52.309C, S52.311A, S52.312A, S52.319A, S52.321A, S52.321B, S52.321C, S52.322A, S52.322B, S52.322C, S52.323A, S52.323B, S52.323C, S52.324A, S52.324B, S52.324C, S52.325A, S52.325B, S52.325C, S52.326A, S52.326B, S52.326C, S52.331A, S52.331B, S52.331C, S52.332A, S52.332B, S52.332C, S52.333A, S52.333B, S52.333C, S52.334A, S52.334B, S52.334C, S52.335A, S52.335B, S52.335C, S52.336A, S52.336B, S52.336C, S52.341A, S52.341B, S52.341C, S52.342A, S52.342B, S52.342C, S52.343A, S52.343B, S52.343C, S52.344A, S52.344B, S52.344C, S52.345A, S52.345B, S52.345C, S52.346A, S52.346B, S52.346C, S52.351A, S52.351B, S52.351C, S52.352A, S52.352B, S52.352C, S52.353A, S52.353B, S52.353C, S52.354A, S52.354B, S52.354C, S52.355A, S52.355B, S52.355C, S52.356A, S52.356B, S52.356C, S52.361A, S52.361B, S52.361C, S52.362A, S52.362B, S52.362C, S52.363A, S52.363B, S52.363C, S52.364A, S52.364B, S52.364C, S52.365A, S52.365B, S52.365C, S52.366A, S52.366B, S52.366C, S52.371A, S52.371B, S52.371C, S52.372A, S52.372B, S52.372C, S52.379A, S52.379B, S52.379C, S52.381A, S52.381B, S52.381C, S52.382A, S52.382B, S52.382C, S52.389A, S52.389B, S52.389C, S52.391A, S52.391B, S52.391C, S52.392A, S52.392B, S52.392C, S52.399A, S52.399B, S52.399C, S52.501A, S52.501B, S52.501C, S52.502A, S52.502B, S52.502C, S52.509A, S52.509B, S52.509C, S52.511A, S52.511B, S52.511C, S52.512A, S52.512B, S52.512C, S52.513A, S52.513B, S52.513C, S52.514A, S52.514B, S52.514C, S52.515A, S52.515B, S52.515C, S52.516A, S52.516B, S52.516C, S52.521A, S52.522A, S52.529A, S52.531A, S52.531B, S52.531C, S52.532A, S52.532B, S52.532C, S52.539A, S52.539B, S52.539C, S52.541A, S52.541B, S52.541C, S52.542A, S52.542B, S52.542C, S52.549A, S52.549B, S52.549C, S52.551A, S52.551B, S52.551C, S52.552A, S52.552B, S52.552C, S52.559A, S52.559B, S52.559C, S52.561A, S52.561B, S52.561C, S52.562A, S52.562B, S52.562C, S52.569A, S52.569B, S52.569C, S52.571A, S52.571B, S52.571C, S52.572A, S52.572B, S52.572C, S52.579A, S52.579B, S52.579C, S52.591A, S52.591B, S52.591C, S52.592A, S52.592B, S52.592C, S52.599A, S52.599B, S52.599C, S52.601A, S52.601B, S52.601C, S52.602A, S52.602B, S52.602C, S52.609A, S52.609B, S52.609C, S52.611A, S52.611B, S52.611C, S52.612A, S52.612B, S52.612C, S52.613A, S52.613B,

© 2025 Wolpaw BJ et al. *JAMA Network Open*.

	S52.613C, S52.614A, S52.614B, S52.614C, S52.615A, S52.615B, S52.615C, S52.616A, S52.616B, S52.616C, S52.621A, S52.622A, S52.629A, S52.691A, S52.691B, S52.691C, S52.692A, S52.692B, S52.692C, S52.699A, S52.699B, S52.699C, S52.90XA, S52.90XB, S52.90XC, S52.91XA, S52.91XB, S52.91XC, S52.92XA, S52.92XB, S52.92XC, S59.001A, S59.002A, S59.009A, S59.011A, S59.012A, S59.019A, S59.021A, S59.022A, S59.029A, S59.031A, S59.032A, S59.039A, S59.041A, S59.042A, S59.049A, S59.091A, S59.092A, S59.099A, S59.101A, S59.102A, S59.109A, S59.111A, S59.112A, S59.119A, S59.121A, S59.122A, S59.129A, S59.131A, S59.132A, S59.139A, S59.141A, S59.142A, S59.149A, S59.191A, S59.192A, S59.199A, S59.201A, S59.202A, S59.209A, S59.211A, S59.212A, S59.219A, S59.221A, S59.222A, S59.229A, S59.231A, S59.232A, S59.239A, S59.241A, S59.242A, S59.249A, S59.291A, S59.292A, S59.299A, S62.001A, S62.001B, S62.002A, S62.002B, S62.009A, S62.009B, S62.011A, S62.011B, S62.012A, S62.012B, S62.013A, S62.013B, S62.014A, S62.014B, S62.015A, S62.015B, S62.016A, S62.016B, S62.021A, S62.021B, S62.022A, S62.022B, S62.023A, S62.023B, S62.024A, S62.024B, S62.025A, S62.025B, S62.026A, S62.026B, S62.031A, S62.031B, S62.032A, S62.032B, S62.033A, S62.033B, S62.034A, S62.034B, S62.035A, S62.035B, S62.036A, S62.036B, S62.101A, S62.101B, S62.102A, S62.102B, S62.109A, S62.109B, S62.111A, S62.111B, S62.112A, S62.112B, S62.113A, S62.113B, S62.114A, S62.114B, S62.115A, S62.115B, S62.116A, S62.116B, S62.121A, S62.121B, S62.122A, S62.122B, S62.123A, S62.123B, S62.124A, S62.124B, S62.125A, S62.125B, S62.126A, S62.126B, S62.131A, S62.131B, S62.132A, S62.132B, S62.133A, S62.133B, S62.134A, S62.134B, S62.135A, S62.135B, S62.136A, S62.136B, S62.141A, S62.141B, S62.142A, S62.142B, S62.143A, S62.143B, S62.144A, S62.144B, S62.145A, S62.145B, S62.146A, S62.146B, S62.151A, S62.151B, S62.152A, S62.152B, S62.153A, S62.153B, S62.154A, S62.154B, S62.155A, S62.155B, S62.156A, S62.156B, S62.161A, S62.161B, S62.162A, S62.162B, S62.163A, S62.163B, S62.164A, S62.164B, S62.165A, S62.165B, S62.166A, S62.166B, S62.171A, S62.171B, S62.172A, S62.172B, S62.173A, S62.173B, S62.174A, S62.174B, S62.175A, S62.175B, S62.176A, S62.176B, S62.181A, S62.181B, S62.182A, S62.182B, S62.183A, S62.183B, S62.184A, S62.184B, S62.185A, S62.185B, S62.186A, S62.186B, S62.201A, S62.201B, S62.202A, S62.202B, S62.209A, S62.209B, S62.211A, S62.211B, S62.212A, S62.212B, S62.213A, S62.213B, S62.221A, S62.221B, S62.222A, S62.222B, S62.223A, S62.223B, S62.224A, S62.224B, S62.225A, S62.225B, S62.226A, S62.226B, S62.231A, S62.231B, S62.232A, S62.232B, S62.233A, S62.233B, S62.234A, S62.234B, S62.235A, S62.235B, S62.236A, S62.236B, S62.241A, S62.241B, S62.242A, S62.242B, S62.243A, S62.243B, S62.244A, S62.244B, S62.245A, S62.245B, S62.246A, S62.246B, S62.251A, S62.251B, S62.252A, S62.252B, S62.253A, S62.253B, S62.254A, S62.254B, S62.255A, S62.255B, S62.256A, S62.256B, S62.291A, S62.291B, S62.292A, S62.292B, S62.299A, S62.299B, S62.300A, S62.300B, S62.301A, S62.301B, S62.302A, S62.302B, S62.303A, S62.303B, S62.304A, S62.304B, S62.305A, S62.305B, S62.306A, S62.306B, S62.307A, S62.307B, S62.308A, S62.308B, S62.309A, S62.309B, S62.310A, S62.310B, S62.311A, S62.311B, S62.312A, S62.312B, S62.313A, S62.313B, S62.314A, S62.314B, S62.315A, S62.315B, S62.316A, S62.316B, S62.317A, S62.317B, S62.318A, S62.318B, S62.319A, S62.319B, S62.320A, S62.320B, S62.321A, S62.321B, S62.322A, S62.322B, S62.323A, S62.323B, S62.324A, S62.324B, S62.325A, S62.325B, S62.326A, S62.326B, S62.327A, S62.327B, S62.328A, S62.328B, S62.329A, S62.329B, S62.330A, S62.330B, S62.331A, S62.331B, S62.332A, S62.332B, S62.333A, S62.333B, S62.334A, S62.334B, S62.335A, S62.335B, S62.336A, S62.336B, S62.337A, S62.337B, S62.338A, S62.338B, S62.339A, S62.339B, S62.340A, S62.340B, S62.341A, S62.341B, S62.342A, S62.342B, S62.343A, S62.343B, S62.344A, S62.344B, S62.345A, S62.345B, S62.346A, S62.346B, S62.347A, S62.347B, S62.348A, S62.348B, S62.349A, S62.349B, S62.350A, S62.350B, S62.351A, S62.351B, S62.352A, S62.352B, S62.353A, S62.353B, S62.354A, S62.354B, S62.355A, S62.355B, S62.356A, S62.356B, S62.357A, S62.357B, S62.358A, S62.358B, S62.359A, S62.359B, S62.360A, S62.360B, S62.361A, S62.361B, S62.362A, S62.362B, S62.363A, S62.363B, S62.364A, S62.364B, S62.365A, S62.365B, S62.366A, S62.366B, S62.367A, S62.367B, S62.368A, S62.368B, S62.369A, S62.369B, S62.390A, S62.390B, S62.391A, S62.391B, S62.392A, S62.392B, S62.393A, S62.393B, S62.394A, S62.394B, S62.395A, S62.395B, S62.396A, S62.396B, S62.397A, S62.397B, S62.398A, S62.398B, S62.399A, S62.399B, S62.501A, S62.501B, S62.502A, S62.502B, S62.509A, S62.509B, S62.511A, S62.511B, S62.512A, S62.512B, S62.513A, S62.513B, S62.514A, S62.514B, S62.515A, S62.515B, S62.516A, S62.516B, S62.521A, S62.521B, S62.522A, S62.522B, S62.523A, S62.523B, S62.524A, S62.524B, S62.525A, S62.525B, S62.526A, S62.526B, S62.600A, S62.600B, S62.601A,

© 2025 Wolpaw BJ et al. *JAMA Network Open*.

S62.601B, S62.602A, S62.602B, S62.603A, S62.603B, S62.604A, S62.604B, S62.605A, S62.605B, S62.606A, S62.606B, S62.607A, S62.607B, S62.608A, S62.608B, S62.609A, S62.609B, S62.610A, S62.610B, S62.611A, S62.611B, S62.612A, S62.612B, S62.613A, S62.613B, S62.614A, S62.614B, S62.615A, S62.615B, S62.616A, S62.616B, S62.617A, S62.617B, S62.618A, S62.618B, S62.619A, S62.619B, S62.620A, S62.620B, S62.621A, S62.621B, S62.622A, S62.622B, S62.623A, S62.623B, S62.624A, S62.624B, S62.625A, S62.625B, S62.626A, S62.626B, S62.627A, S62.627B, S62.628A, S62.628B, S62.629A, S62.629B, S62.630A, S62.630B, S62.631A, S62.631B, S62.632A, S62.632B, S62.633A, S62.633B, S62.634A, S62.634B, S62.635A, S62.635B, S62.636A, S62.636B, S62.637A, S62.637B, S62.638A, S62.638B, S62.639A, S62.639B, S62.640A, S62.640B, S62.641A, S62.641B, S62.642A, S62.642B, S62.643A, S62.643B, S62.644A, S62.644B, S62.645A, S62.645B, S62.646A, S62.646B, S62.647A, S62.647B, S62.648A, S62.648B, S62.649A, S62.649B, S62.650A, S62.650B, S62.651A, S62.651B, S62.652A, S62.652B, S62.653A, S62.653B, S62.654A, S62.654B, S62.655A, S62.655B, S62.656A, S62.656B, S62.657A, S62.657B, S62.658A, S62.658B, S62.659A, S62.659B, S62.660A, S62.660B, S62.661A, S62.661B, S62.662A, S62.662B, S62.663A, S62.663B, S62.664A, S62.664B, S62.665A, S62.665B, S62.666A, S62.666B, S62.667A, S62.667B, S62.668A, S62.668B, S62.669A, S62.669B, S62.90XA, S62.90XB, S62.91XA, S62.91XB, S62.92XA, S62.92XB, M97.11XA, M97.12XA, M97.21XA, M97.22XA, M97.8XXA, M97.9XXA, S72.301A, S72.301B, S72.301C, S72.302A, S72.302B, S72.302C, S72.309A, S72.309B, S72.309C, S72.321A, S72.321B, S72.321C, S72.322A, S72.322B, S72.322C, S72.323A, S72.323B, S72.323C, S72.324A, S72.324B, S72.324C, S72.325A, S72.325B, S72.325C, S72.326A, S72.326B, S72.326C, S72.331A, S72.331B, S72.331C, S72.332A, S72.332B, S72.332C, S72.333A, S72.333B, S72.333C, S72.334A, S72.334B, S72.334C, S72.335A, S72.335B, S72.335C, S72.336A, S72.336B, S72.336C, S72.341A, S72.341B, S72.341C, S72.342A, S72.342B, S72.342C, S72.343A, S72.343B, S72.343C, S72.344A, S72.344B, S72.344C, S72.345A, S72.345B, S72.345C, S72.346A, S72.346B, S72.346C, S72.351A, S72.351B, S72.351C, S72.352A, S72.352B, S72.352C, S72.353A, S72.353B, S72.353C, S72.354A, S72.354B, S72.354C, S72.355A, S72.355B, S72.355C, S72.356A, S72.356B, S72.356C, S72.361A, S72.361B, S72.361C, S72.362A, S72.362B, S72.362C, S72.363A, S72.363B, S72.363C, S72.364A, S72.364B, S72.364C, S72.365A, S72.365B, S72.365C, S72.366A, S72.366B, S72.366C, S72.391A, S72.391B, S72.391C, S72.392A, S72.392B, S72.392C, S72.399A, S72.399B, S72.399C, S72.401A, S72.401B, S72.401C, S72.402A, S72.402B, S72.402C, S72.409A, S72.409B, S72.409C, S72.411A, S72.411B, S72.411C, S72.412A, S72.412B, S72.412C, S72.413A, S72.413B, S72.413C, S72.414A, S72.414B, S72.414C, S72.415A, S72.415B, S72.415C, S72.416A, S72.416B, S72.416C, S72.421A, S72.421B, S72.421C, S72.422A, S72.422B, S72.422C, S72.423A, S72.423B, S72.423C, S72.424A, S72.424B, S72.424C, S72.425A, S72.425B, S72.425C, S72.426A, S72.426B, S72.426C, S72.431A, S72.431B, S72.431C, S72.432A, S72.432B, S72.432C, S72.433A, S72.433B, S72.433C, S72.434A, S72.434B, S72.434C, S72.435A, S72.435B, S72.435C, S72.436A, S72.436B, S72.436C, S72.441A, S72.441B, S72.441C, S72.442A, S72.442B, S72.442C, S72.443A, S72.443B, S72.443C, S72.444A, S72.444B, S72.444C, S72.445A, S72.445B, S72.445C, S72.446A, S72.446B, S72.446C, S72.451A, S72.451B, S72.451C, S72.452A, S72.452B, S72.452C, S72.453A, S72.453B, S72.453C, S72.454A, S72.454B, S72.454C, S72.455A, S72.455B, S72.455C, S72.456A, S72.456B, S72.456C, S72.461A, S72.461B, S72.461C, S72.462A, S72.462B, S72.462C, S72.463A, S72.463B, S72.463C, S72.464A, S72.464B, S72.464C, S72.465A, S72.465B, S72.465C, S72.466A, S72.466B, S72.466C, S72.471A, S72.472A, S72.479A, S72.491A, S72.491B, S72.491C, S72.492A, S72.492B, S72.492C, S72.499A, S72.499B, S72.499C, S72.8X1A, S72.8X1B, S72.8X1C, S72.8X2A, S72.8X2B, S72.8X2C, S72.8X9A, S72.8X9B, S72.8X9C, S72.90XA, S72.90XB, S72.90XC, S72.91XA, S72.91XB, S72.91XC, S72.92XA, S72.92XB, S72.92XC, S79.001A, S79.002A, S79.009A, S79.011A, S79.012A, S79.019A, S79.091A, S79.092A, S79.099A, S79.101A, S79.102A, S79.109A, S79.111A, S79.112A, S79.119A, S79.121A, S79.122A, S79.129A, S79.131A, S79.132A, S79.139A, S79.141A, S79.142A, S79.149A, S79.191A, S79.192A, S79.199A, S82.001A, S82.001B, S82.001C, S82.002A, S82.002B, S82.002C, S82.009A, S82.009B, S82.009C, S82.011A, S82.011B, S82.011C, S82.012A, S82.012B, S82.012C, S82.013A, S82.013B, S82.013C, S82.014A, S82.014B, S82.014C, S82.015A, S82.015B, S82.015C, S82.016A, S82.016B, S82.016C, S82.021A, S82.021B, S82.021C, S82.022A, S82.022B, S82.022C, S82.023A, S82.023B, S82.023C, S82.024A, S82.024B, S82.024C, S82.025A, S82.025B, S82.025C, S82.026A, S82.026B, S82.026C, S82.031A, S82.031B, S82.031C, S82.032A, S82.032B, S82.032C, S82.033A, S82.033B, S82.033C, S82.034A, S82.034B, S82.034C,

© 2025 Wolpaw BJ et al. *JAMA Network Open*.

S82.035A, S82.035B, S82.035C, S82.036A, S82.036B, S82.036C, S82.041A, S82.041B, S82.041C, S82.042A, S82.042B, S82.042C, S82.043A, S82.043B, S82.043C, S82.044A, S82.044B, S82.044C, S82.045A, S82.045B, S82.045C, S82.046A, S82.046B, S82.046C, S82.091A, S82.091B, S82.091C, S82.092A, S82.092B, S82.092C, S82.099A, S82.099B, S82.099C, S82.101A, S82.101B, S82.101C, S82.102A, S82.102B, S82.102C, S82.109A, S82.109B, S82.109C, S82.111A, S82.111B, S82.111C, S82.112A, S82.112B, S82.112C, S82.113A, S82.113B, S82.113C, S82.114A, S82.114B, S82.114C, S82.115A, S82.115B, S82.115C, S82.116A, S82.116B, S82.116C, S82.121A, S82.121B, S82.121C, S82.122A, S82.122B, S82.122C, S82.123A, S82.123B, S82.123C, S82.124A, S82.124B, S82.124C, S82.125A, S82.125B, S82.125C, S82.126A, S82.126B, S82.126C, S82.131A, S82.131B, S82.131C, S82.132A, S82.132B, S82.132C, S82.133A, S82.133B, S82.133C, S82.134A, S82.134B, S82.134C, S82.135A, S82.135B, S82.135C, S82.136A, S82.136B, S82.136C, S82.141A, S82.141B, S82.141C, S82.142A, S82.142B, S82.142C, S82.143A, S82.143B, S82.143C, S82.144A, S82.144B, S82.144C, S82.145A, S82.145B, S82.145C, S82.146A, S82.146B, S82.146C, S82.151A, S82.151B, S82.151C, S82.152A, S82.152B, S82.152C, S82.153A, S82.153B, S82.153C, S82.154A, S82.154B, S82.154C, S82.155A, S82.155B, S82.155C, S82.156A, S82.156B, S82.156C, S82.161A, S82.162A, S82.169A, S82.191A, S82.191B, S82.191C, S82.192A, S82.192B, S82.192C, S82.199A, S82.199B, S82.199C, S82.201A, S82.201B, S82.201C, S82.202A, S82.202B, S82.202C, S82.209A, S82.209B, S82.209C, S82.221A, S82.221B, S82.221C, S82.222A, S82.222B, S82.222C, S82.223A, S82.223B, S82.223C, S82.224A, S82.224B, S82.224C, S82.225A, S82.225B, S82.225C, S82.226A, S82.226B, S82.226C, S82.231A, S82.231B, S82.231C, S82.232A, S82.232B, S82.232C, S82.233A, S82.233B, S82.233C, S82.234A, S82.234B, S82.234C, S82.235A, S82.235B, S82.235C, S82.236A, S82.236B, S82.236C, S82.241A, S82.241B, S82.241C, S82.242A, S82.242B, S82.242C, S82.243A, S82.243B, S82.243C, S82.244A, S82.244B, S82.244C, S82.245A, S82.245B, S82.245C, S82.246A, S82.246B, S82.246C, S82.251A, S82.251B, S82.251C, S82.252A, S82.252B, S82.252C, S82.253A, S82.253B, S82.253C, S82.254A, S82.254B, S82.254C, S82.255A, S82.255B, S82.255C, S82.256A, S82.256B, S82.256C, S82.261A, S82.261B, S82.261C, S82.262A, S82.262B, S82.262C, S82.263A, S82.263B, S82.263C, S82.264A, S82.264B, S82.264C, S82.265A, S82.265B, S82.265C, S82.266A, S82.266B, S82.266C, S82.291A, S82.291B, S82.291C, S82.292A, S82.292B, S82.292C, S82.299A, S82.299B, S82.299C, S82.301A, S82.301B, S82.301C, S82.302A, S82.302B, S82.302C, S82.309A, S82.309B, S82.309C, S82.311A, S82.312A, S82.319A, S82.391A, S82.391B, S82.391C, S82.392A, S82.392B, S82.392C, S82.399A, S82.399B, S82.399C, S82.401A, S82.401B, S82.401C, S82.402A, S82.402B, S82.402C, S82.409A, S82.409B, S82.409C, S82.421A, S82.421B, S82.421C, S82.422A, S82.422B, S82.422C, S82.423A, S82.423B, S82.423C, S82.424A, S82.424B, S82.424C, S82.425A, S82.425B, S82.425C, S82.426A, S82.426B, S82.426C, S82.431A, S82.431B, S82.431C, S82.432A, S82.432B, S82.432C, S82.433A, S82.433B, S82.433C, S82.434A, S82.434B, S82.434C, S82.435A, S82.435B, S82.435C, S82.436A, S82.436B, S82.436C, S82.441A, S82.441B, S82.441C, S82.442A, S82.442B, S82.442C, S82.443A, S82.443B, S82.443C, S82.444A, S82.444B, S82.444C, S82.445A, S82.445B, S82.445C, S82.446A, S82.446B, S82.446C, S82.451A, S82.451B, S82.451C, S82.452A, S82.452B, S82.452C, S82.453A, S82.453B, S82.453C, S82.454A, S82.454B, S82.454C, S82.455A, S82.455B, S82.455C, S82.456A, S82.456B, S82.456C, S82.461A, S82.461B, S82.461C, S82.462A, S82.462B, S82.462C, S82.463A, S82.463B, S82.463C, S82.464A, S82.464B, S82.464C, S82.465A, S82.465B, S82.465C, S82.466A, S82.466B, S82.466C, S82.491A, S82.491B, S82.491C, S82.492A, S82.492B, S82.492C, S82.499A, S82.499B, S82.499C, S82.51XA, S82.51XB, S82.51XC, S82.52XA, S82.52XB, S82.52XC, S82.53XA, S82.53XB, S82.53XC, S82.54XA, S82.54XB, S82.54XC, S82.55XA, S82.55XB, S82.55XC, S82.56XA, S82.56XB, S82.56XC, S82.61XA, S82.61XB, S82.61XC, S82.62XA, S82.62XB, S82.62XC, S82.63XA, S82.63XB, S82.63XC, S82.64XA, S82.64XB, S82.64XC, S82.65XA, S82.65XB, S82.65XC, S82.66XA, S82.66XB, S82.66XC, S82.811A, S82.812A, S82.819A, S82.821A, S82.822A, S82.829A, S82.831A, S82.831B, S82.831C, S82.832A, S82.832B, S82.832C, S82.839A, S82.839B, S82.839C, S82.841A, S82.841B, S82.841C, S82.842A, S82.842B, S82.842C, S82.843A, S82.843B, S82.843C, S82.844A, S82.844B, S82.844C, S82.845A, S82.845B, S82.845C, S82.846A, S82.846B, S82.846C, S82.851A, S82.851B, S82.851C, S82.852A, S82.852B, S82.852C, S82.853A, S82.853B, S82.853C, S82.854A, S82.854B, S82.854C, S82.855A, S82.855B, S82.855C, S82.856A, S82.856B, S82.856C, S82.861A, S82.861B, S82.861C, S82.862A, S82.862B, S82.862C, S82.863A, S82.863B, S82.863C, S82.864A, S82.864B, S82.864C, S82.865A, S82.865B, S82.865C, S82.866A, S82.866B, S82.866C, S82.871A, S82.871B,

© 2025 Wolpaw BJ et al. *JAMA Network Open*.

S82.871C, S82.872A, S82.872B, S82.872C, S82.873A, S82.873B, S82.873C, S82.874A, S82.874B, S82.874C, S82.875A, S82.875B, S82.875C, S82.876A, S82.876B, S82.876C, S82.891A, S82.891B, S82.891C, S82.892A, S82.892B, S82.892C, S82.899A, S82.899B, S82.899C, S82.90XA, S82.90XB, S82.90XC, S82.91XA, S82.91XB, S82.91XC, S82.92XA, S82.92XB, S82.92XC, S89.001A, S89.002A, S89.009A, S89.011A, S89.012A, S89.019A, S89.021A, S89.022A, S89.029A, S89.031A, S89.032A, S89.039A, S89.041A, S89.042A, S89.049A, S89.091A, S89.092A, S89.099A, S89.101A, S89.102A, S89.109A, S89.111A, S89.112A, S89.119A, S89.121A, S89.122A, S89.129A, S89.131A, S89.132A, S89.139A, S89.141A, S89.142A, S89.149A, S89.191A, S89.192A, S89.199A, S89.201A, S89.202A, S89.209A, S89.211A, S89.212A, S89.219A, S89.221A, S89.222A, S89.229A, S89.291A, S89.292A, S89.299A, S89.301A, S89.302A, S89.309A, S89.311A, S89.312A, S89.319A, S89.321A, S89.322A, S89.329A, S89.391A, S89.392A, S89.399A, S92.001A, S92.001B, S92.002A, S92.002B, S92.009A, S92.009B, S92.011A, S92.011B, S92.012A, S92.012B, S92.013A, S92.013B, S92.014A, S92.014B, S92.015A, S92.015B, S92.016A, S92.016B, S92.021A, S92.021B, S92.022A, S92.022B, S92.023A, S92.023B, S92.024A, S92.024B, S92.025A, S92.025B, S92.026A, S92.026B, S92.031A, S92.031B, S92.032A, S92.032B, S92.033A, S92.033B, S92.034A, S92.034B, S92.035A, S92.035B, S92.036A, S92.036B, S92.041A, S92.041B, S92.042A, S92.042B, S92.043A, S92.043B, S92.044A, S92.044B, S92.045A, S92.045B, S92.046A, S92.046B, S92.051A, S92.051B, S92.052A, S92.052B, S92.053A, S92.053B, S92.054A, S92.054B, S92.055A, S92.055B, S92.056A, S92.056B, S92.061A, S92.061B, S92.062A, S92.062B, S92.063A, S92.063B, S92.064A, S92.064B, S92.065A, S92.065B, S92.066A, S92.066B, S92.101A, S92.101B, S92.102A, S92.102B, S92.109A, S92.109B, S92.111A, S92.111B, S92.112A, S92.112B, S92.113A, S92.113B, S92.114A, S92.114B, S92.115A, S92.115B, S92.116A, S92.116B, S92.121A, S92.121B, S92.122A, S92.122B, S92.123A, S92.123B, S92.124A, S92.124B, S92.125A, S92.125B, S92.126A, S92.126B, S92.131A, S92.131B, S92.132A, S92.132B, S92.133A, S92.133B, S92.134A, S92.134B, S92.135A, S92.135B, S92.136A, S92.136B, S92.141A, S92.141B, S92.142A, S92.142B, S92.143A, S92.143B, S92.144A, S92.144B, S92.145A, S92.145B, S92.146A, S92.146B, S92.151A, S92.151B, S92.152A, S92.152B, S92.153A, S92.153B, S92.154A, S92.154B, S92.155A, S92.155B, S92.156A, S92.156B, S92.191A, S92.191B, S92.192A, S92.192B, S92.199A, S92.199B, S92.201A, S92.201B, S92.202A, S92.202B, S92.209A, S92.209B, S92.211A, S92.211B, S92.212A, S92.212B, S92.213A, S92.213B, S92.214A, S92.214B, S92.215A, S92.215B, S92.216A, S92.216B, S92.221A, S92.221B, S92.222A, S92.222B, S92.223A, S92.223B, S92.224A, S92.224B, S92.225A, S92.225B, S92.226A, S92.226B, S92.231A, S92.231B, S92.232A, S92.232B, S92.233A, S92.233B, S92.234A, S92.234B, S92.235A, S92.235B, S92.236A, S92.236B, S92.241A, S92.241B, S92.242A, S92.242B, S92.243A, S92.243B, S92.244A, S92.244B, S92.245A, S92.245B, S92.246A, S92.246B, S92.251A, S92.251B, S92.252A, S92.252B, S92.253A, S92.253B, S92.254A, S92.254B, S92.255A, S92.255B, S92.256A, S92.256B, S92.301A, S92.301B, S92.302A, S92.302B, S92.309A, S92.309B, S92.311A, S92.311B, S92.312A, S92.312B, S92.313A, S92.313B, S92.314A, S92.314B, S92.315A, S92.315B, S92.316A, S92.316B, S92.321A, S92.321B, S92.322A, S92.322B, S92.323A, S92.323B, S92.324A, S92.324B, S92.325A, S92.325B, S92.326A, S92.326B, S92.331A, S92.331B, S92.332A, S92.332B, S92.333A, S92.333B, S92.334A, S92.334B, S92.335A, S92.335B, S92.336A, S92.336B, S92.341A, S92.341B, S92.342A, S92.342B, S92.343A, S92.343B, S92.344A, S92.344B, S92.345A, S92.345B, S92.346A, S92.346B, S92.351A, S92.351B, S92.352A, S92.352B, S92.353A, S92.353B, S92.354A, S92.354B, S92.355A, S92.355B, S92.356A, S92.356B, S92.401A, S92.401B, S92.402A, S92.402B, S92.403A, S92.403B, S92.404A, S92.404B, S92.405A, S92.405B, S92.406A, S92.406B, S92.411A, S92.411B, S92.412A, S92.412B, S92.413A, S92.413B, S92.414A, S92.414B, S92.415A, S92.415B, S92.416A, S92.416B, S92.421A, S92.421B, S92.422A, S92.422B, S92.423A, S92.423B, S92.424A, S92.424B, S92.425A, S92.425B, S92.426A, S92.426B, S92.491A, S92.491B, S92.492A, S92.492B, S92.499A, S92.499B, S92.501A, S92.501B, S92.502A, S92.502B, S92.503A, S92.503B, S92.504A, S92.504B, S92.505A, S92.505B, S92.506A, S92.506B, S92.511A, S92.511B, S92.512A, S92.512B, S92.513A, S92.513B, S92.514A, S92.514B, S92.515A, S92.515B, S92.516A, S92.516B, S92.521A, S92.521B, S92.522A, S92.522B, S92.523A, S92.523B, S92.524A, S92.524B, S92.525A, S92.525B, S92.526A, S92.526B, S92.531A, S92.531B, S92.532A, S92.532B, S92.533A, S92.533B, S92.534A, S92.534B, S92.535A, S92.535B, S92.536A, S92.536B, S92.591A, S92.591B, S92.592A, S92.592B, S92.599A, S92.599B, S92.811A, S92.811B, S92.812A, S92.812B, S92.819A, S92.819B, S92.901A, S92.901B, S92.902A, S92.902B, S92.909A, S92.909B, S92.911A, S92.911B, S92.912A, S92.912B,

© 2025 Wolpaw BJ et al. *JAMA Network Open*.

S92.919A, S92.919B, S99.001A, S99.001B, S99.002A, S99.002B, S99.009A, S99.009B, S99.011A, S99.011B, S99.012A, S99.012B, S99.019A, S99.019B, S99.021A, S99.021B, S99.022A, S99.022B, S99.029A, S99.029B, S99.031A, S99.031B, S99.032A, S99.032B, S99.039A, S99.039B, S99.041A, S99.041B, S99.042A, S99.042B, S99.049A, S99.049B, S99.091A, S99.091B, S99.092A, S99.092B, S99.099A, S99.099B, S99.101A, S99.101B, S99.102A, S99.102B, S99.109A, S99.109B, S99.111A, S99.111B, S99.112A, S99.112B, S99.119A, S99.119B, S99.121A, S99.121B, S99.122A, S99.122B, S99.129A, S99.129B, S99.131A, S99.131B, S99.132A, S99.132B, S99.139A, S99.139B, S99.141A, S99.141B, S99.142A, S99.142B, S99.149A, S99.149B, S99.191A, S99.191B, S99.192A, S99.192B, S99.199A, S99.199B, S99.201A, S99.201B, S99.202A, S99.202B, S99.209A, S99.209B, S99.211A, S99.211B, S99.212A, S99.212B, S99.219A, S99.219B, S99.221A, S99.221B, S99.222A, S99.222B, S99.229A, S99.229B, S99.231A, S99.231B, S99.232A, S99.232B, S99.239A, S99.239B, S99.241A, S99.241B, S99.242A, S99.242B, S99.249A, S99.249B, S99.291A, S99.291B, S99.292A, S99.292B, S99.299A, S99.299B, T84.042A, T84.043A, T84.048A, T84.049A, M97.01XA, M97.02XA, S72.001A, S72.001B, S72.001C, S72.002A, S72.002B, S72.002C, S72.009A, S72.009B, S72.009C, S72.011A, S72.011B, S72.011C, S72.012A, S72.012B, S72.012C, S72.019A, S72.019B, S72.019C, S72.021A, S72.021B, S72.021C, S72.022A, S72.022B, S72.022C, S72.023A, S72.023B, S72.023C, S72.024A, S72.024B, S72.024C, S72.025A, S72.025B, S72.025C, S72.026A, S72.026B, S72.026C, S72.031A, S72.031B, S72.031C, S72.032A, S72.032B, S72.032C, S72.033A, S72.033B, S72.033C, S72.034A, S72.034B, S72.034C, S72.035A, S72.035B, S72.035C, S72.036A, S72.036B, S72.036C, S72.041A, S72.041B, S72.041C, S72.042A, S72.042B, S72.042C, S72.043A, S72.043B, S72.043C, S72.044A, S72.044B, S72.044C, S72.045A, S72.045B, S72.045C, S72.046A, S72.046B, S72.046C, S72.051A, S72.051B, S72.051C, S72.052A, S72.052B, S72.052C, S72.059A, S72.059B, S72.059C, S72.061A, S72.061B, S72.061C, S72.062A, S72.062B, S72.062C, S72.063A, S72.063B, S72.063C, S72.064A, S72.064B, S72.064C, S72.065A, S72.065B, S72.065C, S72.066A, S72.066B, S72.066C, S72.091A, S72.091B, S72.091C, S72.092A, S72.092B, S72.092C, S72.099A, S72.099B, S72.099C, S72.101A, S72.101B, S72.101C, S72.102A, S72.102B, S72.102C, S72.109A, S72.109B, S72.109C, S72.111A, S72.111B, S72.111C, S72.112A, S72.112B, S72.112C, S72.113A, S72.113B, S72.113C, S72.114A, S72.114B, S72.114C, S72.115A, S72.115B, S72.115C, S72.116A, S72.116B, S72.116C, S72.121A, S72.121B, S72.121C, S72.122A, S72.122B, S72.122C, S72.123A, S72.123B, S72.123C, S72.124A, S72.124B, S72.124C, S72.125A, S72.125B, S72.125C, S72.126A, S72.126B, S72.126C, S72.131A, S72.131B, S72.131C, S72.132A, S72.132B, S72.132C, S72.133A, S72.133B, S72.133C, S72.134A, S72.134B, S72.134C, S72.135A, S72.135B, S72.135C, S72.136A, S72.136B, S72.136C, S72.141A, S72.141B, S72.141C, S72.142A, S72.142B, S72.142C, S72.143A, S72.143B, S72.143C, S72.144A, S72.144B, S72.144C, S72.145A, S72.145B, S72.145C, S72.146A, S72.146B, S72.146C, S72.21XA, S72.21XB, S72.21XC, S72.22XA, S72.22XB, S72.22XC, S72.23XA, S72.23XB, S72.23XC, S72.24XA, S72.24XB, S72.24XC, S72.25XA, S72.25XB, S72.25XC, S72.26XA, S72.26XB, S72.26XC, T84.040A, T84.041A, S03.00XA, S03.01XA, S03.02XA, S03.03XA, S03.0XXA, S03.1XXA, S03.2XXA, S13.0XXA, S13.100A, S13.101A, S13.110A, S13.111A, S13.120A, S13.121A, S13.130A, S13.131A, S13.140A, S13.141A, S13.150A, S13.151A, S13.160A, S13.161A, S13.170A, S13.171A, S13.180A, S13.181A, S13.20XA, S13.29XA, S23.0XXA, S23.100A, S23.101A, S23.110A, S23.111A, S23.120A, S23.121A, S23.122A, S23.123A, S23.130A, S23.131A, S23.132A, S23.133A, S23.140A, S23.141A, S23.142A, S23.143A, S23.150A, S23.151A, S23.152A, S23.153A, S23.160A, S23.161A, S23.162A, S23.163A, S23.170A, S23.171A, S23.20XA, S23.29XA, S33.0XXA, S33.100A, S33.101A, S33.110A, S33.111A, S33.120A, S33.121A, S33.130A, S33.131A, S33.140A, S33.141A, S33.2XXA, S33.30XA, S33.39XA, S33.4XXA, S43.001A, S43.002A, S43.003A, S43.004A, S43.005A, S43.006A, S43.011A, S43.012A, S43.013A, S43.014A, S43.015A, S43.016A, S43.021A, S43.022A, S43.023A, S43.024A, S43.025A, S43.026A, S43.031A, S43.032A, S43.033A, S43.034A, S43.035A, S43.036A, S43.081A, S43.082A, S43.083A, S43.084A, S43.085A, S43.086A, S43.101A, S43.102A, S43.109A, S43.111A, S43.112A, S43.119A, S43.121A, S43.122A, S43.129A, S43.131A, S43.132A, S43.139A, S43.141A, S43.142A, S43.149A, S43.151A, S43.152A, S43.159A, S43.201A, S43.202A, S43.203A, S43.204A, S43.205A, S43.206A, S43.211A, S43.212A, S43.213A, S43.214A, S43.215A, S43.216A, S43.221A, S43.222A, S43.223A, S43.224A, S43.225A, S43.226A, S43.301A, S43.302A, S43.303A, S43.304A, S43.305A, S43.306A, S43.311A, S43.312A, S43.313A, S43.314A, S43.315A, S43.316A, S43.391A, S43.392A, S43.393A, S43.394A, S43.395A, S43.396A, S53.001A, S53.002A, S53.003A, S53.004A, S53.005A, S53.006A, S53.011A, S53.012A,

© 2025 Wolpaw BJ et al. *JAMA Network Open*.

S53.013A, S53.014A, S53.015A, S53.016A, S53.021A, S53.022A, S53.023A, S53.024A, S53.025A, S53.026A, S53.031A, S53.032A, S53.033A, S53.091A, S53.092A, S53.093A, S53.094A, S53.095A, S53.096A, S53.101A, S53.102A, S53.103A, S53.104A, S53.105A, S53.106A, S53.111A, S53.112A, S53.113A, S53.114A, S53.115A, S53.116A, S53.121A, S53.122A, S53.123A, S53.124A, S53.125A, S53.126A, S53.131A, S53.132A, S53.133A, S53.134A, S53.135A, S53.136A, S53.141A, S53.142A, S53.143A, S53.144A, S53.145A, S53.146A, S53.191A, S53.192A, S53.193A, S53.194A, S53.195A, S53.196A, S63.001A, S63.002A, S63.003A, S63.004A, S63.005A, S63.006A, S63.011A, S63.012A, S63.013A, S63.014A, S63.015A, S63.016A, S63.021A, S63.022A, S63.023A, S63.024A, S63.025A, S63.026A, S63.031A, S63.032A, S63.033A, S63.034A, S63.035A, S63.036A, S63.041A, S63.042A, S63.043A, S63.044A, S63.045A, S63.046A, S63.051A, S63.052A, S63.053A, S63.054A, S63.055A, S63.056A, S63.061A, S63.062A, S63.063A, S63.064A, S63.065A, S63.066A, S63.071A, S63.072A, S63.073A, S63.074A, S63.075A, S63.076A, S63.091A, S63.092A, S63.093A, S63.094A, S63.095A, S63.096A, S63.101A, S63.102A, S63.103A, S63.104A, S63.105A, S63.106A, S63.111A, S63.112A, S63.113A, S63.114A, S63.115A, S63.116A, S63.121A, S63.122A, S63.123A, S63.124A, S63.125A, S63.126A, S63.131A, S63.132A, S63.133A, S63.134A, S63.135A, S63.136A, S63.141A, S63.142A, S63.143A, S63.144A, S63.145A, S63.146A, S63.200A, S63.201A, S63.202A, S63.203A, S63.204A, S63.205A, S63.206A, S63.207A, S63.208A, S63.209A, S63.210A, S63.211A, S63.212A, S63.213A, S63.214A, S63.215A, S63.216A, S63.217A, S63.218A, S63.219A, S63.220A, S63.221A, S63.222A, S63.223A, S63.224A, S63.225A, S63.226A, S63.227A, S63.228A, S63.229A, S63.230A, S63.231A, S63.232A, S63.233A, S63.234A, S63.235A, S63.236A, S63.237A, S63.238A, S63.239A, S63.240A, S63.241A, S63.242A, S63.243A, S63.244A, S63.245A, S63.246A, S63.247A, S63.248A, S63.249A, S63.250A, S63.251A, S63.252A, S63.253A, S63.254A, S63.255A, S63.256A, S63.257A, S63.258A, S63.259A, S63.260A, S63.261A, S63.262A, S63.263A, S63.264A, S63.265A, S63.266A, S63.267A, S63.268A, S63.269A, S63.270A, S63.271A, S63.272A, S63.273A, S63.274A, S63.275A, S63.276A, S63.277A, S63.278A, S63.279A, S63.280A, S63.281A, S63.282A, S63.283A, S63.284A, S63.285A, S63.286A, S63.287A, S63.288A, S63.289A, S63.290A, S63.291A, S63.292A, S63.293A, S63.294A, S63.295A, S63.296A, S63.297A, S63.298A, S63.299A, S73.001A, S73.002A, S73.003A, S73.004A, S73.005A, S73.006A, S73.011A, S73.012A, S73.013A, S73.014A, S73.015A, S73.016A, S73.021A, S73.022A, S73.023A, S73.024A, S73.025A, S73.026A, S73.031A, S73.032A, S73.033A, S73.034A, S73.035A, S73.036A, S73.041A, S73.042A, S73.043A, S73.044A, S73.045A, S73.046A, S83.001A, S83.002A, S83.003A, S83.004A, S83.005A, S83.006A, S83.011A, S83.012A, S83.013A, S83.014A, S83.015A, S83.016A, S83.091A, S83.092A, S83.093A, S83.094A, S83.095A, S83.096A, S83.101A, S83.102A, S83.103A, S83.104A, S83.105A, S83.106A, S83.111A, S83.112A, S83.113A, S83.114A, S83.115A, S83.116A, S83.121A, S83.122A, S83.123A, S83.124A, S83.125A, S83.126A, S83.131A, S83.132A, S83.133A, S83.134A, S83.135A, S83.136A, S83.141A, S83.142A, S83.143A, S83.144A, S83.145A, S83.146A, S83.191A, S83.192A, S83.193A, S83.194A, S83.195A, S83.196A, S93.01XA, S93.02XA, S93.03XA, S93.04XA, S93.05XA, S93.06XA, S93.101A, S93.102A, S93.103A, S93.104A, S93.105A, S93.106A, S93.111A, S93.112A, S93.113A, S93.114A, S93.115A, S93.116A, S93.119A, S93.121A, S93.122A, S93.123A, S93.124A, S93.125A, S93.126A, S93.129A, S93.131A, S93.132A, S93.133A, S93.134A, S93.135A, S93.136A, S93.139A, S93.141A, S93.142A, S93.143A, S93.144A, S93.145A, S93.146A, S93.149A, S93.301A, S93.302A, S93.303A, S93.304A, S93.305A, S93.306A, S93.311A, S93.312A, S93.313A, S93.314A, S93.315A, S93.316A, S93.321A, S93.322A, S93.323A, S93.324A, S93.325A, S93.326A, S93.331A, S93.332A, S93.333A, S93.334A, S93.335A, S93.336A, S14.0XXA, S14.101A, S14.102A, S14.103A, S14.104A, S14.105A, S14.106A, S14.107A, S14.108A, S14.109A, S14.111A, S14.112A, S14.113A, S14.114A, S14.115A, S14.116A, S14.117A, S14.118A, S14.119A, S14.121A, S14.122A, S14.123A, S14.124A, S14.125A, S14.126A, S14.127A, S14.128A, S14.129A, S14.131A, S14.132A, S14.133A, S14.134A, S14.135A, S14.136A, S14.137A, S14.138A, S14.139A, S14.141A, S14.142A, S14.143A, S14.144A, S14.145A, S14.146A, S14.147A, S14.148A, S14.149A, S14.151A, S14.152A, S14.153A, S14.154A, S14.155A, S14.156A, S14.157A, S14.158A, S14.159A, S24.0XXA, S24.101A, S24.102A, S24.103A, S24.104A, S24.109A, S24.111A, S24.112A, S24.113A, S24.114A, S24.119A, S24.131A, S24.132A, S24.133A, S24.134A, S24.139A, S24.141A, S24.142A, S24.143A, S24.144A, S24.149A, S24.151A, S24.152A, S24.153A, S24.154A, S24.159A, S34.01XA, S34.02XA, S34.101A, S34.102A, S34.103A, S34.104A, S34.105A, S34.109A, S34.111A, S34.112A, S34.113A, S34.114A, S34.115A, S34.119A, S34.121A, S34.122A, S34.123A, S34.124A, S34.125A, S34.129A,

© 2025 Wolpaw BJ et al. *JAMA Network Open*.

S34.131A, S34.132A, S34.139A, S34.3XXA, T33.011A, T33.012A, T33.019A, T33.02XA, T33.09XA, T33.1XXA, T33.2XXA, T33.3XXA, T33.40XA, T33.41XA, T33.42XA, T33.511A, T33.512A, T33.519A, T33.521A, T33.522A, T33.529A, T33.531A, T33.532A, T33.539A, T33.60XA, T33.61XA, T33.62XA, T33.70XA, T33.71XA, T33.72XA, T33.811A, T33.812A, T33.819A, T33.821A, T33.822A, T33.829A, T33.831A, T33.832A, T33.839A, T33.90XA, T33.99XA, T34.011A, T34.012A, T34.019A, T34.02XA, T34.09XA, T34.1XXA, T34.2XXA, T34.3XXA, T34.40XA, T34.41XA, T34.42XA, T34.511A, T34.512A, T34.519A, T34.521A, T34.522A, T34.529A, T34.531A, T34.532A, T34.539A, T34.60XA, T34.61XA, T34.62XA, T34.70XA, T34.71XA, T34.72XA, T34.811A, T34.812A, T34.819A, T34.821A, T34.822A, T34.829A, T34.831A, T34.832A, T34.839A, T34.90XA, T34.99XA, T66.XXXA, T67.01XA, T67.02XA, T67.09XA, T67.0XXA, T67.1XXA, T67.2XXA, T67.3XXA, T67.4XXA, T67.5XXA, T67.6XXA, T67.7XXA, T67.8XXA, T67.9XXA, T68.XXXA, T69.011A, T69.012A, T69.019A, T69.021A, T69.022A, T69.029A, T69.1XXA, T69.8XXA, T69.9XXA, T70.0XXA, T70.1XXA, T70.20XA, T70.29XA, T70.3XXA, T70.4XXA, T70.8XXA, T70.9XXA, T71.111A, T71.112A, T71.113A, T71.114A, T71.121A, T71.122A, T71.123A, T71.124A, T71.131A, T71.132A, T71.133A, T71.134A, T71.141A, T71.143A, T71.144A, T71.151A, T71.152A, T71.153A, T71.154A, T71.161A, T71.162A, T71.163A, T71.164A, T71.191A, T71.192A, T71.193A, T71.194A, T71.20XA, T71.21XA, T71.221A, T71.222A, T71.223A, T71.224A, T71.231A, T71.232A, T71.233A, T71.234A, T71.29XA, T71.9XXA, T73.0XXA, T73.1XXA, T73.2XXA, T73.3XXA, T73.8XXA, T73.9XXA, T75.00XA, T75.01XA, T75.09XA, T75.1XXA, T75.20XA, T75.21XA, T75.22XA, T75.23XA, T75.29XA, T75.3XXA, T75.4XXA, T75.81XA, T75.82XA, T75.89XA, S03.40XA, S03.41XA, S03.42XA, S03.43XA, S03.4XXA, S03.8XXA, S03.9XXA, S09.11XA, S13.4XXA, S13.5XXA, S13.8XXA, S13.9XXA, S16.1XXA, S23.3XXA, S23.41XA, S23.420A, S23.421A, S23.428A, S23.429A, S23.8XXA, S23.9XXA, S29.011A, S29.012A, S29.019A, S33.5XXA, S33.6XXA, S33.8XXA, S33.9XXA, S39.011A, S39.012A, S39.013A, S43.401A, S43.402A, S43.409A, S43.411A, S43.412A, S43.419A, S43.421A, S43.422A, S43.429A, S43.431A, S43.432A, S43.439A, S43.491A, S43.492A, S43.499A, S43.50XA, S43.51XA, S43.52XA, S43.60XA, S43.61XA, S43.62XA, S43.80XA, S43.81XA, S43.82XA, S43.90XA, S43.91XA, S43.92XA, S46.011A, S46.012A, S46.019A, S46.111A, S46.112A, S46.119A, S46.211A, S46.212A, S46.219A, S46.311A, S46.312A, S46.319A, S46.811A, S46.812A, S46.819A, S46.911A, S46.912A, S46.919A, S53.20XA, S53.21XA, S53.22XA, S53.30XA, S53.31XA, S53.32XA, S53.401A, S53.402A, S53.409A, S53.411A, S53.412A, S53.419A, S53.421A, S53.422A, S53.429A, S53.431A, S53.432A, S53.439A, S53.441A, S53.442A, S53.449A, S53.491A, S53.492A, S53.499A, S56.011A, S56.012A, S56.019A, S56.111A, S56.112A, S56.113A, S56.114A, S56.115A, S56.116A, S56.117A, S56.118A, S56.119A, S56.211A, S56.212A, S56.219A, S56.311A, S56.312A, S56.319A, S56.411A, S56.412A, S56.413A, S56.414A, S56.415A, S56.416A, S56.417A, S56.418A, S56.419A, S56.511A, S56.512A, S56.519A, S56.811A, S56.812A, S56.819A, S56.911A, S56.912A, S56.919A, S63.301A, S63.302A, S63.309A, S63.311A, S63.312A, S63.319A, S63.321A, S63.322A, S63.329A, S63.331A, S63.332A, S63.339A, S63.391A, S63.392A, S63.399A, S63.400A, S63.401A, S63.402A, S63.403A, S63.404A, S63.405A, S63.406A, S63.407A, S63.408A, S63.409A, S63.410A, S63.411A, S63.412A, S63.413A, S63.414A, S63.415A, S63.416A, S63.417A, S63.418A, S63.419A, S63.420A, S63.421A, S63.422A, S63.423A, S63.424A, S63.425A, S63.426A, S63.427A, S63.428A, S63.429A, S63.430A, S63.431A, S63.432A, S63.433A, S63.434A, S63.435A, S63.436A, S63.437A, S63.438A, S63.439A, S63.490A, S63.491A, S63.492A, S63.493A, S63.494A, S63.495A, S63.496A, S63.497A, S63.498A, S63.499A, S63.501A, S63.502A, S63.509A, S63.511A, S63.512A, S63.519A, S63.521A, S63.522A, S63.529A, S63.591A, S63.592A, S63.599A, S63.601A, S63.602A, S63.609A, S63.610A, S63.611A, S63.612A, S63.613A, S63.614A, S63.615A, S63.616A, S63.617A, S63.618A, S63.619A, S63.621A, S63.622A, S63.629A, S63.630A, S63.631A, S63.632A, S63.633A, S63.634A, S63.635A, S63.636A, S63.637A, S63.638A, S63.639A, S63.641A, S63.642A, S63.649A, S63.650A, S63.651A, S63.652A, S63.653A, S63.654A, S63.655A, S63.656A, S63.657A, S63.658A, S63.659A, S63.681A, S63.682A, S63.689A, S63.690A, S63.691A, S63.692A, S63.693A, S63.694A, S63.695A, S63.696A, S63.697A, S63.698A, S63.699A, S63.8X1A, S63.8X2A, S63.8X9A, S63.90XA, S63.91XA, S63.92XA, S66.011A, S66.012A, S66.019A, S66.110A, S66.111A, S66.112A, S66.113A, S66.114A, S66.115A, S66.116A, S66.117A, S66.118A, S66.119A, S66.211A, S66.212A, S66.219A, S66.310A, S66.311A, S66.312A, S66.313A, S66.314A, S66.315A, S66.316A, S66.317A, S66.318A, S66.319A, S66.411A, S66.412A, S66.419A, S66.510A, S66.511A, S66.512A, S66.513A, S66.514A, S66.515A, S66.516A, S66.517A, S66.518A, S66.519A, S66.811A, S66.812A, S66.819A, S66.911A, S66.912A, S66.919A, S73.101A, S73.102A, S73.109A, S73.111A, S73.112A, S73.119A, S73.121A, S73.122A, S73.129A, S73.191A, S73.192A,

© 2025 Wolpaw BJ et al. *JAMA Network Open*.

S73.199A, S76.011A, S76.012A, S76.019A, S76.111A, S76.112A, S76.119A, S76.211A, S76.212A, S76.219A, S76.311A, S76.312A, S76.319A, S76.811A, S76.812A, S76.819A, S76.911A, S76.912A, S76.919A, S83.200A, S83.201A, S83.202A, S83.203A, S83.204A, S83.205A, S83.206A, S83.207A, S83.209A, S83.211A, S83.212A, S83.219A, S83.221A, S83.222A, S83.229A, S83.231A, S83.232A, S83.239A, S83.241A, S83.242A, S83.249A, S83.251A, S83.252A, S83.259A, S83.261A, S83.262A, S83.269A, S83.271A, S83.272A, S83.279A, S83.281A, S83.282A, S83.289A, S83.30XA, S83.31XA, S83.32XA, S83.401A, S83.402A, S83.409A, S83.411A, S83.412A, S83.419A, S83.421A, S83.422A, S83.429A, S83.501A, S83.502A, S83.509A, S83.511A, S83.512A, S83.519A, S83.521A, S83.522A, S83.529A, S83.60XA, S83.61XA, S83.62XA, S83.8X1A, S83.8X2A, S83.8X9A, S83.90XA, S83.91XA, S83.92XA, S86.011A, S86.012A, S86.019A, S86.111A, S86.112A, S86.119A, S86.211A, S86.212A, S86.219A, S86.311A, S86.312A, S86.319A, S86.811A, S86.812A, S86.819A, S86.911A, S86.912A, S86.919A, S93.401A, S93.402A, S93.409A, S93.411A, S93.412A, S93.419A, S93.421A, S93.422A, S93.429A, S93.431A, S93.432A, S93.439A, S93.491A, S93.492A, S93.499A, S93.501A, S93.502A, S93.503A, S93.504A, S93.505A, S93.506A, S93.509A, S93.511A, S93.512A, S93.513A, S93.514A, S93.515A, S93.516A, S93.519A, S93.521A, S93.522A, S93.523A, S93.524A, S93.525A, S93.526A, S93.529A, S93.601A, S93.602A, S93.609A, S93.611A, S93.612A, S93.619A, S93.621A, S93.622A, S93.629A, S93.691A, S93.692A, S93.699A, S96.011A, S96.012A, S96.019A, S96.111A, S96.112A, S96.119A, S96.211A, S96.212A, S96.219A, S96.811A, S96.812A, S96.819A, S96.911A, S96.912A, S96.919A, S04.011A, S04.012A, S04.019A, S04.02XA, S04.031A, S04.032A, S04.039A, S04.041A, S04.042A, S04.049A, S04.10XA, S04.11XA, S04.12XA, S04.20XA, S04.21XA, S04.22XA, S04.30XA, S04.31XA, S04.32XA, S04.40XA, S04.41XA, S04.42XA, S04.50XA, S04.51XA, S04.52XA, S04.60XA, S04.61XA, S04.62XA, S04.70XA, S04.71XA, S04.72XA, S04.811A, S04.812A, S04.819A, S04.891A, S04.892A, S04.899A, S04.9XXA, S09.10XA, S09.19XA, S14.2XXA, S14.3XXA, S14.4XXA, S14.5XXA, S14.8XXA, S14.9XXA, S16.8XXA, S16.9XXA, S24.2XXA, S24.3XXA, S24.4XXA, S24.8XXA, S24.9XXA, S29.001A, S29.002A, S29.009A, S29.091A, S29.092A, S29.099A, S34.21XA, S34.22XA, S34.4XXA, S34.5XXA, S34.6XXA, S34.8XXA, S34.9XXA, S39.001A, S39.002A, S39.003A, S39.091A, S39.092A, S39.093A, S44.00XA, S44.01XA, S44.02XA, S44.10XA, S44.11XA, S44.12XA, S44.20XA, S44.21XA, S44.22XA, S44.30XA, S44.31XA, S44.32XA, S44.40XA, S44.41XA, S44.42XA, S44.50XA, S44.51XA, S44.52XA, S44.8X1A, S44.8X2A, S44.8X9A, S44.90XA, S44.91XA, S44.92XA, S46.001A, S46.002A, S46.009A, S46.091A, S46.092A, S46.099A, S46.101A, S46.102A, S46.109A, S46.191A, S46.192A, S46.199A, S46.201A, S46.202A, S46.209A, S46.291A, S46.292A, S46.299A, S46.301A, S46.302A, S46.309A, S46.391A, S46.392A, S46.399A, S46.801A, S46.802A, S46.809A, S46.891A, S46.892A, S46.899A, S46.901A, S46.902A, S46.909A, S46.991A, S46.992A, S46.999A, S54.00XA, S54.01XA, S54.02XA, S54.10XA, S54.11XA, S54.12XA, S54.20XA, S54.21XA, S54.22XA, S54.30XA, S54.31XA, S54.32XA, S54.8X1A, S54.8X2A, S54.8X9A, S54.90XA, S54.91XA, S54.92XA, S56.001A, S56.002A, S56.009A, S56.091A, S56.092A, S56.099A, S56.101A, S56.102A, S56.103A, S56.104A, S56.105A, S56.106A, S56.107A, S56.108A, S56.109A, S56.191A, S56.192A, S56.193A, S56.194A, S56.195A, S56.196A, S56.197A, S56.198A, S56.199A, S56.201A, S56.202A, S56.209A, S56.291A, S56.292A, S56.299A, S56.301A, S56.302A, S56.309A, S56.391A, S56.392A, S56.399A, S56.401A, S56.402A, S56.403A, S56.404A, S56.405A, S56.406A, S56.407A, S56.408A, S56.409A, S56.491A, S56.492A, S56.493A, S56.494A, S56.495A, S56.496A, S56.497A, S56.498A, S56.499A, S56.501A, S56.502A, S56.509A, S56.591A, S56.592A, S56.599A, S56.801A, S56.802A, S56.809A, S56.891A, S56.892A, S56.899A, S56.901A, S56.902A, S56.909A, S56.991A, S56.992A, S56.999A, S64.00XA, S64.01XA, S64.02XA, S64.10XA, S64.11XA, S64.12XA, S64.20XA, S64.21XA, S64.22XA, S64.30XA, S64.31XA, S64.32XA, S64.40XA, S64.490A, S64.491A, S64.492A, S64.493A, S64.494A, S64.495A, S64.496A, S64.497A, S64.498A, S64.8X1A, S64.8X2A, S64.8X9A, S64.90XA, S64.91XA, S64.92XA, S66.001A, S66.002A, S66.009A, S66.091A, S66.092A, S66.099A, S66.100A, S66.101A, S66.102A, S66.103A, S66.104A, S66.105A, S66.106A, S66.107A, S66.108A, S66.109A, S66.190A, S66.191A, S66.192A, S66.193A, S66.194A, S66.195A, S66.196A, S66.197A, S66.198A, S66.199A, S66.201A, S66.202A, S66.209A, S66.291A, S66.292A, S66.299A, S66.300A, S66.301A, S66.302A, S66.303A, S66.304A, S66.305A, S66.306A, S66.307A, S66.308A, S66.309A, S66.390A, S66.391A, S66.392A, S66.393A, S66.394A, S66.395A, S66.396A, S66.397A, S66.398A, S66.399A, S66.401A, S66.402A, S66.409A, S66.491A, S66.492A, S66.499A, S66.500A, S66.501A, S66.502A, S66.503A, S66.504A, S66.505A, S66.506A, S66.507A, S66.508A, S66.509A, S66.590A, S66.591A, S66.592A, S66.593A, S66.594A, S66.595A, S66.596A,

© 2025 Wolpaw BJ et al. *JAMA Network Open*.

	S66.597A, S66.598A, S66.599A, S66.801A, S66.802A, S66.809A, S66.891A, S66.892A, S66.899A, S66.901A, S66.902A, S66.909A, S66.991A, S66.992A, S66.999A, S74.00XA, S74.01XA, S74.02XA, S74.10XA, S74.11XA, S74.12XA, S74.20XA, S74.21XA, S74.22XA, S74.8X1A, S74.8X2A, S74.8X9A, S74.90XA, S74.91XA, S74.92XA, S76.001A, S76.002A, S76.009A, S76.091A, S76.092A, S76.099A, S76.101A, S76.102A, S76.109A, S76.191A, S76.192A, S76.199A, S76.201A, S76.202A, S76.209A, S76.291A, S76.292A, S76.299A, S76.301A, S76.302A, S76.309A, S76.391A, S76.392A, S76.399A, S76.801A, S76.802A, S76.809A, S76.891A, S76.892A, S76.899A, S76.901A, S76.902A, S76.909A, S76.991A, S76.992A, S76.999A, S84.00XA, S84.01XA, S84.02XA, S84.10XA, S84.11XA, S84.12XA, S84.20XA, S84.21XA, S84.22XA, S84.801A, S84.802A, S84.809A, S84.90XA, S84.91XA, S84.92XA, S86.001A, S86.002A, S86.009A, S86.091A, S86.092A, S86.099A, S86.101A, S86.102A, S86.109A, S86.191A, S86.192A, S86.199A, S86.201A, S86.202A, S86.209A, S86.291A, S86.292A, S86.299A, S86.301A, S86.302A, S86.309A, S86.391A, S86.392A, S86.399A, S86.801A, S86.802A, S86.809A, S86.891A, S86.892A, S86.899A, S86.901A, S86.902A, S86.909A, S86.991A, S86.992A, S86.999A, S94.00XA, S94.01XA, S94.02XA, S94.10XA, S94.11XA, S94.12XA, S94.20XA, S94.21XA, S94.22XA, S94.30XA, S94.31XA, S94.32XA, S94.8X1A, S94.8X2A, S94.8X9A, S94.90XA, S94.91XA, S94.92XA, S96.001A, S96.002A, S96.009A, S96.091A, S96.092A, S96.099A, S96.101A, S96.102A, S96.109A, S96.191A, S96.192A, S96.199A, S96.201A, S96.202A, S96.209A, S96.291A, S96.292A, S96.299A, S96.801A, S96.802A, S96.809A, S96.891A, S96.892A, S96.899A, S96.901A, S96.902A, S96.909A, S96.991A, S96.992A, S96.999A, S05.8X1A, S05.8X2A, S05.8X9A, S09.311A, S09.312A, S09.313A, S09.319A, S09.391A, S09.392A, S09.399A, S09.8XXA, S19.80XA, S19.81XA, S19.82XA, S19.83XA, S19.84XA, S19.85XA, S19.89XA, S29.8XXA, S39.81XA, S39.82XA, S39.83XA, S39.840A, S39.848A, S49.80XA, S49.81XA, S49.82XA, S59.801A, S59.802A, S59.809A, S59.811A, S59.812A, S59.819A, S69.80XA, S69.81XA, S69.82XA, S79.811A, S79.812A, S79.819A, S79.821A, S79.822A, S79.829A, S89.80XA, S89.81XA, S89.82XA, S99.811A, S99.812A, S99.819A, S99.821A, S99.822A, S99.829A, T79.0XXA, T79.1XXA, T79.2XXA, T79.4XXA, T79.5XXA, T79.6XXA, T79.7XXA, T79.8XXA, T79.9XXA, T79.A0XA, T79.A11A, T79.A12A, T79.A19A, T79.A21A, T79.A22A, T79.A29A, T79.A3XA, T79.A9XA, O9A.211, O9A.212, O9A.213, O9A.219, O9A.22, O9A.23, S05.90XA, S05.91XA, S05.92XA, S09.301A, S09.302A, S09.309A, S09.90XA, S09.91XA, S09.92XA, S09.93XA, S19.9XXA, S29.9XXA, S39.91XA, S39.92XA, S39.93XA, S39.94XA, S49.90XA, S49.91XA, S49.92XA, S59.901A, S59.902A, S59.909A, S59.911A, S59.912A, S59.919A, S69.90XA, S69.91XA, S69.92XA, S79.911A, S79.912A, S79.919A, S79.921A, S79.922A, S79.929A, S89.90XA, S89.91XA, S89.92XA, S99.911A, S99.912A, S99.919A, S99.921A, S99.922A, S99.929A, T07, T07.XXXA, T14.8, T14.8XXA, T14.90, T14.90XA, T14.91, T14.91XA
**Combined Comorbidity Score (Pre-existing/Chronic) Diagnoses**	
Metastatic cancer (weight 5)	C45.9, C77.0, C77.1, C77.2, C77.3, C77.4, C77.5, C77.6, C77.7, C77.8, C77.9, C78.0, C78.00, C78.01, C78.02, C78.1, C78.2, C78.3, C78.30, C78.39, C78.4, C78.5, C78.6, C78.7, C78.8, C78.80, C78.89, C79.0, C79.00, C79.01, C79.02, C79.1, C79.10, C79.11, C79.19, C79.2, C79.3, C79.31, C79.32, C79.4, C79.40, C79.49, C79.5, C79.51, C79.52, C79.6, C79.60, C79.61, C79.62, C79.63, C79.7, C79.70, C79.71, C79.72, C79.8, C79.81, C79.82, C79.89, C79.9, C80, C80.0, C80.1, C80.2
Congestive heart failure (weight 2)	A18.84, I09.9, I11.0, I13.0, I13.2, I25.5, I42, I42.0, I42.1, I42.2, I42.3, I42.4, I42.5, I42.6, I42.7, I42.8, I42.9, I43, I50, I50.1, I50.2, I50.20, I50.21, I50.22, I50.23, I50.3, I50.30, I50.31, I50.32, I50.33, I50.4, I50.40, I50.41, I50.42, I50.43, I50.8, I50.81, I50.810, I50.811, I50.812, I50.813, I50.814, I50.82, I50.83, I50.84, I50.89, I50.9, I51.7, P29.0
Dementia (weight 2)	F01, F01.5, F01.50, F01.51, F01.511, F01.518, F01.52, F01.53, F01.54, F01.A, F01.A0, F01.A1, F01.A11, F01.A18, F01.A2, F01.A3, F01.A4, F01.B, F01.B1, F01.B11, F01.B18, F01.B2, F01.B3, F01.B4, F01.C, F01.C0, F01.C1, F01.C11, F01.C18, F01.C2, F01.C3, F01.C4, F02, F02.8, F02.80, F02.81, F02.811, F02.818, F02.82, F02.83, F02.84, F02.A, F02.A0, F02.A1, F02.A11, F02.A18, F02.A2, F02.A3, F02.A4, F02.B, F02.B1, F02.B11, F02.B18, F02.B2, F02.B3, F02.B4, F02.C, F02.C0, F02.C1, F02.C11, F02.C18, F02.C2, F02.C3, F02.C4, F03, F03.9, F03.90, F03.91, F03.911, F03.918, F03.92, F03.93, F03.94, F03.A, F03.A0, F03.A1, F03.A11, F03.A18, F03.A2, F03.A3, F03.A4, F03.B, F03.B0, F03.B1, F03.B11, F03.B18, F03.B2, F03.B3, F03.B4, F03.C, F03.C0, F03.C1, F03.C11, F03.C18, F03.C2, F03.C3, F03.C4, F05, G30, G30.0, G30.1, G30.8, G30.9, G31.01, G31.09, G31.1
Renal failure (weight 2)	I12.0, I13, I13.0, I13.1, I13.10, I13.11, I13.2, N03.2, N03.3, N03.4, N03.5, N03.6, N03.7, N05.2, N05.3, N05.4, N05.5, N05.6, N05.7, N18, N18.1, N18.2, N18.3, N18.30, N18.31, N18.32, N18.4,

© 2025 Wolpaw BJ et al. *JAMA Network Open*.

	N18.5, N18.6, N18.9, N19, N25.0, Z39.32, Z48.22, Z49, Z49.0, Z49.01, Z49.02, Z49.3, Z49.31, Z49.32, Z91.15, Z94.0, Z99.2
Weight loss (weight 2)	E40, E41, E42, E43, E44, E44.0, E44.1, E45, E46, E64, E64.0, E64.1, E64.2, E64.3, E64.8, E64.9, R63.4, R64
Hemiplegia (weight 1)	G04.1, G11.4, G80.1, G80.2, G81, G81.0, G81.00, G81.01, G81.02, G81.03, G81.04, G81.1, G81.10, G81.11, G81.12, G81.13, G81.14, G81.9, G81.90, G81.91, G81.92, G81.93, G81.94, G82, G82.2, G82.20, G82.21, G82.22, G82.5, G82.50, G82.51, G82.52, G82.53, G82.54, G83, G83.0, G83.1, G83.10, G83.11, G83.12, G83.13, G83.14, G83.2, G83.20, G83.21, G83.22, G83.23, G83.24, G83.3, G83.30, G83.31, G83.32, G83.33, G83.34, G83.4, G83.5, G83.8, G83.81, G83.82, G83.83, G83.84, G83.9, G83.9
Alcohol abuse (weight 1)	F10.10, F10.120, F10.121, F10.129, F10.130, F10.131, F10.132, F10.139, F10.14, F10.150, F10.151, F10.159, F10.180, F10.181, F10.182, F10.188, F10.19, F10.20, F10.220, F10.221, F10.229, F10.230, F10.231, F10.232, F10.239, F10.24, F10.250, F10.251, F10.259, F10.26, F10.27, F10.280, F10.281, F10.282, F10.288, F10.29, F10.920, F10.921, F10.929, F10.930, F10.931, F10.932, F10.939, F10.94, F10.950, F10.951, F10.959, F10.96, F10.97, F10.980, F10.981, F10.982, F10.988, F10.99, E52, G62.1, I42.6, K29.2, K70.0, K70.3, K70.9, T51, T51.0, T51.0X, T51.0X1, T51.0X1A, T51.0X1D, T51.0X1S, T51.0X2, T51.0X2A, T51.0X2D, T51.0X2S, T51.0X3, T51.0X3A, T51.0X3D, T51.0X3S, T51.0X4, T51.0X4A, T51.0X4D, T51.0X4S, T51.1, T51.1X, T51.1X1, T51.1X1A, T51.1X1D, T51.1X1S, T51.1X2, T51.1X2A, T51.1X2D, T51.1X2S, T51.1X3, T51.1X3A, T51.1X3D, T51.1X3S, T51.2, T51.2X, T51.2X1, T51.2X1A, T51.2X1D, T51.2X1S, T51.2X2, T51.2X2A, T51.2X2D, T51.2X2S, T51.2X3, T51.2X3A, T51.2X3D, T51.2X3S, T51.2X4, T51.2X4A, T51.2X4D, T51.2X4S, T51.3, T51.3X, T51.3X1, T51.3X1A, T51.3X1D, T51.3X1S, T51.3X2, T51.3X2A, T51.3X2D, T51.3X2S, T51.3X3, T51.3X3A, T51.3X3D, T51.3X3S, T51.3X4, T51.3X4A, T51.3X4D, T51.3X4S, T51.8, T51.8X, T51.8X1, T51.8X1A, T51.8X1D, T51.8X1S, T51.8X2, T51.8X2A, T51.8X2D, T51.8X2S, T51.8X3, T51.8X3A, T51.8X3D, T51.8X3S, T51.8X4, T51.8X4A, T51.8X4D, T51.8X4S, T51.9, T51.91, T51.91XA, T51.91XD, T51.91XS, T51.92, T51.92XA, T51.92XD, T51.92XS, T51.93, T51.93XA, T51.93XD, T51.93XS, T51.94, T51.94XA, T51.94XD, T51.94XS, Z71.4, Z71.41, Z71.42, Z71.5, Z71.51, Z71.52, Z71.6, Z71.7, Z71.8, Z71.81, Z71.82, Z71.83, Z71.84, Z71.85, Z71.87, Z71.88, Z71.89, Z71.9, Z65.8
Any tumor (weight 1)	C00.0, C00.1, C00.2, C00.3, C00.4, C00.5, C00.6, C00.8, C00.9, C01, C02.0, C02.1, C02.2, C02.3, C02.4, C02.8, C02.9, C03.0, C03.1, C03.9, C04.0, C04.1, C04.8, C04.9, C05.0, C05.1, C05.2, C05.8, C05.9, C06.0, C06.1, C06.2, C06.80, C06.89, C06.9, C07, C08.0, C08.1, C08.9, C09.0, C09.1, C09.8, C09.9, C10.0, C10.1, C10.2, C10.3, C10.4, C10.8, C10.9, C11.0, C11.1, C11.2, C11.3, C11.8, C11.9, C12, C13.0, C13.1, C13.2, C13.8, C13.9, C14.0, C14.2, C14.8, C15.3, C15.4, C15.5, C15.8, C15.9, C16.0, C16.1, C16.2, C16.3, C16.4, C16.5, C16.6, C16.8, C16.9, C17.0, C17.1, C17.2, C17.3, C17.8, C17.9, C18.0, C18.1, C18.2, C18.3, C18.4, C18.5, C18.6, C18.7, C18.8, C18.9, C19, C20, C21.0, C21.1, C21.2, C21.8, C22.0, C22.1, C22.2, C22.3, C22.4, C22.7, C22.8, C22.9, C23, C24.0, C24.1, C24.8, C24.9, C25.0, C25.1, C25.2, C25.3, C25.4, C25.7, C25.8, C25.9, C26.0, C26.1, C26.9, C30.0, C30.1, C31.0, C31.1, C31.2, C31.3, C31.8, C31.9, C32.0, C32.1, C32.2, C32.3, C32.8, C32.9, C33, C34.00, C34.01, C34.02, C34.10, C34.11, C34.12, C34.2, C34.30, C34.31, C34.32, C34.80, C34.81, C34.82, C34.90, C34.91, C34.92, C37, C38.0, C38.1, C38.2, C38.3, C38.4, C38.8, C39.0, C39.9, C40.00, C40.01, C40.02, C40.10, C40.11, C40.12, C40.20, C40.21, C40.22, C40.30, C40.31, C40.32, C40.80, C40.81, C40.82, C40.90, C40.91, C40.92, C41.0, C41.1, C41.2, C41.3, C41.4, C41.9, C43.0, C43.10, C43.11, C43.111, C43.112, C43.12, C43.121, C43.122, C43.20, C43.21, C43.22, C43.30, C43.31, C43.39, C43.4, C43.51, C43.52, C43.59, C43.60, C43.61, C43.62, C43.70, C43.71, C43.72, C43.8, C43.9, C45.0, C45.1, C45.2, C45.7, C45.9, C46.0, C46.1, C46.2, C46.3, C46.4, C46.50, C46.51, C46.52, C46.7, C46.9, C47.0, C47.10, C47.11, C47.12, C47.20, C47.21, C47.22, C47.3, C47.4, C47.5, C47.6, C47.8, C47.9, C48.0, C48.1, C48.2, C48.8, C48.8, C49.0, C49.10, C49.11, C49.12, C49.20, C49.21, C49.22, C49.3, C49.4, C49.5, C49.6, C49.8, C49.9, C49.A0, C49.A1, C49.A2, C49.A3, C49.A4, C49.A5, C49.A9, C50.011, C50.012, C50.019, C50.021, C50.022, C50.029, C50.111, C50.112, C50.119, C50.121, C50.122, C50.129, C50.211, C50.212, C50.219, C50.221, C50.222, C50.229, C50.311, C50.312, C50.319, C50.321, C50.322, C50.329, C50.411, C50.412, C50.419, C50.421, C50.422, C50.429, C50.511, C50.512, C50.519, C50.521, C50.522, C50.529, C50.611, C50.612, C50.619, C50.621, C50.622, C50.629, C50.811, C50.812, C50.819, C50.821, C50.822, C50.829, C50.911, C50.912, C50.919, C50.921, C50.922, C50.929, C51.0, C51.1, C51.2, C51.8, C51.9, C52, C53.0, C53.1, C53.8, C53.9, C54.0, C54.1, C54.2, C54.3, C54.8, C54.9, C55, C56.1, C56.2, C56.3, C56.9, C57.00, C57.01, C57.02, C57.10, C57.11, C57.12, C57.20, C57.21, C57.22, C57.3, C57.4, C57.7, C57.8, C57.9, C58, C60.0, C60.1, C60.2, C60.8, C60.9, C61, C62.00, C62.01, C62.02, C62.10, C62.11, C62.12, C62.90, C62.91, C62.92, C63.00, C63.01, C63.02, C63.10,

© 2025 Wolpaw BJ et al. *JAMA Network Open*.

	C63.11, C63.12, C63.2, C63.7, C63.8, C63.9, C64.1, C64.2, C64.9, C65.1, C65.2, C65.9, C66.1, C66.2, C66.9, C67.0, C67.1, C67.2, C67.3, C67.4, C67.5, C67.6, C67.7, C67.8, C67.9, C68.0, C68.1, C68.8, C68.9, C69.00, C69.01, C69.02, C69.10, C69.11, C69.12, C69.20, C69.21, C69.22, C69.30, C69.31, C69.32, C69.40, C69.41, C69.42, C69.50, C69.51, C69.52, C69.60, C69.61, C69.62, C69.80, C69.81, C69.82, C69.90, C69.91, C69.92, C70.0, C70.1, C70.9, C71.0, C71.1, C71.2, C71.3, C71.4, C71.5, C71.6, C71.7, C71.8, C71.9, C72.0, C72.1, C72.20, C72.21, C72.22, C72.30, C72.31, C72.32, C72.40, C72.41, C72.42, C72.50, C72.59, C72.9, C73, C74.00, C74.01, C74.02, C74.10, C74.11, C74.12, C74.90, C74.91, C74.92, C75.0, C75.1, C75.2, C75.3, C75.4, C75.5, C75.8, C75.9, C76.0, C76.1, C76.2, C76.3, C76.40, C76.41, C76.42, C76.50, C76.51, C76.52, C76.8, C81.00, C81.01, C81.02, C81.03, C81.04, C81.05, C81.06, C81.07, C81.08, C81.09, C81.10, C81.11, C81.12, C81.13, C81.14, C81.15, C81.16, C81.17, C81.18, C81.19, C81.20, C81.21, C81.22, C81.23, C81.24, C81.25, C81.26, C81.27, C81.28, C81.29, C81.30, C81.31, C81.32, C81.33, C81.34, C81.35, C81.36, C81.37, C81.38, C81.39, C81.40, C81.41, C81.42, C81.43, C81.44, C81.45, C81.46, C81.47, C81.48, C81.49, C81.70, C81.71, C81.72, C81.73, C81.74, C81.75, C81.76, C81.77, C81.78, C81.79, C81.90, C81.91, C81.92, C81.93, C81.94, C81.95, C81.96, C81.97, C81.98, C81.99, C82.00, C82.01, C82.02, C82.03, C82.04, C82.05, C82.06, C82.07, C82.08, C82.09, C82.10, C82.11, C82.12, C82.13, C82.14, C82.15, C82.16, C82.17, C82.18, C82.19, C82.20, C82.21, C82.22, C82.23, C82.24, C82.25, C82.26, C82.27, C82.28, C82.29, C82.30, C82.31, C82.32, C82.33, C82.34, C82.35, C82.36, C82.37, C82.38, C82.39, C82.40, C82.41, C82.42, C82.43, C82.44, C82.45, C82.46, C82.47, C82.48, C82.49, C82.50, C82.51, C82.52, C82.53, C82.54, C82.55, C82.56, C82.57, C82.58, C82.59, C82.60, C82.61, C82.62, C82.63, C82.64, C82.65, C82.66, C82.67, C82.68, C82.69, C82.80, C82.81, C82.82, C82.83, C82.84, C82.85, C82.86, C82.87, C82.88, C82.89, C82.90, C82.91, C82.92, C82.93, C82.94, C82.95, C82.96, C82.97, C82.98, C82.99, C83.00, C83.01, C83.02, C83.03, C83.04, C83.05, C83.06, C83.07, C83.08, C83.09, C83.10, C83.11, C83.12, C83.13, C83.14, C83.15, C83.16, C83.17, C83.18, C83.19, C83.30, C83.31, C83.32, C83.33, C83.34, C83.35, C83.36, C83.37, C83.38, C83.39, C83.50, C83.51, C83.52, C83.53, C83.54, C83.55, C83.56, C83.57, C83.58, C83.59, C83.70, C83.71, C83.72, C83.73, C83.74, C83.75, C83.76, C83.77, C83.78, C83.79, C83.80, C83.81, C83.82, C83.83, C83.84, C83.85, C83.86, C83.87, C83.88, C83.89, C83.90, C83.91, C83.92, C83.93, C83.94, C83.95, C83.96, C83.97, C83.98, C83.99, C84.00, C84.01, C84.02, C84.03, C84.04, C84.05, C84.06, C84.07, C84.08, C84.09, C84.10, C84.11, C84.12, C84.13, C84.14, C84.15, C84.16, C84.17, C84.18, C84.19, C84.40, C84.41, C84.42, C84.43, C84.44, C84.45, C84.46, C84.47, C84.48, C84.49, C84.60, C84.61, C84.62, C84.63, C84.64, C84.65, C84.66, C84.67, C84.68, C84.69, C84.70, C84.71, C84.72, C84.73, C84.74, C84.75, C84.76, C84.77, C84.78, C84.79, C84.7A, C84.90, C84.91, C84.92, C84.93, C84.94, C84.95, C84.96, C84.97, C84.98, C84.99, C84.A0, C84.A1, C84.A2, C84.A3, C84.A4, C84.A5, C84.A6, C84.A7, C84.A8, C84.A9, C84.Z0, C84.Z1, C84.Z2, C84.Z3, C84.Z4, C84.Z5, C84.Z6, C84.Z7, C84.Z8, C84.Z9, C85.10, C85.11, C85.12, C85.13, C85.14, C85.15, C85.16, C85.17, C85.18, C85.19, C85.20, C85.21, C85.22, C85.23, C85.24, C85.25, C85.26, C85.27, C85.28, C85.29, C85.80, C85.81, C85.82, C85.83, C85.84, C85.85, C85.86, C85.87, C85.88, C85.89, C85.90, C85.91, C85.92, C85.93, C85.94, C85.95, C85.96, C85.97, C85.98, C85.99, C86.0, C86.1, C86.2, C86.3, C86.4, C86.5, C86.6, C88.0, C88.2, C88.3, C88.4, C88.8, C88.9, C90.00, C90.01, C90.02, C90.10, C90.11, C90.12, C90.20, C90.21, C90.22, C90.30, C90.31, C90.32, C91.00, C91.01, C91.02, C91.10, C91.11, C91.12, C91.30, C91.31, C91.32, C91.40, C91.41, C91.42, C91.50, C91.51, C91.52, C91.60, C91.61, C91.62, C91.90, C91.91, C91.92, C91.A0, C91.A1, C91.A2, C91.Z0, C91.Z1, C91.Z2, C92.00, C92.01, C92.02, C92.10, C92.11, C92.12, C92.20, C92.21, C92.22, C92.30, C92.31, C92.32, C92.40, C92.41, C92.42, C92.50, C92.51, C92.52, C92.60, C92.61, C92.62, C92.90, C92.91, C92.92, C92.A0, C92.A1, C92.A2, C92.Z0, C92.Z1, C92.Z2, C93.00, C93.01, C93.02, C93.10, C93.11, C93.12, C93.30, C93.31, C93.32, C93.90, C93.91, C93.92, C93.Z0, C93.Z1, C93.Z2, C94.00, C94.01, C94.02, C94.20, C94.21, C94.22, C94.30, C94.31, C94.32, C94.80, C94.81, C94.82, C95.00, C95.01, C95.02, C95.10, C95.11, C95.12, C95.90, C95.91, C95.92, C96.0, C96.2, C96.20, C96.21, C96.22, C96.29, C96.4, C96.5, C96.6, C96.9, C96.A, C96.Z, D45, D89, D89.0, D89.1, D89.2, D89.3, D89.4, D89.40, D89.41, D89.42, D89.43, D89.44, D89.49, D89.8, D89.81, D89.810, D89.811, D89.812, D89.813, D89.82, D89.83, D89.831, D89.832, D89.833, D89.834, D89.835, D89.839, D89.89, D89.9, Z85.46
Cardiac arrhythmias (weight 1)	I44.0, I44.1, I44.3, I44.30, I44.39, I44.4, I44.5, I44.6, I44.60, I44.69, I44.7, I45.0, I45.1, I45.2, I45.4, I45.5, I45.6, I45.7, I45.8, I45.9, I47, I47.0, I47.1, I47.2, I47.20, I47.21, I47.29, I47.9, I48, I48.0, I48.1, I48.11, I48.19, I48.2, I48.20, I48.21, I48.3, I48.4, I48.9, I48.9, I48.91, I48.92, I49, I49.0, I49.01, I49.02, I49.2, I49.3, I49.4, I49.40, I49.49, I49.5, I49.5, I49.8, I49.9, R00.0, R00.1, R00.8,

© 2025 Wolpaw BJ et al. *JAMA Network Open*.

	T82.1, T82.11, T82.110, T82/110A, T82.110D, T82.110S, T82.111, T82.111A, T82.111D, T82.111S, T82.118, T82.118A, T82.118D, T82.118S, T82.119, T82.119A, T82.119D, T82.119S, T82.12, T82.120, T82.120A, T82.120D, T82.120S, T82.121, T82.121A, T82.121D, T82.121S, T82.128, T82.128A, T82.128D, T82.128S, T82.129, T82.129A, T82.129D, T82.129S, T82.19, T82.190, T82.190A, T82.190D, T82.190S, T82.191, T82.191A, T82.191D, T82.191S, T82.198, T82.198A, T82.198D, T82.198S, T82.199, T82.199A, T82.199D, T82.199S, Z45.0, Z95.0, Z95.810, Z95.818, Z95.9
Chronic pulmonary disease (weight 1)	I26.0, I26.01, I26.02, I26.09, I27.2, I27.9, J40, J41, J41.0, J41.1, J41.8, J42, J43, J43.0, J43.1, J43.2, J43.8, J43.9, J44, J44.0, J44.1, J44.9, J45.20, J45.21, J45.22, J45.30, J45.31, J45.32, J45.40, J45.41, J45.42, J45.50, J45.51, J45.52, J45.901, J45.902, J45.909, J45.990, J45.991, J45.998, J47, J47.0, J47.1, J47.9, J60, J61, J62, J62.0, J62.8, J63, J63.0, J63.1, J63.2, J63.3, J63.4, J63.5, J63.6, J64, J65, J66, J66.0, J66.1, J66.2, J66.8, J67, J67.0, J67.1, J67.2, J67.3, J67.4, J67.5, J67.6, J67.7, J67.8, J67.9, J68.4, J70.1, J70.3
Coagulopathy (weight 1)	D65, D66, D67, D68, D68.0, D68.00, D68.01, D68.02, D68.020, D68.021, D68.022, D68.023, D68.029, D68.03, D68.04, D68.09, D68.1, D68.2, D68.3, D68.31, D68.311, D68.312, D68.318, D68.32, D68.4, D68.5, D68.51, D68.52, D68.59, D68.6, D68.61, D68.62, D68.69, D68.8, D68.9, D69.1, D69.3, D69.4, D69.41, D69.42, D69.49, D69.5, D69.51, D69.59, D69.6
Complicated diabetes (weight 1)	E10.21, E10..22, E10.29, E10.311, E10.319, E10.321, E10.3211, E10.3212, E10.3213, E10.3219, E10.329, E10.3291, E10.3292, E10.3293, E10.3299, E10.331, E10.3311, E10.3312, E10.3313, E10.3319, E10.339, E10.3391, E10.3392, E10.3393, E10.3399, E10.341, E10.3411, E10.3412, E10.3413, E10.3419, E10.349, E10.3491, E10.3492, E10.3493, E10.3499, E10.351, E10.3511, E10.3512, E10.3513, E10.3519, E10.3521, E10.3522, E10.3523, E10.3529, E10.3531, E10.3532, E10.3533, E10.3539, E10.3541, E10.3542, E10.3543, E10.3549, E10.3551, E10.3552, E10.3553, E10.3559, E10.359, E10.3591, E10.3592, E10.3593, E10.3599, E10.36, E10.37X1, E10.37X2, E10.37X3, E10.37X9, E10.39, E10.40, E10.41, E10.42, E10.43, E10.44, E10.49, E10.51, E10.52, E10.59, E10.610, E10.618, E10.620, E10.621, E10.622, E10.628, E10.630, E10.638, E10.641, E10.649, E10.65, E10.69, E10.8, E11.21, E11.22, E11.29, E11.311, E11.319, E11.321, E11.3211, E11.3212, E11.3213, E11.3219, E11.329, E11.3291, E11.3292, E11.3293, E11.3299, E11.331, E11.3311, E11.3312, E11.3313, E11.3319, E11.339, E11.3391, E11.3392, E11.3393, E11.3399, E11.341, E11.3411, E11.3412, E11.3413, E11.3419, E11.349, E11.3491, E11.3492, E11.3493, E11.3499, E11.351, E11.3511, E11.3512, E11.3513, E11.3519, E11.3521, E11.3522, E11.3523, E11.3529, E11.3531, E11.3532, E11.3533, E11.3539, E11.3541, E11.3542, E11.3543, E11.3549, E11.3551, E11.3552, E11.3553, E11.3559, E11.359, E11.3591, E11.3592, E11.3593, E11.3599, E11.36, E11.37X1, E11.37X2, E11.37X3, E11.37X9, E11.39, E11.40, E11.41, E11.42, E11.43, E11.44, E11.49, E11.51, E11.52, E11.59, E11.610, E11.618, E11.620, E11.621, E11.622, E11.628, E11.630, E11.638, E11.641, E11.649, E11.65, E11.69, E11.8, E13.21, E13.22, E13.29, E13.311, E13.319, E13.321, E13.3211, E13.3212, E13.3213, E13.3219, E13.329, E13.3291, E13.3292, E13.3293, E13.3299, E13.331, E13.3311, E13.3312, E13.3313, E13.3319, E13.339, E13.3391, E13.3392, E13.3393, E13.3399, E13.341, E13.3411, E13.3412, E13.3413, E13.3419, E13.349, E13.3491, E13.3492, E13.3493, E13.3499, E13.351, E13.3511, E13.3512, E13.3513, E13.3519, E13.3521, E13.3522, E13.3523, E13.3529, E13.3531, E13.3532, E13.3533, E13.3539, E13.3541, E13.3542, E13.3543, E13.3549, E13.3551, E13.3552, E13.3553, E13.3559, E13.359, E13.3591, E13.3592, E13.3593, E13.3599, E13.36, E13.37X1, E13.37X2, E13.37X3, E13.37X9, E13.39, E13.40, E13.41, E13.42, E13.43, E13.44, E13.49, E13.51, E13.52, E13.59, E13.610, E13.618, E13.620, E13.621, E13.622, E13.628, E13.630, E13.638, E13.641, E13.649, E13.65, E13.69, E13.8
Deficiency anemias (weight 1)	D50, D50.0, D50.1, D50.8, D50.9, D51, D51.0, D51.1, D51.2, D51.3, D51.8, D51.9, D52, D52.0, D52.1, D52.8, D52.9, D53, D53.0, D53.0, D53.1, D53.2, D53.8, D53.9, D64.9
Fluid and electrolyte disorders (weight 1)	E22.2, E86, E86.0, E86.1, E86.9, E87, E87.0, E87.1, E87.2, E87.20, E87.21, E87.22, E87.29, E87.3, E87.4, E87.5, E87.6, E87.7, E87.70, E87.71, E87.79, E87.8
Liver disease (weight 1)	B18, B18.0, B18.1, B18.2, B18.8, B18.9, I85, I85.0, I85.00, I85.01, I85.1, I85.10, I85.11, I86.4, K70.0, K70.2, K70.30, K70.31, K70.40, K70.41, K70.9, K71.0, K71.10, K71.11, K71.7, K71.8, K71.9, K72.00, K72.01, K72.10, K72.11, K72.90, K72.91, K74.0, K74.00, K74.01, K74.02, K74.1, K74.2, K74.3, K74.4, K74.5, K74.60, K74.69, K75.4, K75.81, K76.0, K76.2, K76.3, K76.4, K76.5, K76.6, K76.7, K76.8, K76.81, K76.82, K76.89, K76.9, Z48.23, Z94.4

© 2025 Wolpaw BJ et al. *JAMA Network Open*.

Peripheral vascular disorder (weight 1)	E08.51, E08.52, E09.51, E09.52, E10.51, E10.52, E11.51, E13.51, E13.52, I67.0, I70.0, I70.1, I70.201, I70.202, I70.203, I70.208, I70.209, I70.211, I70.212, I70.213, I70.218, I70.219, I70.221, I70.222, I70.223, I70.228, I70.229, I70.231, I70.232, I70.233, I70.234, I70.235, I70.238, I70.239, I70.241, I70.242, I70.243, I70.244, I70.245, I70.248, I70.249, I70.25, I70.261, I70.262, I70.263, I70.268, I70.269, I70.291, I70.292, I70.293, I70.298, I70.299, I70.301, I70.302, I70.303, I70.308, I70.309, I70.311, I70.312, I70.313, I70.318, I70.319, I70.321, I70.322, I70.323, I70.328, I70.329, I70.331, I70.332, I70.333, I70.334, I70.335, I70.338, I70.339, I70.341, I70.342, I70.343, I70.344, I70.345, I70.348, I70.349, I70.35, I70.361, I70.362, I70.363, I70.368, I70.369, I70.391, I70.392, I70.393, I70.398, I70.399, I70.401, I70.402, I70.403, I70.408, I70.409, I70.411, I70.412, I70.413, I70.418, I70.419, I70.421, I70.422, I70.423, I70.428, I70.429, I70.431, I70.432, I70.433, I70.434, I70.435, I70.438, I70.439, I70.441, I70.442, I70.443, I70.444, I70.445, I70.448, I70.449, I70.45, I70.461, I70.462, I70.463, I70.468, I70.469, I70.491, I70.492, I70.493, I70.498, I70.499, I70.501, I70.502, I70.503, I70.508, I70.509, I70.511, I70.512, I70.513, I70.518, I70.519, I70.521, I70.522, I70.523, I70.528, I70.529, I70.531, I70.532, I70.533, I70.534, I70.535, I70.538, I70.539, I70.541, I70.542, I70.543, I70.544, I70.545, I70.548, I70.549, I70.55, I70.561, I70.562, I70.563, I70.568, I70.569, I70.591, I70.592, I70.593, I70.598, I70.599, I70.601, I70.602, I70.603, I70.608, I70.609, I70.611, I70.612, I70.613, I70.618, I70.619, I70.621, I70.622, I70.623, I70.628, I70.629, I70.631, I70.632, I70.633, I70.634, I70.635, I70.638, I70.639, I70.641, I70.642, I70.643, I70.644, I70.645, I70.648, I70.649, I70.65, I70.661, I70.662, I70.663, I70.668, I70.669, I70.691, I70.692, I70.693, I70.698, I70.699, I70.701, I70.702, I70.703, I70.708, I70.709, I70.711, I70.712, I70.713, I70.718, I70.719, I70.721, I70.722, I70.723, I70.728, I70.729, I70.731, I70.732, I70.733, I70.734, I70.735, I70.738, I70.739, I70.741, I70.742, I70.743, I70.744, I70.745, I70.748, I70.749, I70.75, I70.761, I70.762, I70.763, I70.768, I70.769, I70.791, I70.792, I70.793, I70.798, I70.799, I70.8, I70.90, I70.91, I70.92, I71.00, I71.01, I71.02, I71.03, I71.1, I71.2, I71.3, I71.4, I71.5, I71.6, I71.8, I71.9, I73.1, I73.8, I73.81, I73.89, I73.9, I71.1, I71.10, I71.11, I71.12, I71.13, I77.71, I77.74, I77.79, I79, I79.0, I79.1, I79.8, K55.9, Z95.82, Z95.9
Psychosis (weight 1)	F20, F20.0, F20.1, F20.2, F20.3, F20.5, F20.81, F20.89, F20.9, F22, F23, F24, F25, F25.0, F25.1, F25.8, F25.9, F28, F29, F30.11, F33.3, F34.8, F34.9, F39, F44.89, F84.3
Pulmonary circulation disorders (weight 1)	I26.0, I26.01, I26.02, I26.09, I27.2, I27.20, I27.21, I27.22, I27.23, I27.24, I27.29, I27.8, I27.81, I27.82, I27.83, I27.89, I27.9, I28, I28.0, I28.1, I28.8, I28.9
HIV/AIDS (weight -1)	B20
Hypertension (weight -1)	I10, I11, I11.0, I11.9, I13, I13.0, I13.1, I13.10, I13.11, I13.2, I15, I15.0, I15.1, I15.2, I15.8, I15.9, N26.2
Other Pre-existing/Chronic Conditions Relevant to Treatment of AWS	
Prior AWS	F10.230 , F10.231, F10.232, F10.239, F10.130, F10.131, F10.132, F10.139, F10.930, F10.931, F10.932, F10.939
Prior AUD (including in remission) or alcohol-related diagnoses	F10.180, F10.14, F10.150, F10.151, F10.159, F10.181, F10.182, F10.121, F10.120, F10.129, F10.188, F10.19, F10.131, F10.132, F10.130, F10.139, F10.10, F10.280, F10.24, F10.26, F10.27, F10.250, F10.251, F10.259, F10.281, F10.282, F10.221, F10.220, F10.229, F10.288, F10.29, F10.231, F10.232, F10.230, F10.239, F10.20, F10.11, F10.21, F10.10, F10.120, F10.121, F10.129, F10.130, F10.131, F10.132, F10.139, F10.14, F10.150, F10.151, F10.159, F10.180, F10.181, F10.182, F10.188, F10.19, F10.20, F10.220, F10.221, F10.229, F10.230, F10.231, F10.232, F10.239, F10.24, F10.250, F10.251, F10.259, F10.26, F10.27, F10.280, F10.281, F10.282, F10.288, F10.29, F10.920, F10.921, F10.929, F10.930, F10.931, F10.932, F10.939, F10.94, F10.950, F10.951, F10.959, F10.96, F10.97, F10.980, F10.981, F10.982, F10.988, F10.99, G31.2, G62.1, I42.6, K29.20, K29.21, K70.0, K70.10, K70.11, K70.2, K70.30, K70.31, K70.40, K70.41, K70.9, O35.4XX0, O35.4XX1, O35.4XX2, O35.4XX3, O35.4XX4, O35.4XX5, O35.4XX9, O99.310, O99.311, O99.312, O99.313, O99.314, O99.315, Y90.0, Y90.1, Y90.2, Y90.3, Y90.4, Y90.5, Y90.6, Y90.7, Y90.8, Y90.9

Abbreviations: AIDS=acquired immunodeficiency syndrome, AUD=alcohol use disorder, AWS=alcohol withdrawal syndrome, HIV=human immunodeficiency virus, ICD-10=International Classification of Diseases, 10th Edition

© 2025 Wolpaw BJ et al. *JAMA Network Open*.

eTable 4. Standardized mean differences in characteristics of pre- and post- phenobarbital orderset groups—used to identify covariates for inclusion in multivariable regression models (i.e., analyses adjusted for baseline group differences)

	**Total** (n = 254)	**Pre-Phenobarbital Orderset** (n = 154)	**Post-Phenobarbital Orderset** (n = 100)	**SMD** [a]
**Sociodemographic**				
Age, mean (sd)	53.0 (14.9)	53.1 (14.7)	52.7 (15.3)	-0.03
Sex, no. (%)				
Female	84 (33.1)	53 (34.4)	31 (31.0)	-0.07
Male	170 (66.9)	101 (65.6)	69 (69.0)	0.07
Race categories, no. (%)				-0.09 [b]
American Indian or Alaska Native	11 (4.3)	4 (2.6)	7 (7.0)	
Asian or Native Hawaiian or Pacific Islander	6 (2.4)	4 (2.6)	2 (2.0)	
Black	14 (5.5)	10 (6.5)	4 (4.0)	
White	212 (83.5)	134 (84.2)	82 (82.0)	
Multiple races	8 (3.2)	5 (3.3)	3 (3.0)	
Other or Unknown	3 (1.2)	1 (0.7)	2 (2.0)	
Hispanic or Latinx ethnicity, no. (%)	22 (8.7)	12 (7.8)	10 (10.0)	0.08
Documented homelessness, no. (%)	10 (3.9)	4 (2.6)	6 (6.0)	0.17
Insurance, no. (%)				0.01
Commercial	53 (20.9)	30 (19.5)	23 (23.0)	
Medicaid	118 (46.5)	74 (48.1)	44 (44.0)	
Medicare	68 (26.8)	39 (25.3)	29 (29.0)	
Self-pay	14 (5.5)	10 (6.5)	4 (4.0)	
Tricare	1 (0.4)	1 (0.7)	0 (0)	
**Clinical**				
Prior encounter(s) within the health care system	152 (59.8)	96 (62.3)	56 (56.0)	-0.13
Severe pre-existing morbidity (CCS ≥ 5),[b] no./total no. (%)	14/152 (9.2)	8/96 (8.3)	6/56 (10.7)	0.05
Prior AUD (including in remission) or alcohol-related diagnosis,[a] no./total no. (%)	85/152 (55.9)	56/96 (58.3)	29/56 (51.8)	-0.13
Prior AWS diagnosis, no./total no. (%)	40/152 (26.3)	27/96 (20.1)	13/56 (23.2)	-0.11
First CIWA-Ar score, no., median (IQR) [c]	9 (5-16)	10 (6-14)	9 (4-16)	0.02
Severe acute illness (NEWS score ≥ 7), no. (%)	25 (9.8)	17 (11.0)	8 (8.0)	-0.10
Hospital diagnoses, no. (%)				
Arrhythmia	26 (10.2)	14 (9.1)	12 (12.0)	0.09
Brain injury (trauma / hemorrhage / stroke)	10 (3.9)	4 (2.6)	6 (6.0)	0.17
Gastrointestinal tract disorder	32 (12.6)	17 (11.0)	15 (15.0)	0.12
Liver disease	27 (10.6)	18 (11.7)	9 (9.0)	-0.08
Nutrition / electrolyte / acid-base disorder	57 (22.4)	35 (22.7)	22 (22.0)	-0.02
Other substance use disorder (excluding alcohol)	20 (7.9)	9 (5.8)	11 (11.0)	0.19
Psychiatric condition	19 (7.5)	11 (7.1)	8 (8.0)	0.03
Seizure	23 (9.1)	14 (9.1)	9 (9.0)	<-0.01
Sepsis / shock / infection	41 (16.1)	25 (16.2)	16 (16.0)	<-0.01
Trauma	16 (6.3)	6 (3.9)	10 (10.0)	0.24
**Care Delivery**				
Initiated AWS treated within 24h of hospital presentation, no (%)	236 (92.9)	144 (93.5)	92 (92.0)	-0.06
Specialty of prescriber and hospital location at initiation of AWS treatment, no. (%)				0.08

© 2025 Wolpaw BJ et al. *JAMA Network Open*.

ED prescriber in the ED	156 (61.4)	97 (63.0)	59 (59.0)	
Medical prescriber in the ED	30 (11.8)	18 (11.7)	12 (12.0)	
Medical prescriber in acute care	47 (18.5)	27 (17.5)	20 (20.0)	
Surgical prescriber in acute care	2 (0.8)	1 (0.7)	1 (1.0)	
Medical prescriber in the ICU	19 (7.5)	11 (7.1)	8 (8.0)	
Acute care only (no intensive care) during first 24h from hospital presentation, no (%)	192 (75.6)	115 (74.7)	77 (77.0)	0.05

Abbreviations: AWS=alcohol withdrawal syndrome, AUD=alcohol use disorder, CCS=combined comorbidity score, CIWA-Ar=Clinical Institute Withdrawal Assessment for Alcohol-Revised, ED=Emergency Department, EHR=electronic health record, ICU=Intensive Care Unit, IQR=interquartile range, NEWS=National Early Warning System, SMD=standardized mean difference

[a] SMDs were computed as the difference in means standardized by the pooled standard deviation for continuous variables; the difference in proportions standardized by the pooled variance for binary variables; and as an overall measure of distributional differences across categories for categorical variables. Covariates with SMD > 0.15 were included in the multivariable regression models.

[b] Results limited to patients with prior health records: Pre n=96 and Post n=56

[c] 83% of Pre (n=129) and Post (n=83) groups had at least one CIWA-Ar measurement

eTable 5. Use of phenobarbital pre- and post-implementation of the phenobarbital EHR orderset

	Pre-Phenobarbital Orderset	Post-Phenobarbital Orderset
Patients receiving any dose of phenobarbital, no. (%)	52 (33.8)	71 (71.0)
Median (IQR) cumulative phenobarbital, of patients receiving a dose of phenobarbital, mg/kg	3.7 (1.9-10.5)	12.6 (9.7-16.1)

Abbreviations: ED=Emergency Department, ICU=Intensive Care Unit, EHR=electronic health record

eTable 6. Use of the phenobarbital EHR orderset post-implementation by specialty of prescriber and hospital location

No. (%)	No Use of Phenobarbital Orderset	Use of Phenobarbital Orderset
ED prescriber in the ED	13 (39.4)	46 (68.7)
Medical prescriber in the ED	3 (9.1)	9 (13.4)
Medical prescriber in acute care	11 (33.3)	9 (13.4)
Surgical prescriber in acute care	1 (3.0)	0 (0)
Medical prescriber in the ICU	5 (15.2)	3 (4.5)

Abbreviations: ED=Emergency Department, ICU=Intensive Care Unit, EHR=electronic health record

© 2025 Wolpaw BJ et al. *JAMA Network Open*.
