# Supplementary material for: Hospital-Wide Implementation, Clinical Outcomes, and Safety of Phenobarbital for Alcohol Withdrawal
Source: JAMA Netw Open. 2025 Aug 25;8(8):e2528694. doi: 10.1001/jamanetworkopen.2025.28694 (PMC12379078; doi:10.1001/jamanetworkopen.2025.28694)
Supplement: Supplement 2. — Data Sharing Statement [file jamanetwopen-e2528694-s002.pdf]

Data Sharing Statement

Wolpaw. Hospital-Wide Implementation, Clinical Effectiveness, and Safety of Phenobarbital for Alcohol Withdrawal. *JAMA Netw Open*. Published August 25, 2025.
doi:10.1001/jamanetworkopen.2025.28694

Data
Data available: No
